# Covariation among higher-order psychopathology dimensions is driven by nomorbidity and not comorbidity

**DOI:** 10.1038/s44220-026-00727-0

**Published:** 2026-10-01

**Authors:** Ashley L. Watts, Adon F. G. Rosen, Ashley L. Greene, Kevin M. King, Francisco A. C. Meyer, Timothy J. Trull, Kenneth J. Sher, Keanan J. Joyner

**Affiliations:** 1 Department of Psychological Sciences, Vanderbilt University, Nashville, TN USA; 2 Long Island University-Brooklyn, Brooklyn, NY USA; 3 VISN 2 Mental Illness Research, Education and Clinical Center, James J. Peters VA Medical Center, Bronx, NY USA; 4 Department of Psychiatry, Icahn School of Medicine at Mount Sinai, New York, NY USA; 5 Department of Psychology, University of Washington, Seattle, WA USA; 6 Department of Psychiatry, Yale University School of Medicine, New Haven, CT USA; 7 Department of Psychological Sciences, University of Missouri, Columbia, MO USA; 8 Department of Psychology, University of California, Berkeley, Berkeley, CA USA

**Keywords:** Psychology, Psychology, Signs and symptoms

## Abstract

Emerging classification frameworks claim that covariation among psychopathology dimensions reflects comorbidity. We scrutinized this claim by applying Bayesian change point models to four waves of the Adolescent Brain Cognitive Development Study data (*N* = 11,868 youths; 48% female) and examined associations among higher-order psychopathology dimensions. Covariation between psychopathology dimensions was driven by nomorbidity, not comorbidity: we observed strong positive associations among psychopathology dimensions at relatively extreme low (asymptomatic) levels (for example, when three to five symptoms were endorsed) and weak positive associations at higher levels. For instance, at wave 1, a 1-point increase in internalizing was associated with a 32% increase in externalizing at nearly asymptomatic levels (*B* = 0.28), compared with only a 7% increase at higher levels (*B* = 0.07). These findings challenge existing assumptions about comorbidity in nonclinical samples, suggesting that covariation is more accurately explained by the absence of symptomatology rather than shared psychiatric conditions.

## Abstract (web summary)

This research utilized Bayesian change point models on Adolescent Brain Cognitive Development Study data to reveal that psychopathology dimension covariation stems from nomorbidity, indicating stronger associations at low symptom levels compared with higher symptomatology, challenging traditional comorbidity assumptions.

## Main

Psychiatric comorbidity, the co-occurrence of two or more mental disorders, is often regarded as the ‘rule rather than the exception’^1,2^. But epidemiologic data suggest that it is statistically uncommon to be diagnosed with two or more conditions, let alone three or more, at the same time^3^. At a given time, most people in the general population do not exhibit appreciable levels of symptomatology to warrant a psychiatric diagnosis. Around 70% of the population exhibits what we term ‘nomorbidity’—wherein an individual is not diagnosed with a single mental disorder—when past-year diagnoses are assessed, and 50% when lifetime diagnoses are assessed^3^. Further, when followed over long stretches of time, many people (~17–40%) are not diagnosed with a single mental disorder at any time^4,5^. Of course, conditional upon receipt of one diagnosis, comorbidity is far more common. If a person receives one diagnosis, their odds of receiving two diagnoses increases, and so on.

Modern classification systems, such as the Hierarchical Taxonomy of Psychopathology^6^, argue that this ‘rampant comorbidity problem’^7,8^ results from the fact that the Diagnostic and Statistical Manual of Mental Disorders and related systems ‘do not carve nature at its joints’^9–11^. According to this criticism, comorbidity is largely conceivably artefactual, arising because we draw arbitrary borders around phenomena that overlap phenotypically and etiologically^6,12^. In response, these systems harness the supposed overlap among putatively distinct conditions using covariance-based modeling and reorganize psychopathology into a more parsimonious set of dimensions. Products of covariance-based models have, in turn, been widely promoted as capturing sources of psychiatric comorbidity^13,14^.

Across nearly half of a century and thousands of studies, these efforts have converged upon two broad psychopathology dimensions: externalizing and internalizing^6,15–17^, which were first studied in youth^15^ nearly thirty or so years before they were studied in adults^17^. Externalizing encompasses conditions of poor behavioral and emotional control^18^ (for example, conduct disorder, oppositional defiant disorder and substance use disorder), whereas internalizing encompasses conditions that are characterized by intense negative emotions^19^ (for example, generalized anxiety disorder, major depressive disorder and specific phobia). The idea behind these transdiagnostic dimensions is that, say, conduct disorder and substance use disorder exhibit high degrees of comorbidity because they are phenotypically and etiologically overlapping, with disinhibition explaining their overlap^18,20^.

On the basis of the correlation between externalizing and internalizing (*r* ≈ 0.5–0.6), various researchers further proposed that a single dimension^21–23^—the *p*-factor—is the etiologic source of psychiatric comorbidity: ‘a single dimension is able to measure and maybe even explain a person’s liability to mental disorder, comorbidity among disorders, persistence of disorders over time, and severity of symptoms’ (pp. 831^21^; see also refs. ^24–29^). This claim positively asserts that the covariation between externalizing and internalizing reflects some shared substantive mechanism (for example, poor emotional control^30,31^ and negative emotionality^32,33^).

We propose an alternative explanation, that evidence of so-called comorbidity arises owing to the considerable extent of asymptomatic cases in the population. That is, covariation among forms of psychopathology is driven by the inclusion of people who report little to no psychopathology, a typical presentation in large-scale epidemiologic and otherwise community-based samples that are most often used to model hierarchical structures of psychopathology^18,19^.

As the basis of this claim, we borrow from a larger literature that suggests that the inclusion of asymptomatic (or undiagnosed) cases in covariance-based analyses of the structure of psychological constructs encourages their unidimensionality^34^, or inflates the overlap among the phenomena of interest. Including asymptomatic cases produces dense network^35^ structures and coherent (unidimensional) factor^34,36^ structures that—although people assume they capture comorbidity—are driven by people who endorse few, if any, symptoms^37^. That is, what might glue together symptoms is a lack of comorbidity, or nomorbidity (if you find this claim counterintuitive, we expand on our argument in Appendix A).

We extend these findings to examine associations among higher-order psychopathology dimensions, whose covariation is interpreted as being synonymous with psychiatric comorbidity, using innovative statistical modeling techniques. Here, we focus on youth who took part in the Adolescent Brain Cognitive Development Study (ABCD)^38^ given that externalizing and internalizing were first observed among youth^15^ and because epidemiologic data are often used to characterize the structure of psychopathology^6^. The large size of the ABCD Study affords one of the best powered examinations of the interface between higher-order psychopathology dimensions in youth.

## Results

### Univariate and bivariate normality

**Fig. 1:**
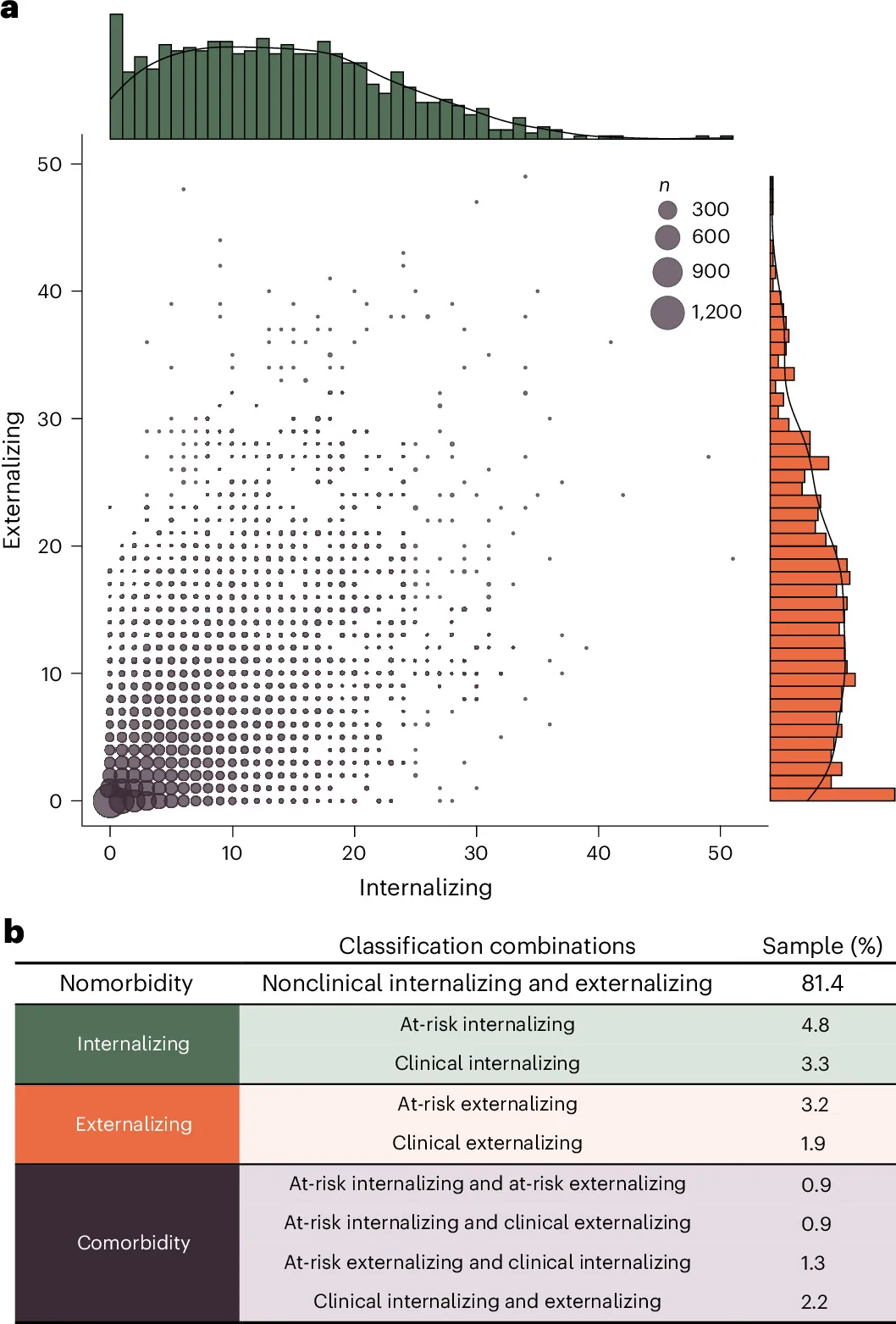
Distributions of externalizing and internalizing across levels of clinical severity. **a**, The bivariate distribution between externalizing and internalizing dimensions, with the marginal distributions for each on their respective axes. The size of the individual points corresponds to the number of cases at each point: the larger the point, the more cases. **b**, The percentage of cases that make up different categories of clinical severity for externalizing and internalizing based on the T-score categorizations for the Child Behavior Checklist. T scores below 60 correspond to nonclinical levels, between 60 and 63 correspond to ‘at-risk’ or borderline levels, and greater than 64 correspond to clinical levels. Supplementary Table C1 (Appendix C) contains a translation between T scores and raw scores for all scales across all waves.

At wave 1, 81% of the sample had nonclinical levels of internalizing and externalizing and only 5.3% of the sample exhibited some combination of at-risk or clinical levels of both internalizing and externalizing (Fig. 1a). All psychopathology dimensions were highly skewed, with a greater weight in the left tail than is expected in a normal distribution (Table 1; see Supplementary Fig. B8 and Supplementary Tables B4–6 for all other waves), kurtotic and not bivariate normal.

**Table 1:** Univariate and bivariate normality for higher-order psychopathology dimensions at wave one

Predictor	Univariate normality		Bivariate normality		
	Skewness	Kurtosis	Criterion	Skewness	Kurtosis
Externalizing	2.30	7.02	Internalizing	18,326	203
			Attention problems	15,085	152
			Thought problems	19,115	211
Internalizing	1.94	5.12	Externalizing	18,326	203
			Attention problems	11,309	116
			Thought problems	16,804	195
Attention problems	1.44	1.83	Externalizing	15,085	152
			Internalizing	11,309	116
			Thought problems	13,436	137
Thought problems	2.26	6.88	Externalizing	19,115	211
			Internalizing	16,804	195
			Attention problems	13,436	137

Univariate skewness values >1 or <−1 indicate a high degree of skewness, and univariate kurtosis values >2 or <−2 indicate substantial nonnormality. Higher estimates of Mardia bivariate skewness and kurtosis indicate deviations from bivariate normality, with 0 indicating true multivariate normality. Bonferroni-corrected two-tailed *P* values associated with the Mardia statistics were significant at *P* ≈ 2.220446 × 10^−16^, indicating that the data were significantly deviant from bivariate normality.

### Associations between externalizing and internalizing

**Fig. 2:**
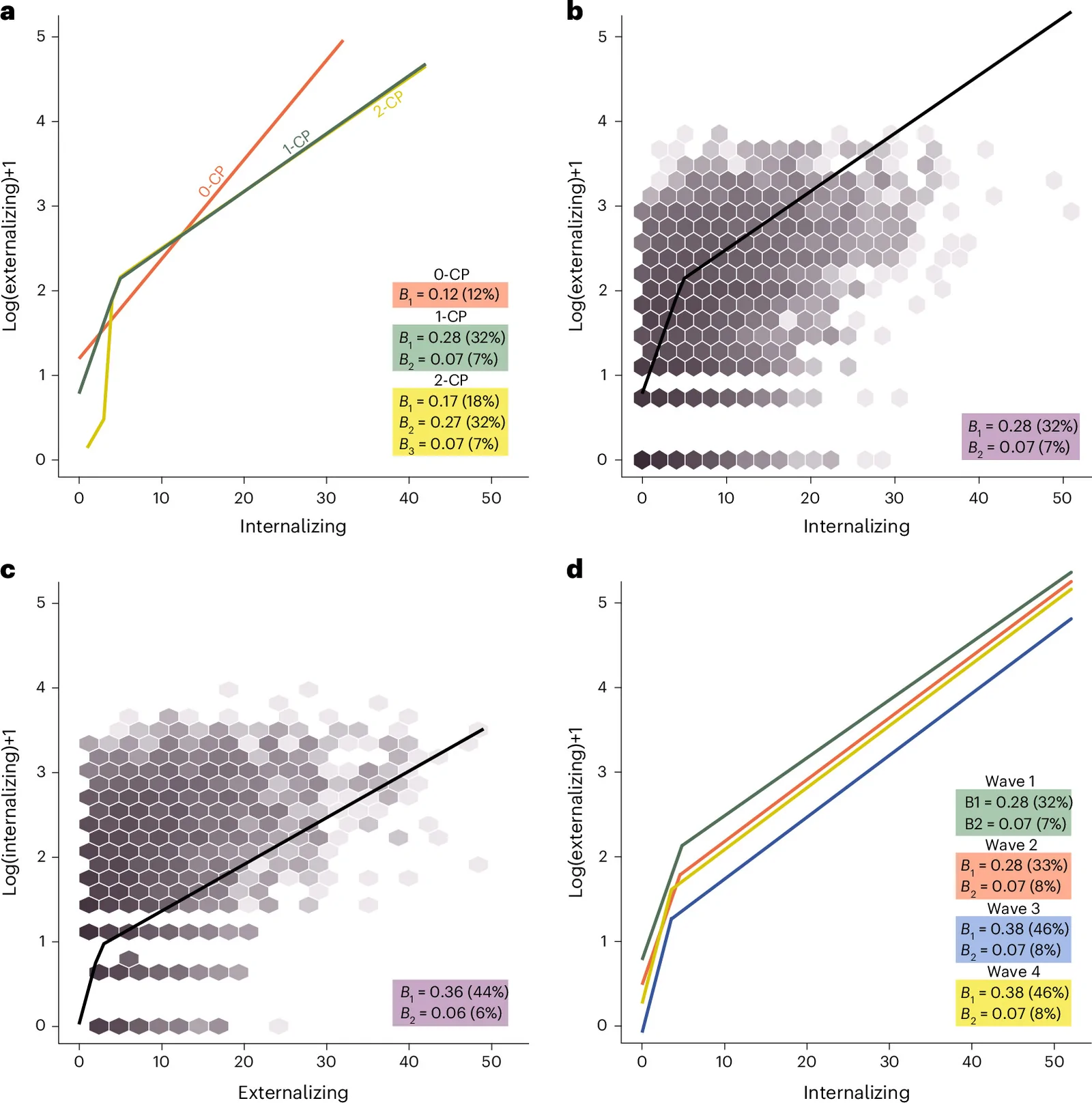
Associations between externalizing and internalizing dimensions from the negative binomial regression models at wave 1. **a**, The association across zero-, one- and two-change point models. **b**, The slopes of the associations from the one-change point model, with the bivariate density overlaid. **c**, The slopes from a one-change point model where internalizing was regressed onto externalizing. **d**, The associations across all waves. The *y* axis reflects log-transformed values of externalizing to facilitate interpretation of the slopes as linear and additive. **b** and **c** depict the density of the cases at each value of externalizing and internalizing in the bivariate distributions—the darker the color, the more cases at that pair of values. *B*_1_ represents the unstandardized beta (slope) coefficient of the first slope estimated, *B*_2_ represents the unstandardized beta (slope) coefficient of the second slope estimated, *B*_3_ represents the unstandardized beta (slope) coefficient of the third slope estimated. The percentages next to the beta coefficients reflect the rate ratios, or the multiplicative factor (percentage) by which the expected count changes when the predictor increases by one point. For Internalizing, values lower than ~9 reflect nonclinical levels (per the T scores), between ~9 and ~14 reflect at-risk levels, and above ~14 reflect clinical levels. For externalizing, values lower than 12 reflect nonclinical levels, between ~12 and ~16 reflect at-risk levels, and above ~16 reflect clinical levels. The specific raw score that corresponds to a T score varies as a function of age and sex. Supplementary Table C1 contains a translation between raw scores and T scores for all scales across all waves. CP, change point.

When we regressed externalizing onto internalizing, the change point occurred when internalizing was equal to a value of 4.85, corresponding to endorsement of approximately two to three internalizing symptoms. The pre-change point slope was strong (*B*_1_ = 0.28), with a 1-point increase in internalizing indicating a 32% increase in externalizing (exp[0.28] of 1.32) (Fig. 2a,b). By contrast, the post-change point slope was relatively weak (*B*_2_ = 0.07) (Fig. 2b), with a 1-point increase in internalizing only indicating a 7% increase in externalizing. The relative rate of the pre-change point slope was 4.6-times larger than that of the post-change point slope (32/7 = 4.6), suggesting far stronger slopes among largely asymptomatic cases who endorse fewer than three symptoms. Thus, nomorbidity, rather than comorbidity, drove the covariance commonly reported in literature.

**Fig. 3:**
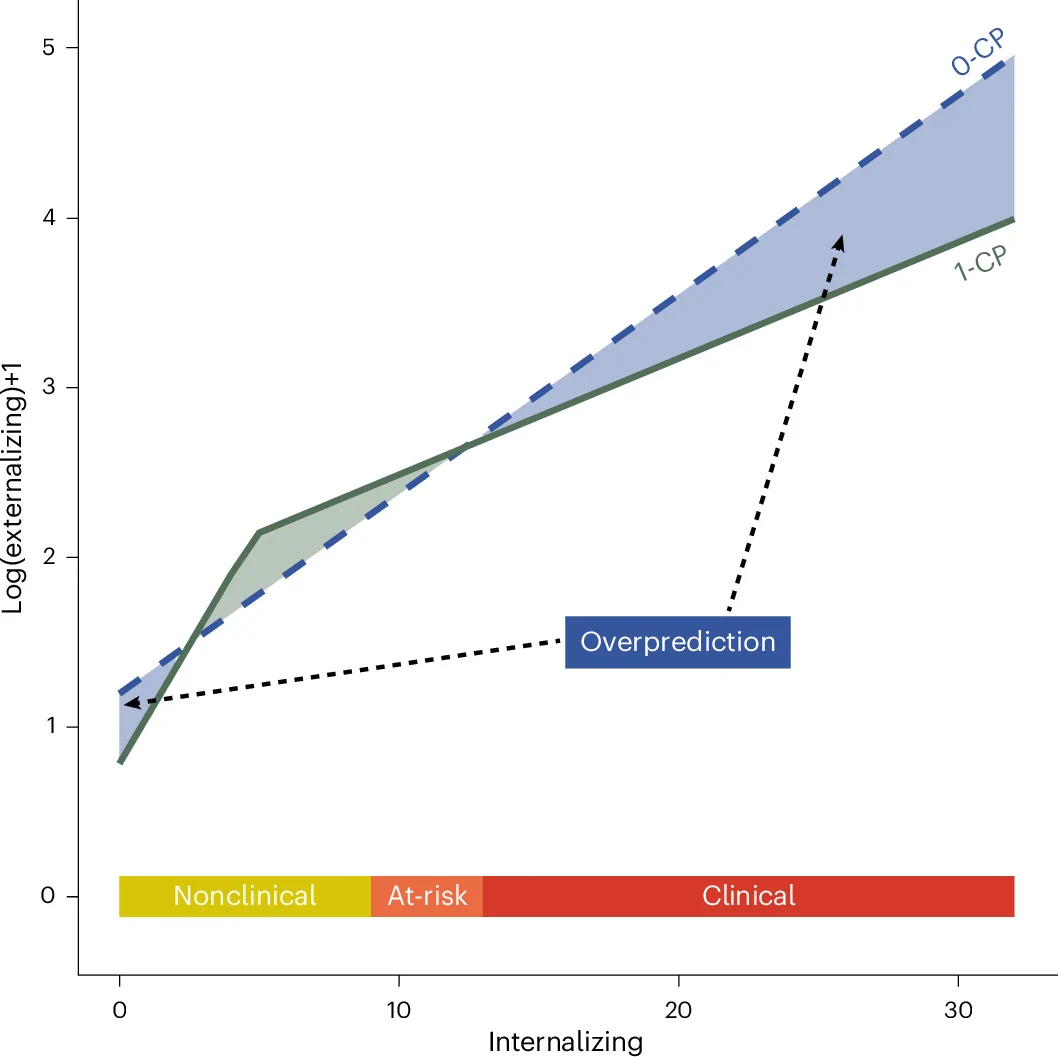
Prediction errors generated by the OLS regression model in the presence of nonlinearity. Here, we depict the same data as presented in Fig. 2a, with an emphasis on the negative binomial models with zero- and one-change points (the dashed blue and solid green lines, respectively), where we regressed externalizing onto internalizing. The zero-change point model corresponds to the OLS regression model most typically used in literature, albeit with a negative binomial link function as opposed to a Gaussian link function. By comparing the slopes from the OLS (zero-change point) and one-change point models, we see that the OLS estimate results in substantial prediction error (per the shaded regions of the plot) across levels of severity for internalizing, with much of the error occurring at clinical levels of severity. Shaded blue regions indicate where the OLS model is overestimating the association relative to what is predicted by the one-change point model, and shaded green regions indicate where the OLS model is underestimating the association. Severity levels are denoted by the bar that ranges from nonclinical levels (yellow) to at-risk levels (orange) to clinical levels (red). For Internalizing, values lower than ~9 reflect nonclinical levels (per the T scores), between ~9 and ~14 reflect at-risk levels, and above ~14 reflect clinical levels (see Supplementary Table C1 for a translation between raw scores and T scores). Thus, because of the considerable extent of nonclinical (and asymptomatic cases) in the data, the OLS estimate generally overstates the statistical association between externalizing and internalizing at nonclinical levels of internalizing, and especially at symptomatic and clinically severe levels of internalizing.

In contrast with the one-change point model, the zero-change point model (that is, the ordinary least squares (OLS) estimate with a negative binomial link function) was associated with considerable prediction error (Fig. 3). The OLS estimate resulted in substantial prediction error, such that it overpredicted the association between externalizing and internalizing at nonclinical levels of internalizing (per the T scores), and particularly at symptomatic and clinical levels of internalizing.

We also considered two robustness checks: (1) the ordering of the predictor and criterion variables, and (2) replication across development. First, these patterns held when we regressed internalizing onto externalizing as opposed to the other way around (Fig. 2c). The change point occurred when externalizing was equal to a value of 2.5, corresponding to endorsement of one to two symptoms. Again, the pre-change point slope (*B*_1_ = 0.36) was far stronger than the post-change point slope (*B*_2_ = 0.06), with the former slope indicating that a 1-point change in externalizing was associated with a 43% increase in internalizing, and the latter indicating a 6% increase; the pre-change point slope was seven-times stronger than the post-change point slope.

Second, these patterns replicated across four waves (Fig. 2d). When we regressed externalizing onto internalizing across each wave, the location of the change points observed shifted slightly but not substantially across waves, with all change points being located between internalizing scores of 3 and 5 (corresponding to endorsement of one to five symptoms) and decreasing over time (*τ*_1_ = 4.85, *τ*_2_ = 4.61, *τ*_3_ = 3.57 and *τ*_4_ = 3.53). The pre- and post-change point slopes were consistent across waves (*B*_1_ = 0.28 (wave 1 and 2), 0.38 (wave 3 and 4) and *B*_2_ = 0.07 (wave 1–4)), with all waves suggesting much stronger slopes at low levels of internalizing. At waves 3 and 4, the pre-change point slopes were even stronger than previous waves, with a 1-point change in internalizing associated with a 46% increase in externalizing. These findings suggest that covariance among psychopathology dimensions may be increasingly driven by asymptomatic cases across development.

### Associations among other higher-order psychopathology dimensions

**Fig. 4:**
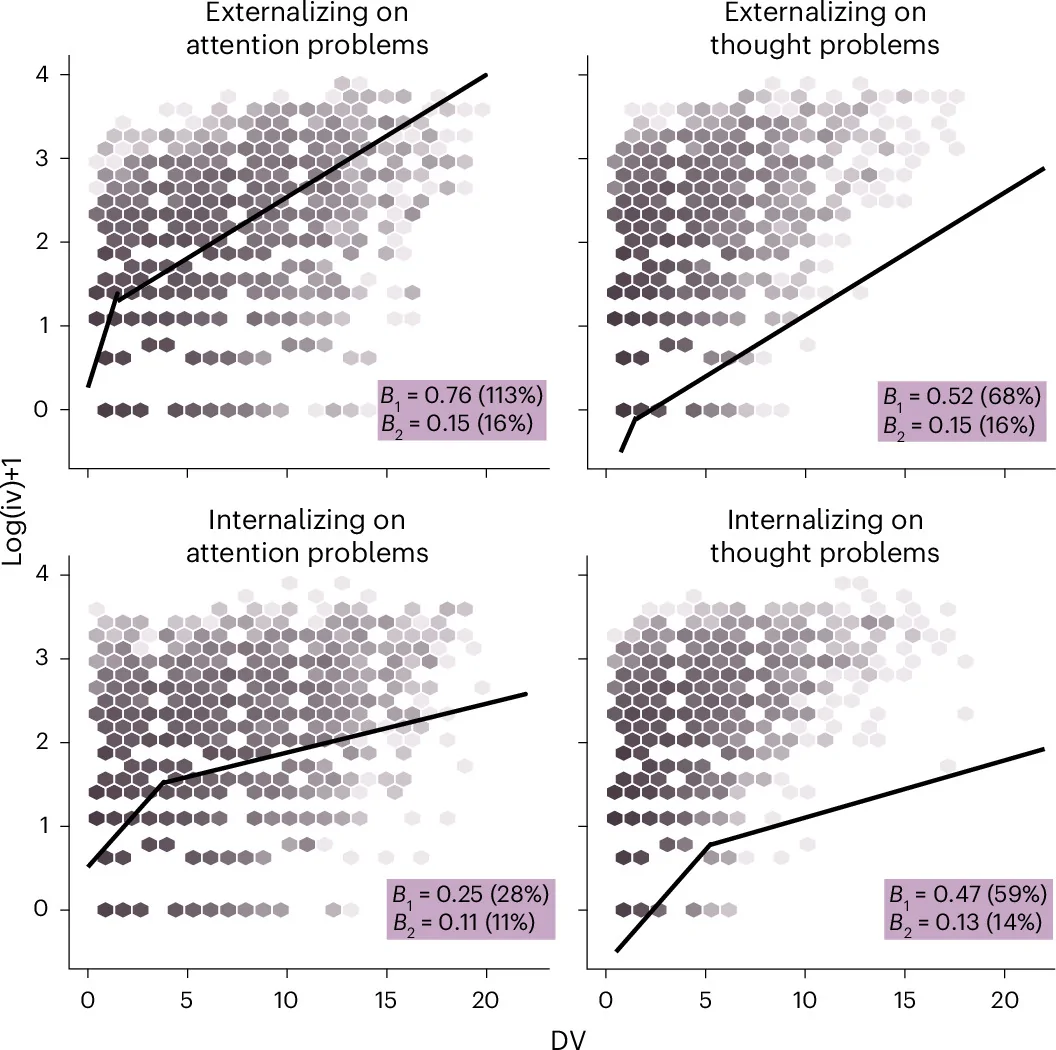
Externalizing and internalizing’s associations with other higher-order psychopathology dimensions from the one-change point negative binomial regression models at wave 1. *B*_1_ represents the unstandardized beta (slope) coefficient of the first slope estimated in change point models. *B*_2_ represents the unstandardized beta (slope) coefficient of the second slope estimated. The percentages next to the beta coefficients reflect the rate ratios or the multiplicative factor (percentage) by which the expected count changes when the predictor increases by one point. In these models, the dependent variable corresponds to the first higher-order dimension listed in the plot title and the independent variable corresponds to the second (for example, ‘attention problems’ is the independent variable in the plot titled ‘externalizing on attention problems’). For externalizing, values lower than 12 reflect nonclinical levels, between ~12 and ~16 reflect at-risk levels, and above ~16 reflect clinical levels. For internalizing, values lower than ~9 reflect nonclinical levels (per the T scores), between ~9 and ~14 reflect at-risk levels, and above ~14 reflect clinical levels. For attention problems, values lower than ~7 reflect nonclinical levels, between ~7 and ~8 reflect at-risk levels, and above ~9 reflect clinical levels. For thought problems, values lower than ~3 reflect nonclinical levels, between ~3 and ~4 reflect at-risk levels, and above ~4 reflect clinical levels. The specific raw score that corresponds to a T score varies as a function of age and sex. Supplementary Table C1 contains a translation between raw scores and T scores for all scales across all waves. DV, dependent variable; IV, independent variable.

We also considered externalizing and internalizing’s associations with other higher-order psychopathology dimensions: thought problems and attention problems (for wave 1 see Table 2 and Fig. 4; see Supplementary Tables B7–B11 for findings from all other waves). We observed the same trend across all models, whereby pre-change point slopes were far stronger than post-change point slopes, including when we regressed externalizing onto attention problems (*B*_1_ = 0.76, *B*_2_ = 0.15), externalizing onto thought problems (*B*_1_ = 0.54, *B*_2_ = 0.15), internalizing onto attention problems (*B*_1_= 0.25, *B*_2_ = 0.11), internalizing onto thought problems (*B*_1_ = 0.47, *B*_2_ = 0.13) and attention problems onto thought problems (*B*_1_ = 0.52, *B*_2_ = 0.12).

**Table 2:** Results from the one-change point negative binomial regressions for all higher-order psychopathology dimensions at wave 1

Predictor variable	Criterion variable	Change point location			Pre-change point slope				Post-change point slope				Ratio of slopes
		*τ*	SE	$\hat{{\boldsymbol{R}}}$	*B*_1_	SE	(%) exp(*B*_1_)	$\hat{{\boldsymbol{R}}}$	*B*_2_	SE	(%) exp(*B*_2_)	$\hat{{\boldsymbol{R}}}$	
Internalizing	Externalizing	4.85	0.07	1.04	0.28	0.00	32	1.05	0.07	0.00	7	1.03	4.5
	Attention problems	3.82	0.05	1.02	0.27	0.00	31	1.03	0.06	0.00	6	1.01	5.0
	Thought problems	5.26	0.06	1.04	0.27	0.00	31	1.03	0.07	0.00	7	1.03	4.3
Externalizing	Internalizing	2.52	0.01	1.00	0.36	0.00	43	1.00	0.06	0.00	6	1.00	7.0
	Attention problems	3.48	0.02	1.01	0.37	0.00	45	1.02	0.06	0.00	6	1.01	7.2
	Thought problems	5.15	0.09	1.07	0.24	0.00	27	1.06	0.06	0.00	6	1.04	4.4
Attention problems	Externalizing	1.50	0.00	1.00	0.76	0.00	114	1.00	0.15	0.00	16	1.00	7.0
	Internalizing	1.07	0.05	1.02	0.25	0.05	28	1.03	0.11	0.00	12	1.02	2.4
	Thought problems	1.51	0.01	1.00	0.54	0.00	72	1.00	0.15	0.00	16	1.00	4.4
Thought problems	Externalizing	2.51	0.01	1.00	0.52	0.00	68	1.00	0.15	0.00	16	1.00	4.2
	Internalizing	2.50	0.00	1.00	0.47	0.00	60	1.00	0.13	0.00	14	1.00	4.3
	Attention problems	2.51	0.01	1.00	0.52	0.00	68	1.01	0.12	0.00	13	1.01	5.3

*B*, unstandardized beta (slope) coefficients; exp(*B*_1_), exponentiated beta coefficient; $\hat{{R}}$, scale reduction factor (convergence criterion); SE, standard error; *τ*, the change point location. The exponentiated beta coefficients reflect the percent increase in the criterion variable per point of the predictor variable; for instance, when we regressed externalizing onto internalizing, *B*_1_ = 0.28, so exp(*B*_1_) = exp(0.28) = 1.32, or a 32% increase. The ratios of slopes are computed as follows: % exp(*B*_1_) / % exp(*B*_2_). Table C1 contains a translation between raw scores and T scores for all scales across all waves.

## Discussion

Researchers commonly make the inference that the correlation between two higher-order psychopathology dimensions captures comorbidity^16,21,39^. Nevertheless, we provide empirical evidence here that covariation among psychopathology dimensions is largely driven by nomorbidity, or a lack of comorbidity. Associations between psychopathology dimensions were strong and positive among asymptomatic people and substantially weaker among people who experience more than a handful of symptoms with relative frequency. That is, psychopathology dimensions were more differentiated (or less overlapping) at higher severity levels, to the extent that associations were negligible at moderate to clinically severe levels of psychopathology. Thus, covariance among psychopathology dimensions does not necessarily reflect or capture comorbidity^16,21,40^. Our findings underscore important limitations of covariance-based analyses for studying comorbidity, the *p*-factor^41–44^ and the latent structure of psychopathology more generally.

Covariance-based similarity measures are dramatically influenced by the distributional qualities (for example, base rates and skewness) of the variables of interest^45^, an issue that has been better appreciated in other fields (for example, ecology, computer science and engineering). Ecology, for instance, relies on association measures that exclude the mutual absences of rare species’ locations^46^ for the very reason we demonstrate here: traditional association measures (such as the correlation or factor analysis^34^) erroneously indicate that two species, or forms of psychopathology, are highly related simply because they do not co-occur often. The use of association measures that are driven by the absence of relatedness is particularly relevant to textbook definitions of comorbidity, which specifically emphasize a shared presence—rather than shared absence—of two or more disorders^47^. Covariance-based analyses, which include factor analyses of psychopathology data^6,16,48^, in the general population may largely characterize the absence of different forms of psychopathology rather than their co-occurrence (or comorbidity).

A construct whose statistical and ontological status is particularly challenged by our findings is the *p*-factor, a dimension that is claimed to give rise to all forms of psychopathology and explains why different forms of psychopathology are associated with each other^21,22^. So it goes, the higher one’s *p*-factor score, the higher propensity to develop several forms of psychopathology^21,22^. Critically, our findings suggest that the *p*-factor appears largely irrelevant to clinical levels of psychopathology and the subpopulation of people who experience psychopathology in some appreciable form, which is inconsistent with the notion that it is a unitary dimension on which all humans can be placed^21,22^.

Common interpretations of the *p*-factor as the source of psychiatric comorbidity thus appear to rest on a faulty inference because the *p*-factor may not reflect comorbidity, but rather, an absence of clinically significant psychopathology. In this way, the *p*-factor appears more relevant to low as opposed to moderate or high levels of psychopathology. Although this conclusion may appear overly conjectural, related literature supports it. According to Spearman’s law of diminishing returns (also termed ‘cognitive ability differentiation’), the general factor of intelligence (or *g*)^49^, the supposed theoretical predecessor to the *p*-factor^21,50^, contains more explanatory power at lower as opposed to higher levels of intelligence^51–54^.

Our findings also have implications for structural models of psychopathology that form the bedrock of modern classification efforts^6^. Such models, which typically rely on the general linear model, assume rather than test linear (or additive) relationships among psychopathology variables. Factor analysis, which is most commonly used to construct taxonomic frameworks^6,55^, actually makes even stronger assumptions about linearity—that a latent normal distribution underlies the data^56^, that the statistical relationship between a given item and the latent factor is linear, and that the correlations among latent factors are linear^6,57–59^. Even though factor analysis assumes an underlying normal distribution, estimated factor scores are not required to be normally distributed. In fact, in these data, factor scores of psychopathology dimensions were highly skewed and kurtotic, often even more so than the sum scores (Supplementary Fig. B9 and Supplementary Tables B12–15), and assumptions of linearity between psychopathology dimensions were violated in the data considered here (Supplementary Figs. B9 and B10).

Given apparent violations of linearity, when researchers implicitly or explicitly adopt a model with one slope in the population (that is, a zero-change point model), the slopes simply capture the approximate average of the two slopes estimated in a one-change point model (Appendix D). Although it may be tempting to think that the ‘average’ is a sufficient guess for the typical person in the population, that average has no psychological meaning when there are substantially different slopes in the population. Such an estimate is not only an oversimplification of the true population relationship, but a biased estimate of it^56,60–64^. Specifically, we showed that these estimates are upwardly biased, over characterizing the statistical association between higher-order psychopathology dimensions at nonclinical levels of psychopathology, and particularly at clinical levels. Because the assumptions of the linear model were extremely violated in these data (Supplementary Figs. A9 and A10), we conducted a simulation study (Appendix D) to show that the linear model produces a biased, and often extremely misleading, estimate of the population association between two psychopathology dimensions in the presence of nonlinearity under many conditions.

Ultimately, there may be no single, ‘true’ structure of psychopathology in the population^65,66^. Instead, the assumption of population homogeneity may be inappropriate^66^, and there may be discrete subpopulations of people that are differentially susceptible to psychopathology, have differential comorbidity patterns and, in turn, have dramatically different structures of psychopathology. Related literature supports such a possibility: self-esteem appears to have different factor structures—one unidimensional and one multidimensional—depending on the subpopulation of interest^67^. Further, the structure of intelligence appears moderated by ability level, with the strength of correlations among intelligence tasks decreasing as intelligence levels increase^68–70^.

With the considerable degree of asymptomatic cases in most large-scale epidemiologic samples in mind, it may be the case that there is a large class with a relative absence of diagnosed psychopathology for whom psychopathology appears to conform to a unidimensional solution^35,36,71^, and one or more relatively symptomatic classes for which psychopathology appears multidimensional. We suspect that there is a subpopulation that comprises people with an exceptionally high threshold to experience and/or accurately report psychopathology, whose detection is obscured—even precluded—in most modern classification efforts^72^ owing to the widespread assumption of population homogeneity^73^.

As the framers of the Diagnostic and Statistical Manual of Mental Disorders (DSM-6) are charged with capturing the continuum (or dimensionality) of mental health, and to explicitly consider severity and transdiagnostic features^74^, our findings are highly relevant to current efforts to incorporate dimensionality into future psychiatric nosology^9,14^. In particular, our findings suggest that dimensional structures of psychopathology should be evaluated in relation to their target population and intended clinical use^75^. A structure estimated from covariance in a community sample may be useful for population screening or describing overall symptom burden, but it should not automatically be interpreted as evidence of clinical comorbidity, shared etiology or treatment-relevant co-occurrence among symptomatic patients. In community samples overrepresented by asymptomatic cases, global covariance may primarily reflect shared absence of symptoms. Thus, studies used to support dimensional nosology should report the distributional qualities of the data (for example, skewness and kurtosis), confirm that assumptions of the general linear model are met in their data, examine nonlinear associations among forms of psychopathology and evaluate whether broad structures remain informative in the clinical range. Our data suggest that the general linear model results in overprediction of covariance (or so-called comorbidity) among psychopathology dimensions at clinically severe levels of psychopathology.

Given the strong implications of our findings, it is worth discussing several potential limitations or criticisms of them. First, because we examined nonlinear relationships among higher-order psychopathology dimensions, skeptics of our findings might argue that we have invoked Berkson’s bias^76^ or the like^77,78^ by restricting the sample post-change points to reflect higher degrees of severity (that is, conditioning on severity), thereby attenuating the associations between psychopathology dimensions at higher severity levels. That argument holds only if there is a single linear relationship between two psychopathology dimensions of interest in the population, or an absence of nonlinearity and population heterogeneity^73^ (Appendix D). It is also worth noting that our change points were consistently located at extremely mild levels of psychopathology (for example, when two or three symptoms were endorsed), meaning that our results are not artifacts of restricting the sample to high severity levels.

Second, because we allowed for a change point in the associations between higher-order psychopathology dimensions, skeptics might argue that we artificially produced different slopes and, in turn, evidence of nonlinearity. Change point models do not inherently produce evidence of nonlinearity. Indeed, our simulation study (Appendix E) demonstrates that the change point model will generate estimates identical to the linear model when there is no evidence of nonlinearity. We also observed evidence of this fact in our analysis of psychopathology data. For instance, in the Gaussian one-change point model that regressed externalizing onto internalizing (Appendix C), the pre- and post-change point slopes were almost identical to each other, and to the slope estimated in the zero-change point model. Thus, it is not always the case that modeling a change point will engender evidence of nonlinearity.

Third, we relied on sum scores of youth psychopathology data. In sensitivity analyses, we examined caregivers’ psychopathology data from the Adult Self-Report, the adult counterpart to the Child Behavior Checklist (CBCL), and our findings mirrored those reported here (Supplementary Table C9). Change point models indicated a preference for one (as opposed to zero or two) change points, and the pre-change point slopes were considerably stronger than the post-change point slopes. It is perhaps unsurprising that our findings replicated among adults because the adult data in the ABCD Study were highly skewed and kurtotic (Supplementary Fig. B11 and Supplementary Tables B16 and B17). As demonstrated in our simulation study (Appendix E), any data are liable to the problems we demonstrated here: the associations among higher-order psychopathology dimensions—in youths and adults—are driven up with the inclusion of asymptomatic cases.

With that said, here, we focused only on the associations among higher-order psychopathology dimensions and did not conduct factor analyses of psychopathology, which is typical in classification efforts (see Appendix C for subscale-level analyses). As yet, there is no implementation of the Bayesian change point model for factor analysis. Still, the sum score is a special case of a latent variable^79^, so our findings bear directly on factor models and the interpretation of correlations among higher-order psychopathology factors as evidence of comorbidity.

Fourth, given the highly positively skewed nature of our psychopathology dimensions, the ABCD data may be an inadequate representation of psychopathology at clinically severe levels. We offer several counterpoints to this potential conclusion. Although nonclinical cases reflected the plurality of data in the ABCD Study (Fig. 1), we had an appreciable amount of clinically severe levels of externalizing and internalizing, with 2.2% of youths (*n* = 257) falling into this category. Moreover, when we forced two-change point models, we did not find a change point at severe levels of psychopathology dimensions and did not find evidence of increasingly strong associations at severe levels (Appendix C). In one instance, when we regressed externalizing onto internalizing in the adult data, we detected a second change point at a score of 30 (Supplementary Table C9). Still, the three slopes detected in this model suggested a decreasing association between externalizing and internalizing as a function of severity (*B*_1_ = 0.29, *B*_2_ = 0.12 and *B*_3_ = 0.04).

Along those lines, we used an alternate estimation method, generalized additive multilevel models (Appendix C), that may be more sensitive to the upper tail of the distribution, and still found no support for this hypothesis. Thus, we are not convinced that our two-change point models were preordained to reject the notion that higher-order psychopathology dimensions become more strongly associated at severe levels^21^. Instead, there was no uptick for the third estimated slope, ruling out the possibility that comorbidity ‘emerges’ at severe levels of psychopathology.

We welcome investigations of nonlinearity among samples with more representation of severe levels of psychopathology, such as in clinical or mixed community–clinical samples, and differing assessments of psychopathology, including ones that assess finer-grained phenomena than we consider here. Although not of focus here, it is possible that the dimensionality of narrower phenomena is also driven by asymptomatic cases, and that higher-order psychopathology dimensions may appear more multidimensional at higher levels of psychopathology. For instance, perhaps depression and anxiety are most overlapping at low levels and less overlapping at higher levels (Appendix C).

Mitigating potential generalizability concerns, a recent meta-analysis^80^ indicates that there is strong reason to believe that higher-order psychopathology dimensions become more weakly—as opposed to more strongly—associated at higher levels of severity. The correlation between externalizing and internalizing is dramatically stronger in nonenriched (or more nomorbid) samples (*Φ* = 0.48) compared with psychopathology enriched samples (or samples with more comorbidity, *Φ* = 0.20). This pattern holds for assessment strategies that manipulate the extent of positive skew or nomorbidity in the data^35,36,71^; the correlation between externalizing and internalizing was much stronger when current diagnoses (*Φ* = 0.37), as opposed to lifetime diagnoses (*Φ* = 0.19), were modeled, and it was also much stronger when studies did not rely on hierarchical exclusion (or ‘skip-out’) rules (*Φ* = 0.41) compared with those that did (*Φ* = 0.24)^81^. Compared with their counterparts, current diagnoses and diagnoses that did not rely on hierarchical exclusion rules are more strongly skewed^82,83^ and hence contain more asymptomatic cases^36,84^.

## Conclusion

Our findings challenge the prevailing assumption that statistical covariance among forms of psychopathology should be taken as evidence of psychiatric comorbidity. Just as correlation does not imply causation, correlation does not imply comorbidity. The inclusion of asymptomatic, or nomorbid, cases in covariance-based analyses of psychopathology created the illusion of strong covariation among higher-order psychopathology dimensions. At higher levels of psychopathology, higher-order psychopathology dimensions were far more differentiated than is implied by the existing literature^16,17,85,86^, providing weak evidence of a *p*-factor (or general factor of psychopathology). Ignoring the implications of the inclusion of largely asymptomatic cases in psychopathology research could jeopardize the integrity and utility of psychiatric classification systems^6,9,14,74^.

More generally, we call for a reconsideration of the uncritical adoption of the general linear model in psychopathology research, which often produces comorbidity-inference artifacts in community-level data, where most individuals do not exhibit clinically severe psychopathology. We encourage researchers to embrace nonlinearity and population heterogeneity and adopt conceptual and analytic frameworks that prioritize symptomatology over statistical correlation. An exaggerated sense of comorbidity among distinct forms of psychopathology has, and will continue to, perpetuate the reification of artifactual transdiagnostic dimensions^42,87,88^ and cease investigation of disorder-specific mechanisms^21^, fundamentally impeding the field’s detection of psychopathology’s etiologic mechanisms, treatment targets and consequences.

## Methods

### Data

Data were drawn from the first four waves of ABCD (data release 5.0), a collaboration between 21 sites across the USA^38,89^ that tracks a convenience sample of 11,868 nine- and ten-year-olds from the USA annually across development (wave 1: *M*_age_ = 9.94 years, s.d. 0.62 years; 48% female). The ABCD data are not nationally representative, but the study team endeavored to make the sample as representative of the US population as possible. Overall, 74% of youth participants identified as white (14% Hispanic), 21% as Black (2% Hispanic), 7% as Asian and 11% as ‘other’ (see Supplementary Table B1 in Appendix B for demographics of all other waves). In total, 20% identified as Hispanic. These data are available to researchers upon receipt of a data use agreement from the National Institutes of Health (https://nda.nih.gov/).

### Measures

We used the caregiver-reported CBCL^90^, a 113-item assessment of psychiatric symptoms. The CBCL is among the most widely used broadband assessments of youth psychopathology, with over 10,000 publications reporting its use across over 50 different countries^91^. Symptoms are rated on a three-point scale (‘not true’, ‘somewhat true/sometimes true’ and ‘very true/often true’). We used summary score composites for all psychopathology variables. For readers interested in T scores, Supplementary Table C1 (Appendix C) contains a translation between raw scores and manual-based T scores for all scales across all waves. Of primary focus here were the two broadband psychopathology scores, externalizing and internalizing (at wave 1, coefficient omegas were 0.89 and 0.87, respectively) (see Supplementary Table B2 for omegas for all other waves).

We also considered two subscales that contain items not included in externalizing and internalizing: attention problems and thought problems (coefficient omegas were 0.86 and 0.70, respectively), because they map onto broad transdiagnostic dimensions in modern classification frameworks^18,92^. In addition to externalizing and internalizing spectra, modern frameworks also include neurodevelopmental^93^ and psychosis^92^ (or thought disorder) spectra, which map broadly onto attention problems and thought problems, respectively. As the field incorporates lesser studied higher-order psychopathology dimensions into hierarchical frameworks of psychopathology, we felt that broadening our focus to include these dimensions would be of relevance to the field.

Readers interested in the interface among lower-order psychopathology dimensions within a higher-order dimension (that is, anxiety and depression within internalizing) are directed to Supplementary Table C8 in Appendix C, where we show the results for all change point models based on CBCL subscales. In brief, results were consistent with what we report here, with a few exceptions: across models, the one-change point model was superior to others, and pre-change point slopes were far stronger than post-change point slopes.

### Analyses

#### Univariate and bivariate skew and kurtosis

First, we estimated univariate skewness and kurtosis for all psychopathology dimensions to inform deviations from a normal distribution. A normal distribution’s skewness is 0, with positive values indicating positive skew, and its kurtosis is 3, with values greater than 3 suggesting leptokurtosis. Second, we examined bivariate skewness and kurtosis^84^, which inform how bivariate relationships deviate from a theoretical multivariate normal distribution. For bivariate estimates, we report Mardia’s chi-squared tests, whose *P* values indicate whether the data satisfy the assumptions of multivariate normality. We considered all unique pairwise comparisons (that is, six) among psychopathology dimensions across all four waves, and Bonferroni corrected all *P* values to penalize for the number of tests conducted (24, or 6 × 4).

#### Bayesian change point models

We used Bayesian change point models to examine whether the relationship between psychopathology dimensions changes at different points along their joint distribution, allowing for nonlinear relationships. Change point models have other common names, including broken-stick models^94^, or turning point^95^ or break point models^96^. They are used routinely in ecology to identify transitions in animal behavior^97^ and environmental thresholds^98^ in cross-sectional data, as well as in time-series analysis^99^. They are conceptually similar to regression discontinuity designs^100^.

The Bayesian change point model identifies locations, or change points, along the predictor variable where its relationship with the criterion variable may change. For instance, the relationship between *x* and *y* might be strong and positive before ten units of *x*, at which point a change point occurs, and the relationship may become weak and negative after ten units of *x*. By contrast, an OLS regression assumes that the relationship is equivalent, or does not change, across the joint distribution (in Appendix D we show, via a simulation study, that the OLS estimate is biased in the presence of nonlinearity).

The number of change points can range from zero to infinity. A one-change point model adheres to the following structure:

$${y}_{i}=\left\{\begin{array}{cc}{\alpha }_{1}+{\beta }_{1}X, & x < {\tau }_{r}\\ {\alpha }_{2}+{\beta }_{2}X, & x\ge {\tau }_{r}\end{array}\right.,$$

where *τ*_*r*_ represents the location of the change point (or the value of the predictor variable where the slope changes) and where the *α* and *β* terms are the intercept and slope terms, respectively. That is, the one-change point model is a combination of two generalized linear models, with each model estimating an intercept and slope before and after the change point. Because the change point model is a generalized linear model, it can be extended to other extensions of the generalized linear model (for example, hierarchical and generalized linear models).

##### Model estimation

We allowed for up to two change points and compared two link functions, the Gaussian link and the negative binomial link. We used the Gaussian link given its overwhelming popularity, and we used the negative binomial link because it should be more appropriate given the positively skewed (or overdispersed) count data used here^101,102^.

In contrast with the Gaussian link-based regression, negative binomial regression assumes a nonlinear (log–linear) relationship between the predictor variables and the expected count of the criterion variable, whereby change in the predictor is multiplicative. The beta coefficient (or slope, *B*) represents the change in the log of the expected count for a 1-point increase in the predictor variable. To translate the nonlinear estimates, one can exponentiate the slope (exp[*B*]) so that it reflects the rate ratio or the multiplicative factor by which the expected count changes when the predictor increases by one point.

Every model used Markov Chain Monte Carlo sampling with a burn-in length of 10,000, a total chain length of 20,000 and a thinning of every second sample to control for potential autocorrelation. All sampling was performed using the Stan language and the No-U-Turn Sampler algorithm^103,104^. We selected diffuse and naive priors. Estimation of the change point(s) used uniform priors that ranged from the minimum to the maximum value of the domain, and estimation of the slope(s) and intercept(s) used normal priors centered on 0 with a variance of 1. Random effect variance used uniform priors ranging from 0 to 10. In terms of other covariates, we included a fixed effect for age and nested random effects for complex survey design features (family within site).

##### Model convergence

We examined model convergence using the potential scale reduction factor $\hat{R}$ (ref. ^105^) to determine if the chains mixed. $\hat{R}$ measures the ratio of the average variance of samples within each chain to the variance of the pooled samples across chains. If all chains are at equilibrium, the average variance of samples within each chain will be equal to the variance of the pooled samples across chains, producing an $\hat{R}$ of 1.0. Values less than 1.2 generally indicate convergence across chains^105^. To confirm strong model convergence, we also visually inspected the trace, density and autocorrelation plots (Supplementary Figs. B1–B4; Supplementary Table 2 contains $\hat{R}$ values for the slope parameters of interest here; and Appendix C contains all model parameters, including $\hat{R}$ values, for all models).

##### Model comparison

We prioritized the Leave-One-Out Information Criterion (LOOIC), which is commonly used for Bayesian model selection^106^. LOOIC facilitates the comparison of models within and across link functions, with lower values indicating a better-fitting model. We also used model *R*^2^ (or the proportion of variability explained).

Of the 288 models compared—Gaussian and negative binomial, each with either zero, one or two change points—LOOIC supported one-change point negative binomial models over Gaussian models (Supplementary Fig. B5 and Supplementary Table B3). Additional sources of evidence converged on the inappropriateness of the Gaussian models, including that the data were not univariate or bivariate normal, and that model diagnostics of linear regressions suggested substantial violations of linear regression assumptions, including linearity, homogeneity of variance and homogeneity in residuals (Supplementary Figs. B9 and B10). Thus, results from the Gaussian models are invalid.

Among the negative binomial models, the two-change point models were over extracted, with little to no increment in *R*^2^ above and beyond the one-change point models (Supplementary Fig. A7). For instance, when we regressed externalizing onto internalizing at wave 1, model *R*^2^ (or variance explained in externalizing) was 21% in the zero-change point model, 56% in the one-change point model and 55% in the two-change point model.

We anticipated that a two-change point model might reveal a change in the slope between internalizing and externalizing at high levels of internalizing (or high levels of severity), with the slope strengthening and reflecting increased comorbidity. Instead, the second change point in this model was located at *τ*_2_ = 5.09, corresponding to endorsement of around three or four internalizing symptoms. Further, the slope after the second change point was lower than the slope after the first change point (*B*_1_ = 0.17, *B*_2_ = 0.27 and *B*_3_ = 0.07, respectively), which is inconsistent with the notion that their association would increase as a function of presumed comorbidity. There were no instances in the two-change point negative binomial models where the slope after the second change point (*B*_3_) increased relative to the prior slope (*B*_2_). Given the lack of incremental *R*^2^ for the two-change point models, we carried forward all one-change point negative binomial models (Fig. 2a).

We considered an alternative estimation method, generalized additive multilevel models^107,108^, to examine nonlinear associations between higher-order psychopathology dimensions, as in previous work^109^. We detected the same nonlinear associations using this method and did not find consistent evidence that there is a strong positive association between externalizing and internalizing at extreme high levels of internalizing (Appendix E).

### Reporting summary

Further information on research design is available in the Nature Portfolio Reporting Summary linked to this article.

## Supplementary information

Supplementary Information Appendices A, B, D and E.

Reporting Summary

Peer Review File

Supplementary Table 1 These tables comprise Appendix C, which contains nine tables along with a table of contents.

## Data Availability

Data used in the preparation of this article were obtained from the ABCD Study (https://abcdstudy.org), held in the NIMH Data Archive. This is a multisite, longitudinal study designed to recruit more than 10,000 children aged 9–10 and follow them over 10 years into early adulthood. The ABCD Study is supported by the National Institutes of Health and additional federal partners under award nos. U01DA041048, U01DA050989, U01DA051016, U01DA041022, U01DA051018, U01DA051037, U01DA050987, U01DA041174, U01DA041106, U01DA041117, U01DA041028, U01DA041134, U01DA050988, U01DA051039, U01DA041156, U01DA041025, U01DA041120, U01DA051038, U01DA041148, U01DA041093, U01DA041089, U24DA041123 and U24DA041147. A full list of supporters is available at https://abcdstudy.org/federal-partners.html. A listing of participating sites and a complete listing of the study investigators can be found at https://abcdstudy.org/consortium_members/. ABCD consortium investigators designed and implemented the study and/or provided data but did not necessarily participate in the analysis or writing of this report. This manuscript reflects the views of the authors and may not reflect the opinions or views of the NIH or ABCD consortium investigators. The ABCD data repository grows and changes over time. The ABCD data used in this report are available via the NDA at https://doi.org/10.15154/8873-zj65 (ref. ^110^). These data are available to researchers upon receipt of a data use agreement from the National Institutes of Health (https://nda.nih.gov/).

## Code availability

Our results are fully reproducible (upon receipt of the ABCD data) via our code, which is avilable via OSF at https://osf.io/nr5h3/overview and via GitHub at https://github.com/Watts-MAP-Lab/psychopathology_nonlinearity.
