# Supplementary material for: Covariation among higher-order psychopathology dimensions is driven by nomorbidity and not comorbidity
Source: Nat Ment Health. 2026 Oct 1;4(11):1674–85. doi: 10.1038/s44220-026-00727-0 (PMC13736491; doi:10.1038/s44220-026-00727-0)
Supplement: Supplementary file 1 [file 44220_2026_727_MOESM1_ESM.pdf]

nature mental health

Supplementary information https://doi.org/10.1038/s44220-026-00727-0

Covariation among higher-order psychopathology dimensions is driven by nomorbidity and not comorbidity

In the format provided by the authors and unedited

This document contains Appendices A, B, D, and E. Appendix C is shared as a separate file.

Appendix A: A description of the tension between the general linear model and inferences about comorbidity in large-scale community-level data

Appendix B: Supplemental Tables

Appendix D: A simulation study to illustrate how the OLS is biased when there is a nonlinear relationship between two variables

Appendix E: Alternative Estimation Method

APPENDIX A

A description of the tension between the general linear model and inferences about comorbidity in large-scale community-level data

These two statements may seem contradictory:

1. People are statistically more likely to be diagnosed with a third mental disorder if they are diagnosed with two.
2. Covariance between mental disorders is driven by nomorbidity, or a lack of comorbidity.

Here, we will explain the tension between these two statements, and why the general linear model in community-level data is less inclined to capture comorbidity *per se*. The linear model's likelihood is dominated by the highest-density part of the data (i.e., where most observations lie). In other words, the region of the data space with the most cases drives the estimated relationship.

Under this assumption, each observation contributes a normal density:

$$p(y_i \mid x_i, \beta, \sigma^2) = \frac{1}{\sqrt{2\pi\sigma^2}} \exp\left(-\frac{(y_i - x_i^\top \beta)^2}{2\sigma^2} \right).$$

Ordinary least squares is the maximum-likelihood estimator when errors are Gaussian:

$$y_i = x_i^\top \beta + \varepsilon_i, \varepsilon_i \sim N(0, \sigma^2).$$

With Gaussian errors, we maximize the log-likelihood of the relationship between two variables, β, which is equivalent to minimizing the sum of squared residuals (the goal of OLS regression). The log-likelihood (up to a constant) is:

$$\ell(\beta, \sigma^2) = -\frac{n}{2} \log(2\pi\sigma^2) - \frac{1}{2\sigma^2} \sum_{i=1}^{n} (y_i - x_i^\top \beta)^2.$$

So, maximizing the likelihood in β is equivalent to minimizing the residual sum of squares (RSS):

$$\text{RSS}(\beta) = \sum_{i=1}^{n} r_i^2, r_i = (y_i - x_i^\top \beta).$$

A key feature of the Gaussian log-likelihood (and the objective of OLS regression) is that it is an unweighted sum over observations:

$$\text{RSS}(\beta) = \sum_{i=1}^{n} r_i^2.$$

Each observation contributes additively to model estimation. The linear model's likelihood is dominated by the highest-density part of the data (i.e., where most observations lie). Thus, the parts of the data space where observations are most common contribute most to the objective function of the estimator.

In simpler terms, OLS “listens” most to where most of the data are. Just like the average of eight numbers {3, 3, 3, 3, 3, 3, 1, 8} will end up close to 3 if six of them are 3 (mean = 3.375), the regression fit is pulled toward the part of the outcome–predictor space where most observations lie (here, the largely asymptomatic majority).

In many psychopathology studies, most individuals are at the low end of the distribution. Because of the unipolar nature of psychopathology assessments, most individuals are located at the center (or mean) of the outcome distribution.

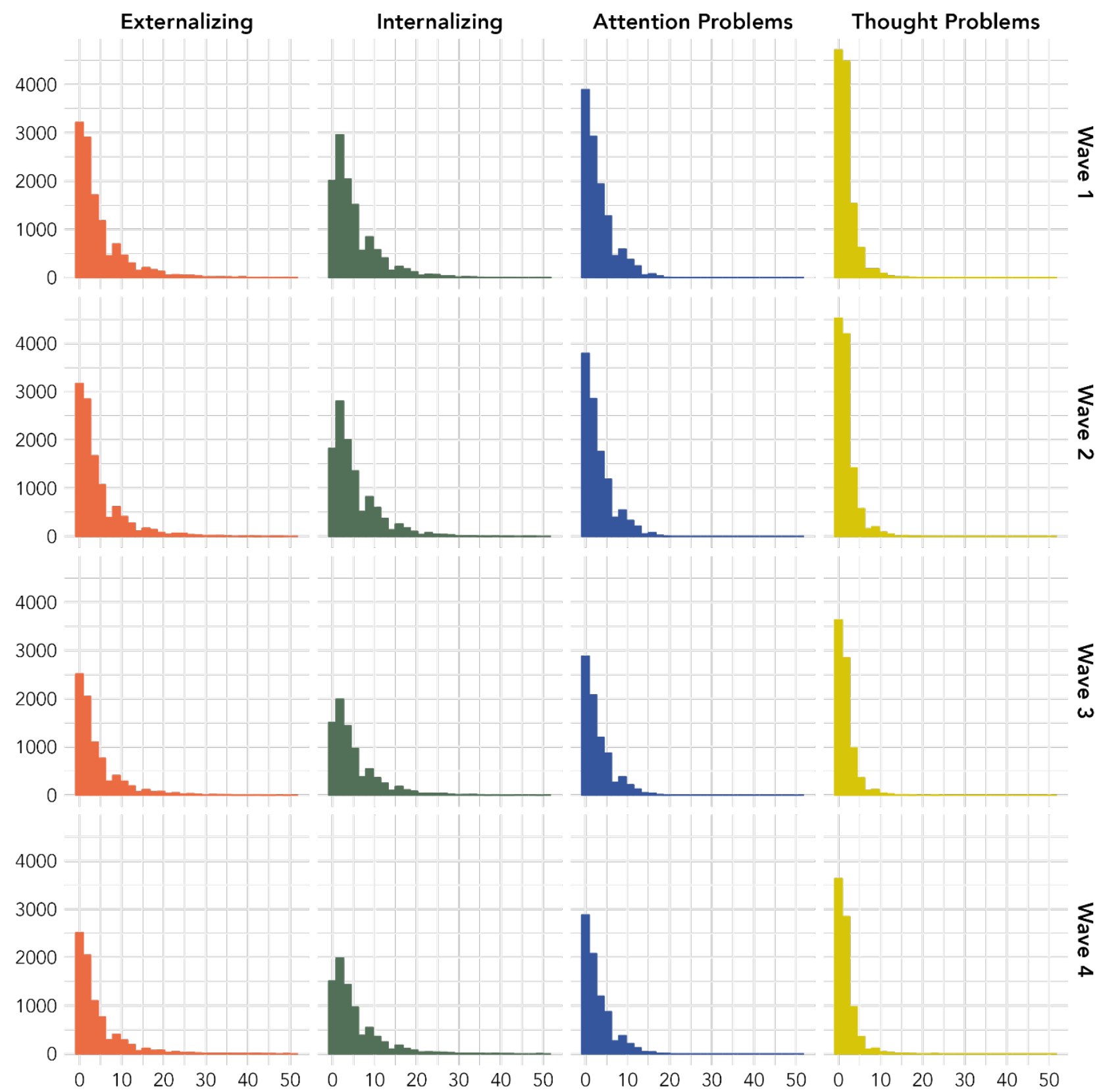

Figure A1. *Univariate distributions of higher-order psychopathology dimensions across all waves.*

Even if high severity cases in the tail have large residuals, they are typically rare, so their total contribution to the sum can be smaller than the combined contribution of the large central mass. In turn, the OLS (/Gaussian) likelihood is dominated by the highest-density region of the data such that parameter estimates are primarily driven by optimizing fit (r^2) where the sample density is highest.

Returning to the two opening statements that are seemingly contradictory, they can both be true because they are referring to different processes. The association between psychopathology dimensions estimated using OLS/Gaussian likelihood is heavily influenced by the large mass of largely asymptomatic individuals. Because most people in the sample have few (or no) symptoms, the overall correlation mostly reflects how “healthy” people cluster together, rather than how

symptoms co-occur among those who experience psychopathology symptoms. This problem can shape the estimated relationship that is interpreted as “comorbidity”.

It is, of course, true that individuals can receive multiple mental disorder diagnoses at a given time, as shown in our study where 2.2% of the youths in the ABCD Study have clinically severe levels of both externalizing and internalizing psychopathology at baseline.

(a)

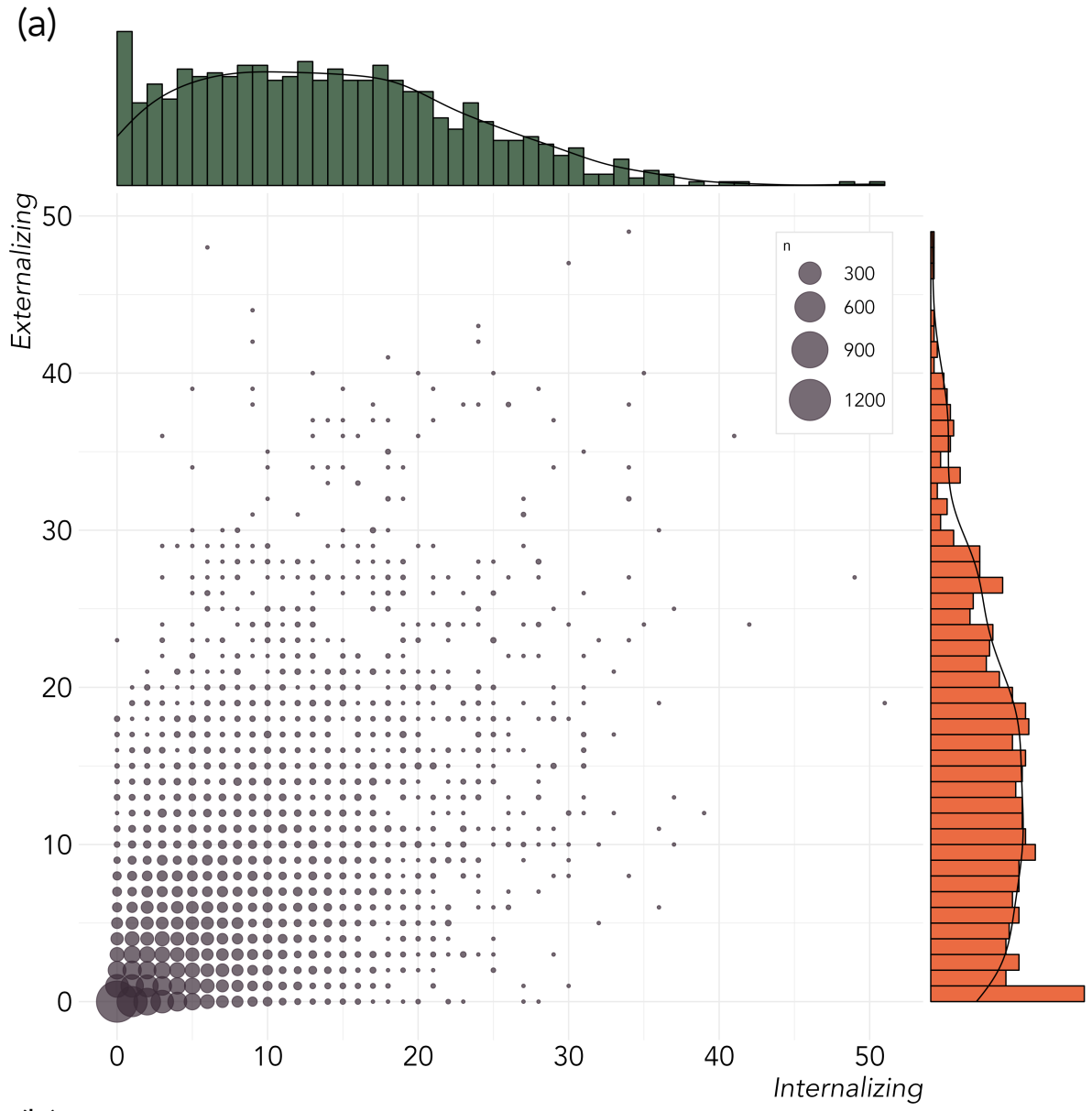

(b)

	Classification Combinations	% of sample
Nomorbidity	Nonclinical Internalizing & Externalizing	81.4
Internalizing	At-risk Internalizing	4.8
	Clinical Internalizing	3.3
Externalizing	At-risk Externalizing	3.2
	Clinical Externalizing	1.9
Comorbidity	At-risk Internalizing & At-risk Externalizing	0.9
	At-risk Internalizing & Clinical Externalizing	0.9
	At-risk Externalizing & Clinical Internalizing	1.3
	Clinical Internalizing & Externalizing	2.2

Figure A2 (also Figure 1 in the main text). Distributions of externalizing and internalizing across levels of clinical severity.

Note. Panel A depicts the bivariate distribution between externalizing and internalizing dimensions, with the marginal distributions for each on their respective axes. The size of the individual points corresponds to the number of cases at each point – the larger the point, the more cases. Panel B describes the percentage of cases that make up different categories of clinical severity for externalizing and internalizing based on the T-score categorizations for the Child Behavior Checklist. T scores below 60 correspond to nonclinical levels, between 60 and 63 correspond to ‘at-risk’ or borderline levels, and greater than 64 correspond to clinical levels. Table C1 (Appendix C) contains a translation between T scores and raw scores for all scales across all waves.

Once someone has a first diagnosis, they are no longer a random draw from the population. Instead, they are a selected subgroup with stronger psychopathology severity and/or increased clinical contact, with more opportunities for detection (ascertainment bias). With respect to the latter, we recognize that this possibility does not completely explain away population-based epidemiologic data that also find statistical instances of comorbidity and multimorbidity. Regardless, in both instances, the conditional probability of achieving a second diagnosis can rise.

But these two statements can occur simultaneously because they emerge from different levels of analysis. Our point about ‘asymptomatic’ individuals is about population-level estimation, or estimation in community samples with many asymptomatic or undiagnosed cases: Because most individuals have few symptoms, likelihood-based/OLS estimates of cross-domain associations are disproportionately influenced by the large asymptomatic group that makes up the plurality of cases in population-level data.

In contrast, the increased probability of receiving an additional diagnosis after a first diagnosis reflects selection or ascertainment bias: Once an individual meets criteria for an initial disorder they are enriched for higher underlying liability and are more likely to be under clinical observation, which increases detection of additional conditions. Thus, comorbidity parameter estimates can be driven by the low-symptom majority in the full sample, and clinically observed sequential diagnoses can still be more frequent among those already diagnosed. Even though comorbidity and multimorbidity occur in the population, they are statistically rare and thus are not as heavily weighted in the OLS regression model.

Appendix B

Appendix B Tables

Title		Page
**B1**	Demographics of ABCD Study sample across all four waves.	**3**
**B2**	Reliability of the Child Behavior Checklist Scales.	**4**
**B3**	Results from 1-changepoint negative binomial regression models for externalizing and internalizing across all waves.	**5**
**B4**	Univariate and bivariate normality for higher-order psychopathology dimensions at wave two.	**6**
**B5**	Univariate and bivariate normality for higher-order psychopathology dimensions at wave three.	**7**
**B6**	Univariate and bivariate normality for higher-order psychopathology dimensions at wave four.	**8**
**B7**	Results from 1-changepoint negative binomial regression models for externalizing and attention problems across all waves.	**9**
**B8**	Results from 1-changepoint negative binomial regression models for internalizing and attention problems across all waves.	**10**
**B9**	Results from 1-changepoint negative binomial regression models for externalizing and thought problems across all waves.	**11**
**B10**	Results from 1-changepoint negative binomial regression models for internalizing and thought problems across all waves.	**12**
**B11**	Results from 1-changepoint negative binomial regression models for attention problems and thought problems.	**13**
**B12**	Univariate and bivariate normality for CBCL higher-order psychopathology dimensions' factor scores at wave one.	**14**
**B13**	Univariate and bivariate normality for CBCL higher-order psychopathology dimensions' factor scores at wave two.	**15**
**B14**	Univariate and bivariate normality for CBCL higher-order psychopathology dimensions' factor scores at wave three.	**16**
**B15**	Univariate and bivariate normality for CBCL higher-order psychopathology dimensions' factor scores at wave four.	**17**
**B16**	Univariate and bivariate normality for Adult Self-report higher-order psychopathology dimensions at wave one.	**18**
**B17**	Univariate and bivariate normality for Adult Self-report higher-order psychopathology dimensions at wave three.	**19**

Appendix B Figures

Title		Page
**B1**	Trace-plots for 1-changepoint negative binomial regression models with externalizing and internalizing at wave one.	**20**
**B2**	Trace-plots for 1-changepoint negative binomial regression models with externalizing and internalizing at wave two.	**21**
**B3**	Trace-plots for 1-changepoint negative binomial regression models with externalizing and internalizing at wave three.	**22**
**B4**	Trace-plots for 1-changepoint negative binomial regression models with externalizing and internalizing at wave four.	**23**
**B5**	Univariate distributions of higher-order psychopathology dimensions across all waves.	**24**
**B6**	Comparison of the leave-one-out information criterion for 0-, 1-, and 2-changepoint Gaussian and negative binomial regression models across all waves.	**25**
**B7**	Comparison of the model R-squared for 0-, 1-, and 2-changepoint Gaussian and negative binomial regression models across all waves.	**26**
**B8**	Distribution of CBCL higher-order psychopathology dimensions' factor scores across all waves.	**27**
**B9**	Regression linearity assumption checks for Gaussian models that regressed externalizing onto internalizing across all waves.	**28**
**B10**	Regression linearity assumption checks for Gaussian models that regressed higher-order psychopathology dimensions onto one another at wave 1.	**29**
**B11**	Distribution of Adult Self-report higher-order psychopathology dimensions across all available waves.	**30**

Table B1.

Demographics of ABCD Study sample across all four waves.

	Wave 1	Wave 2	Wave 3	Wave 4
N	11,868	11,220	10,973	10,366
M_{age} (SD)	9.92 (.63)	10.92 (.64)	12.02 (.67)	12.91 (.65)
% female	48	48	48	48
% White	74	75	75	77
% Black	21	20	20	19
% Asian	7	7	7	7
% Other	11	11	11	10
% Hispanic	20	20	20	20

Note. Race (White, Black, Asian, Other) and Ethnicity (Hispanic) categories are not mutually exclusive; participants could endorse multiple categories.

Table B2.
Reliability of the Child Behavior Checklist Scales.

CBCL Scale	*N* items	Max score	Wave 1	Wave 2	Wave 3	Wave 4
Externalizing	35	70	0.89	0.89	0.89	0.89
Internalizing	32	64	0.87	0.87	0.88	0.88
Attention Problems	10	20	0.86	0.86	0.85	0.85
Thought Problems	15	30	0.70	0.71	0.72	0.71

Note. Max score indicates the maximum possible score on the scale, not the maximum observed in this study.

Table B3.
Univariate and bivariate normality for higher-order psychopathology dimensions at wave two.

	Univariate Normality		Bivariate Normality		
Predictor	Skewness	Kurtosis	Criterion	Skewness	Kurtosis
			Internalizing	17902	204
Externalizing	2.41	7.66	Attention Problems	15378	157
			Thought Problems	19479	222
			Externalizing	17902	204
Internalizing	1.92	5.00	Attention Problems	10746	110
			Thought Problems	15785	182
			Externalizing	15378	157
Attention Problems	1.48	1.95	Internalizing	10746	110
			Thought Problems	13075	136
			Externalizing	19479	222
Thought Problems	2.28	6.90	Internalizing	15785	182
			Attention Problems	13075	136

Note. Univariate skewness values > 1 or < - 1 indicate a high degree of skewness, and univariate kurtosis values > 2 or < -2 indicate substantial nonnormality. Higher estimates of Mardia bivariate skewness and kurtosis indicate deviations from bivariate normality, with 0 indicating true multivariate normality. Bonferroni-corrected *p* values associated with the Mardia statistics were significant at $p<.001$, indicating that the data were significantly deviant from bivariate normality.

Table B4.
Univariate and bivariate normality for higher-order psychopathology dimensions at wave three.

	Univariate Normality		Bivariate Normality		
Predictor	Skewness	Kurtosis	Criterion	Skewness	Kurtosis
			Internalizing	13940	203
Externalizing	2.52	8.55	Attention Problems	11800	152
			Thought Problems	15006	211
			Externalizing	13940	203
Internalizing	2.05	5.68	Attention Problems	8606	116
			Thought Problems	12581	195
			Externalizing	11800	152
Attention Problems	1.51	2.07	Internalizing	8606	116
			Thought Problems	10343	137
			Externalizing	15006	211
Thought Problems	2.42	8.33	Internalizing	12581	195
			Attention Problems	10343	137

Note. Univariate skewness values > 1 or < - 1 indicate a high degree of skewness, and univariate kurtosis values > 2 or < -2 indicate substantial nonnormality. Higher estimates of Mardia bivariate skewness and kurtosis indicate deviations from bivariate normality, with 0 indicating true multivariate normality. Bonferroni-corrected *p* values associated with the Mardia statistics were significant at $p<.001$, indicating that the data were significantly deviant from bivariate normality.

Table B5.
Univariate and bivariate normality for higher-order psychopathology dimensions at wave four.

	Univariate Normality		Bivariate Normality		
Predictor	Skewness	Kurtosis	Criterion	Skewness	Kurtosis
			Internalizing	12826	175
Externalizing	2.45	7.95	Attention Problems	10388	130
			Thought Problems	14022	197
			Externalizing	12826	175
Internalizing	1.95	4.96	Attention Problems	7346	89
			Thought Problems	12348	177
			Externalizing	10388	130
Attention Problems	1.46	1.91	Internalizing	7346	89
			Thought Problems	9838	130
			Externalizing	14022	197
Thought Problems	2.47	8.78	Internalizing	12348	177
			Attention Problems	9838	130

Note. Univariate skewness values > 1 or < - 1 indicate a high degree of skewness, and univariate kurtosis values > 2 or < -2 indicate substantial nonnormality. Higher estimates of Mardia bivariate skewness and kurtosis indicate deviations from bivariate normality, with 0 indicating true multivariate normality. Bonferroni-corrected *p* values associated with the Mardia statistics were significant at p<.001, indicating that the data were significantly deviant from bivariate normality.

Table B6.

Results from 1-changepoint negative binomial regression models for externalizing and internalizing across all waves.

			**Changepoint Location**			**Slopes**								**Intercepts**					
			**T_1**			**B_1**				**B_2**				**a_1**			**a_2**		
wave	LOOIC	Model R^2	T_1	SE	$\hat{R}$	B_1	SE	$\hat{R}$	% exp(B_1)	B_2	SE	$\hat{R}$	% exp(B_2)	a_1	SE	$\hat{R}$	a_2	SE	$\hat{R}$
Predictor: Internalizing, Criterion: Externalizing																			
1	60333	0.56	4.85	.07	1.04	.28	.00	1.05	32	.07	.00	1.03	7	0.78	.01	1.00	1.80	.01	1.01
2	54713	0.63	4.61	.04	1.01	.28	.00	1.02	33	.07	.00	1.01	8	0.49	.01	1.01	1.45	.01	1.01
3	37870	0.64	3.57	.02	1.00	.38	.00	1.00	46	.07	.00	1.00	8	-0.07	.01	1.00	1.01	.01	1.00
4	35727	0.60	3.53	.02	1.01	.38	.00	1.01	46	.07	.00	1.01	8	0.27	.02	1.02	1.35	.03	1.02
Predictor: Externalizing, Criterion: Internalizing																			
1	66014	0.56	2.52	.01	1.00	.36	.00	1.00	44	.06	.00	1.00	6	0.02	.00	1.00	0.81	.00	1.00
2	62378	0.62	2.51	.01	1.00	.37	.00	1.00	45	.06	.00	1.00	6	0.12	.01	1.00	0.89	.01	1.00
3	43895	0.65	2.51	.01	1.00	.39	.00	1.00	48	.05	.00	1.00	5	0.06	.01	1.00	0.89	.01	1.00
4	42130	0.64	2.87	.04	1.04	.37	.00	1.03	44	.05	.00	1.01	5	0.15	.01	1.00	1.06	.01	1.00

Note. B = unstandardized beta (slope) coefficients; exp(B_1) = exponentiated beta coefficient; $\hat{R}$= scale reduction factor (convergence criterion); SE = standard error; T = tau, or the changepoint location. The exponentiated beta coefficients reflect the percent increase in the criterion variable per point of the predictor variable.

Table B7.
Results from 1-changepoint negative binomial regression models for externalizing and attention problems across all waves.

			**Changepoint Location**			**Slopes**								**Intercepts**					
			**T_1**			**B_1**				**B_2**				**a_1**			**a_2**		
wave	LOOIC	Model R^2	T_1	SE	$\hat{R}$	B_1	SE	$\hat{R}$	% exp(B_1)	B_2	SE	$\hat{R}$	% exp(B_2)	a_1	SE	$\hat{R}$	a_2	SE	$\hat{R}$
Predictor: Attention Problems, Criterion: Externalizing																			
1	59444	0.57	1.50	.00	1.00	.76	.00	1.00	113	.15	.00	1.00	16	0.27	.00	1.00	1.08	.00	1.00
2	53920	0.58	1.50	.00	1.00	.73	.00	1.00	107	.14	.00	1.00	16	0.08	.00	1.00	0.92	.00	1.00
3	37340	0.63	1.50	.00	1.00	.78	.00	1.00	118	.16	.00	1.00	17	-0.20	.01	1.00	0.63	.01	1.00
4	35194	0.63	1.50	.00	1.00	.81	.00	1.00	124	.15	.00	1.00	17	0.12	.01	1.00	1.02	.01	1.00
Predictor: Externalizing, Criterion: Attention Problems																			
1	56754	0.47	11.16	.05	1.01	.46	.00	1.01	59	.26	.00	1.02	30	0.46	.03	1.01	2.77	.03	1.02
2	53017	0.41	12.29	.09	1.02	.47	.00	1.01	60	.25	.00	1.00	28	0.67	.02	1.00	3.26	.02	1.01
3	37477	0.47	8.99	.12	1.00	.48	.00	1.00	61	.27	.00	1.00	31	0.42	.02	1.00	2.34	.03	1.00
4	35419	0.48	6.85	.06	1.01	.54	.00	1.00	72	.30	.00	1.02	35	0.51	.04	1.01	2.13	.04	1.02

Note. B = unstandardized beta (slope) coefficients; exp(B_1) = exponentiated beta coefficient; $\hat{R}$= scale reduction factor (convergence criterion); SE = standard error; T = tau, or the changepoint location. The exponentiated beta coefficients reflect the percent increase in the criterion variable per point of the predictor variable.

Table B8.

Results from 1-changepoint negative binomial regression models for internalizing and attention problems across all waves.

			**Changepoint Location**			**Slopes**								**Intercepts**					
			**T_1**			**B_1**				**B_2**				**a_1**			**a_2**		
wave	LOOIC	Model R^2	T_1	SE	$\hat{R}$	B_1	SE	$\hat{R}$	% exp(B_1)	B_2	SE	$\hat{R}$	% exp(B_2)	a_1	SE	$\hat{R}$	a_2	SE	$\hat{R}$
Predictor: Attention Problems, Criterion: Internalizing																			
1	66748	0.51	1.07	.05	1.02	.25	.05	1.03	28	.11	.00	1.02	11	0.24	.01	1.01	0.65	.01	1.01
2	63067	0.54	1.33	.07	1.07	.40	.07	1.02	49	.10	.00	1.03	10	0.28	.01	1.00	0.75	.01	1.01
3	44323	0.60	0.68	.03	1.02	.05	.06	1.00	5	.11	.00	1.00	11	0.08	.02	1.01	0.58	.03	1.01
4	42536	0.56	1.28	.11	1.17	.47	.05	1.03	59	.11	.00	1.16	12	0.38	.03	1.05	0.87	.03	1.08
Predictor: Internalizing, Criterion: Attention Problems																			
1	51800	0.27	3.82	.05	1.02	.27	.00	1.03	30	.06	.00	1.01	6	0.51	.01	1.01	1.29	.01	1.01
2	47565	0.24	2.44	.24	1.27	.39	.05	1.57	48	.07	.00	1.18	7	0.52	.01	1.03	1.24	.01	1.04
3	33061	0.25	3.03	.05	1.01	.34	.00	1.01	40	.06	.00	1.01	6	0.06	.01	1.00	0.89	.01	1.00
4	31724	0.27	2.80	.03	1.01	.36	.00	1.01	43	.06	.00	1.00	6	0.40	.02	1.02	1.24	.02	1.02

Note. B = unstandardized beta (slope) coefficients; exp(B_1) = exponentiated beta coefficient; $\hat{R}$= scale reduction factor (convergence criterion); SE = standard error; T = tau, or the changepoint location. The exponentiated beta coefficients reflect the percent increase in the criterion variable per point of the predictor variable.

Table B9.

Results from 1-changepoint negative binomial regression models for externalizing and thought problems across all waves.

			Changepoint Location			Slopes								Intercepts					
			T_1			B_1				B_2				a_1			a_2		
wave	LOOIC	Model R^2	T_1	SE	$\hat{R}$	B_1	SE	$\hat{R}$	% exp(B_1)	B_2	SE	$\hat{R}$	% exp(B_2)	a_1	SE	$\hat{R}$	a_2	SE	$\hat{R}$
Predictor: Thought Problems, Criterion: Externalizing																			
1	60466	0.60	2.51	.01	1.00	.52	.00	1.00	68	.15	.00	1.00	16	0.85	.01	1.01	1.79	.01	1.01
2	54905	0.65	2.57	.04	1.02	.49	.00	1.03	63	.15	.00	1.02	16	0.44	.01	1.01	1.35	.01	1.00
3	38103	0.63	2.74	.04	1.03	.49	.00	1.02	63	.15	.00	1.03	17	0.05	.02	1.01	0.97	.02	1.02
4	35933	0.61	1.77	.06	1.06	.64	.01	1.08	90	.19	.00	1.02	20	0.40	.03	1.04	1.22	.03	1.03
Predictor: Externalizing, Criterion: Thought Problems																			
1	35361	0.64	5.15	.09	1.07	.24	.00	1.06	27	.06	.00	1.04	6	-0.91	.01	1.02	0.00	.01	1.02
2	33202	0.64	4.46	.05	1.01	.27	.00	1.01	31	.06	.00	1.00	6	-0.45	.01	1.01	0.45	.01	1.01
3	22369	0.55	2.41	.04	1.06	.44	.01	1.07	55	.07	.00	1.02	7	-0.61	.01	1.01	0.23	.01	1.01
4	20398	0.67	3.39	.02	1.00	.36	.00	1.00	44	.07	.00	1.00	7	-0.54	.01	1.00	0.42	.01	1.00

Note. B = unstandardized beta (slope) coefficients; exp(B_1) = exponentiated beta coefficient; $\hat{R}$= scale reduction factor (convergence criterion); SE = standard error; T = tau, or the changepoint location. The exponentiated beta coefficients reflect the percent increase in the criterion variable per point of the predictor variable.

Table B10.

Results from 1-changepoint negative binomial regression models for internalizing and thought problems across all waves.

			Changepoint Location			Slopes								Intercepts					
			T_1			B_1				B_2				a_1			a_2		
wave	LOOIC	Model R^2	T_1	SE	$\hat{R}$	B_1	SE	$\hat{R}$	% exp(B_1)	B_2	SE	$\hat{R}$	% exp(B_2)	a_1	SE	$\hat{R}$	a_2	SE	$\hat{R}$
Predictor: Thought Problems, Criterion: Internalizing																			
1	65862	0.61	2.50	.00	1.00	.47	.00	1.00	59	.13	.00	1.00	14	0.39	.00	1.00	1.20	.00	1.00
2	62040	0.65	2.50	.01	1.00	.48	.00	1.00	61	.13	.00	1.00	13	0.26	.01	1.01	1.08	.01	1.01
3	43665	0.69	2.54	.02	1.00	.47	.00	1.00	59	.13	.00	1.00	14	0.10	.01	1.01	0.97	.01	1.01
4	41840	0.65	1.57	.08	1.10	.67	.01	1.14	96	.15	.00	1.11	17	0.22	.00	1.00	1.05	.01	1.01
Predictor: Internalizing, Criterion: Thought Problems																			
1	34969	0.54	5.26	.06	1.04	.27	.00	1.03	31	.07	.00	1.03	7	-0.64	.01	1.00	0.42	.01	1.00
2	32598	0.60	5.67	.07	1.05	.26	.00	1.06	29	.07	.00	1.04	7	-0.33	.01	1.01	0.72	.01	1.01
3	21872	0.61	6.45	.05	1.02	.25	.00	1.02	28	.07	.00	1.01	7	-0.40	.01	1.01	0.75	.01	1.01
4	19937	0.67	7.06	.07	1.03	.24	.00	1.05	27	.06	.00	1.02	6	-0.39	.02	1.03	0.87	.02	1.03

Note. B = unstandardized beta (slope) coefficients; exp(B_1) = exponentiated beta coefficient; $\hat{R}$= scale reduction factor (convergence criterion); SE = standard error; T = tau, or the changepoint location. The exponentiated beta coefficients reflect the percent increase in the criterion variable per point of the predictor variable.

Table B11.

Results from 1-changepoint negative binomial regression models for attention problems and thought problems.

			Changepoint Location			Slopes								Intercepts					
			T_1			B_1				B_2				a_1			a_2		
wave	LOOIC	Model R^2	T_1	SE	$\hat{R}$	B_1	SE	$\hat{R}$	% $\exp(B_1)$	B_2	SE	$\hat{R}$	% $\exp(B_2)$	a_1	SE	$\hat{R}$	a_2	SE	$\hat{R}$
Predictor: Thought Problems, Criterion: Attention Problems																			
1	50998	0.43	2.51	.01	1.00	.52	.00	1.01	68	.12	.00	1.01	13	0.51	.01	1.00	1.50	.01	1.00
2	46720	0.44	2.49	.01	1.00	.53	.00	1.00	71	.13	.00	1.00	13	0.40	.00	1.00	1.37	.00	1.00
3	32690	0.45	2.59	.04	1.03	.50	.00	1.01	64	.13	.00	1.03	13	0.17	.01	1.00	1.14	.01	1.00
4	31336	0.43	1.72	.06	1.03	.66	.01	1.02	93	.14	.00	1.02	15	0.33	.01	1.00	1.25	.01	1.00
Predictor: Attention Problems, Criterion: Thought Problems																			
1	34889	0.61	1.51	.01	1.00	.54	.00	1.00	72	.15	.00	1.00	16	-0.90	.00	1.00	-0.33	.00	1.00
2	32610	0.69	1.51	.00	1.00	.59	.00	1.00	80	.15	.00	1.00	16	-0.55	.01	1.00	0.05	.01	1.00
3	22063	0.61	1.43	.02	1.01	.56	.01	1.02	75	.16	.00	1.00	17	-0.59	.01	1.00	-0.03	.01	1.00
4	20109	0.71	1.54	.01	1.00	.59	.00	1.00	81	.15	.00	1.00	17	-0.60	.00	1.00	0.08	.00	1.00

Note. B = unstandardized beta (slope) coefficients; $\exp(B_1)$ = exponentiated beta coefficient; $\hat{R}$= scale reduction factor (convergence criterion); SE = standard error; T = tau, or the changepoint location. The exponentiated beta coefficients reflect the percent increase in the criterion variable per point of the predictor variable.

Table B12.
Univariate and bivariate normality for CBCL higher-order psychopathology dimensions' factor scores at wave one.

	Univariate Normality		Bivariate Normality		
Predictor	Skewness	Kurtosis	Criterion	Skewness	Kurtosis
Externalizing	2.72	10.15	Internalizing	24748	278
			Attention Problems	19416	193
			Thought Problems	30162	343
Internalizing	2.15	6.32	Externalizing	24748	278
			Attention Problems	12617	128
			Thought Problems	26366	302
Attention Problems	1.34	1.33	Externalizing	19416	193
			Internalizing	12617	128
			Thought Problems	21239	208
Thought Problems	2.94	11.76	Externalizing	30162	343
			Internalizing	26366	302
			Attention Problems	21239	208

Note. CBCL = Child Behavior Checklist. Factor scores were derived by extracting a 1-factor solution and factor scores were extracted using the Thurstone regression weights.
Univariate skewness values > 1 or < - 1 indicate a high degree of skewness, and univariate kurtosis values > 2 or < -2 indicate substantial nonnormality. Higher estimates of Mardia bivariate skewness and kurtosis indicate deviations from bivariate normality, with 0 indicating true multivariate normality. Bonferroni-corrected *p* values associated with the Mardia statistics were significant at $p<.001$, indicating that the data were significantly deviant from bivariate normality.

Table B13.
Univariate and bivariate normality for CBCL higher-order psychopathology dimensions' factor scores at wave two.

	Univariate Normality		Bivariate Normality		
Predictor	Skewness	Kurtosis	Criterion	Skewness	Kurtosis
			Internalizing	24314	274
Externalizing	2.83	10.94	Attention Problems	19865	199
			Thought Problems	30520	347
			Externalizing	24314	274
Internalizing	2.12	6.00	Attention Problems	11922	120
			Thought Problems	24865	278
			Externalizing	19865	199
Attention Problems	1.38	1.45	Internalizing	11922	120
			Thought Problems	20332	201
			Externalizing	30520	347
Thought Problems	2.96	11.44	Internalizing	24865	278
			Attention Problems	20332	201

Note. CBCL = Child Behavior Checklist. Factor scores were derived by extracting a 1-factor solution and factor scores were extracted using the Thurstone regression weights.
Univariate skewness values > 1 or < - 1 indicate a high degree of skewness, and univariate kurtosis values > 2 or < -2 indicate substantial nonnormality. Higher estimates of Mardia bivariate skewness and kurtosis indicate deviations from bivariate normality, with 0 indicating true multivariate normality. Bonferroni-corrected *p* values associated with the Mardia statistics were significant at $p<.001$, indicating that the data were significantly deviant from bivariate normality.

Table B14.
Univariate and bivariate normality for CBCL higher-order psychopathology dimensions' factor scores at wave three.

	Univariate Normality		Bivariate Normality		
Predictor	Skewness	Kurtosis	Criterion	Skewness	Kurtosis
			Internalizing	26273	305
Externalizing	3.03	12.65	Attention Problems	21650	233
			Thought Problems	34848	426
			Externalizing	26273	305
Internalizing	2.24	6.71	Attention Problems	12850	129
			Thought Problems	29572	357
			Externalizing	21650	233
Attention Problems	1.41	1.57	Internalizing	12850	129
			Thought Problems	25186	291
			Externalizing	34848	426
Thought Problems	3.33	16.04	Internalizing	29572	357
			Attention Problems	25186	291

Note. CBCL = Child Behavior Checklist. Factor scores were derived by extracting a 1-factor solution and factor scores were extracted using the Thurstone regression weights.
Univariate skewness values > 1 or < - 1 indicate a high degree of skewness, and univariate kurtosis values > 2 or < -2 indicate substantial nonnormality. Higher estimates of Mardia bivariate skewness and kurtosis indicate deviations from bivariate normality, with 0 indicating true multivariate normality. Bonferroni-corrected *p* values associated with the Mardia statistics were significant at $p<.001$, indicating that the data were significantly deviant from bivariate normality.

Table B15.
Univariate and bivariate normality for CBCL higher-order psychopathology dimensions' factor scores at wave four.

	Univariate Normality		Bivariate Normality		
Predictor	Skewness	Kurtosis	Criterion	Skewness	Kurtosis
			Internalizing	24673	293
Externalizing	3.00	12.56	Attention Problems	19593	215
			Thought Problems	33253	433
			Externalizing	24673	293
Internalizing	2.19	6.32	Attention Problems	11239	116
			Thought Problems	32244	432
			Externalizing	19593	215
Attention Problems	1.37	1.42	Internalizing	11239	116
			Thought Problems	25525	302
			Externalizing	33253	433
Thought Problems	3.52	18.98	Internalizing	32244	432
			Attention Problems	25525	302

Note. CBCL = Child Behavior Checklist. Factor scores were derived by extracting a 1-factor solution and factor scores were extracted using the Thurstone regression weights.
Univariate skewness values > 1 or < - 1 indicate a high degree of skewness, and univariate kurtosis values > 2 or < -2 indicate substantial nonnormality. Higher estimates of Mardia bivariate skewness and kurtosis indicate deviations from bivariate normality, with 0 indicating true multivariate normality. Bonferroni-corrected p values associated with the Mardia statistics were significant at p<.001, indicating that the data were significantly deviant from bivariate normality.

Table B16.
Univariate and bivariate normality for Adult Self-report higher-order psychopathology dimensions at wave one.

	Univariate Normality		Bivariate Normality		
Predictor	Skewness	Kurtosis	Criterion	Skewness	Kurtosis
Externalizing	1.72	3.83	Internalizing	10408	115
			Attention Problems	8907	94
			Thought Problems	13934	160
Internalizing	1.68	3.73	Externalizing	10408	115
			Attention Problems	8762	95
			Thought Problems	13459	153
Attention Problems	1.29	1.81	Externalizing	8907	94
			Internalizing	8762	95
			Thought Problems	12510	134
Thought Problems	2.17	6.82	Externalizing	13934	160
			Internalizing	13459	153
			Attention Problems	12510	134

Note. Adult Self-report data come from primary caregivers who participated in the ABCD Study with their children. Univariate skewness values > 1 or < - 1 indicate a high degree of skewness, and univariate kurtosis values > 2 or < -2 indicate substantial nonnormality. Higher estimates of Mardia bivariate skewness and kurtosis indicate deviations from bivariate normality, with 0 indicating true multivariate normality. Bonferroni-corrected *p* values associated with the Mardia statistics were significant at $p<.001$, indicating that the data were significantly deviant from bivariate normality.

Table B17.
Univariate and bivariate normality for Adult Self-report higher-order psychopathology dimensions at wave three.

	Univariate Normality		Bivariate Normality		
Predictor	Skewness	Kurtosis	Criterion	Skewness	Kurtosis
			Internalizing	11389	150
Externalizing	1.93	6.27	Attention Problems	9678	126
			Thought Problems	15037	197
			Externalizing	11389	150
Internalizing	1.64	3.39	Attention Problems	7587	84
			Thought Problems	13316	158
			Externalizing	9678	126
Attention Problems	1.30	1.96	Internalizing	7587	84
			Thought Problems	12700	151
			Externalizing	15037	197
Thought Problems	2.29	7.76	Internalizing	13316	158
			Attention Problems	12700	151

Note. Adult Self-report data come from primary caregivers who participated in the ABCD Study with their children. Univariate skewness values > 1 or < - 1 indicate a high degree of skewness, and univariate kurtosis values > 2 or < -2 indicate substantial nonnormality. Higher estimates of Mardia bivariate skewness and kurtosis indicate deviations from bivariate normality, with 0 indicating true multivariate normality. Bonferroni-corrected p values associated with the Mardia statistics were significant at $p<.001$, indicating that the data were significantly deviant from bivariate normality.

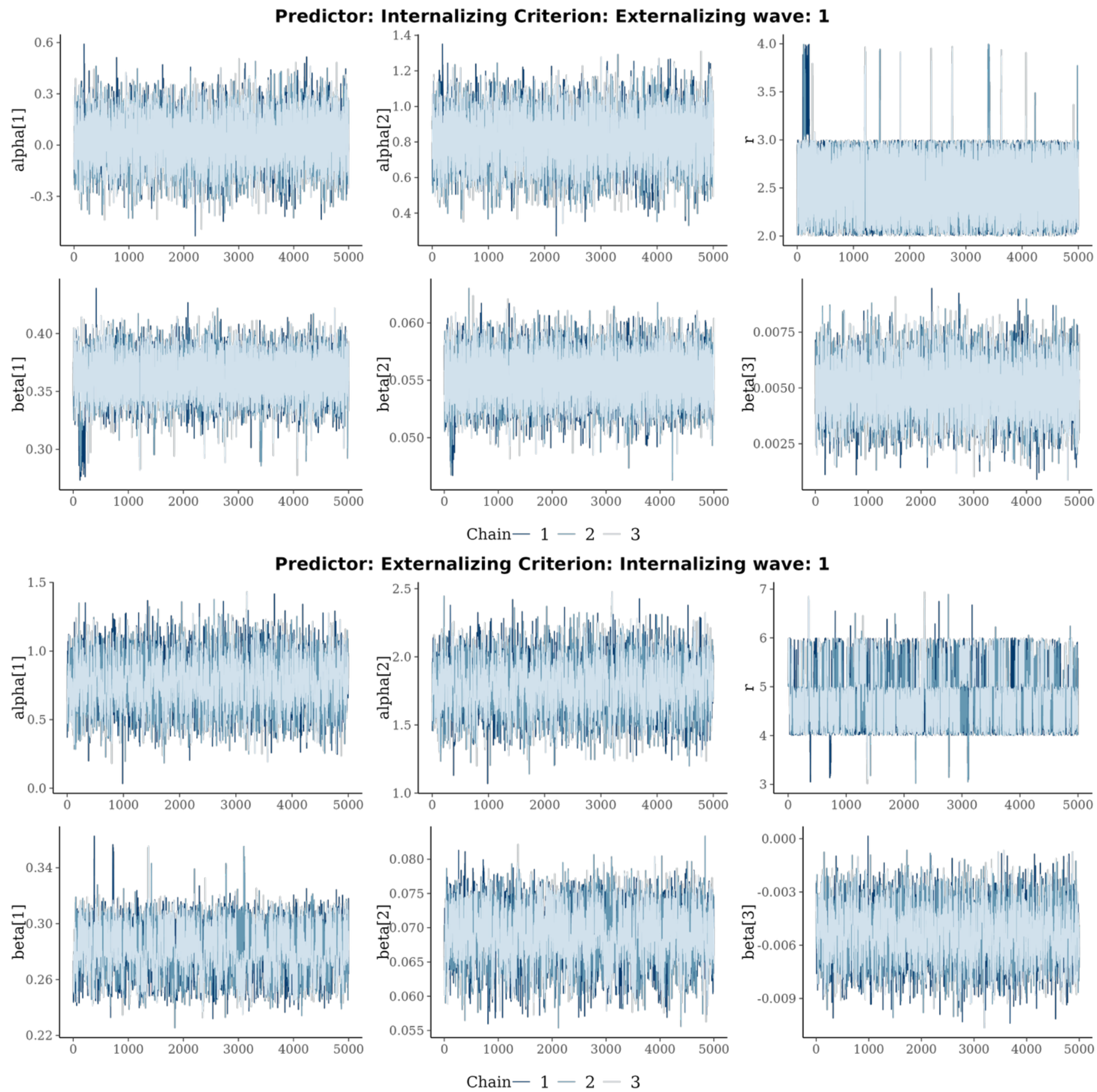

Figure B1. *Trace-plots for 1-changepoint negative binomial regression models with externalizing and internalizing at wave one.*

Note. alpha[1] = intercept of pre-changepoint regression, alpha[2] = intercept of post-changepoint regression, r = changepoint location, beta[1] = slope of pre-changepoint regression, beta[2] = slope of post-changepoint regression, beta[3] = slope of age.

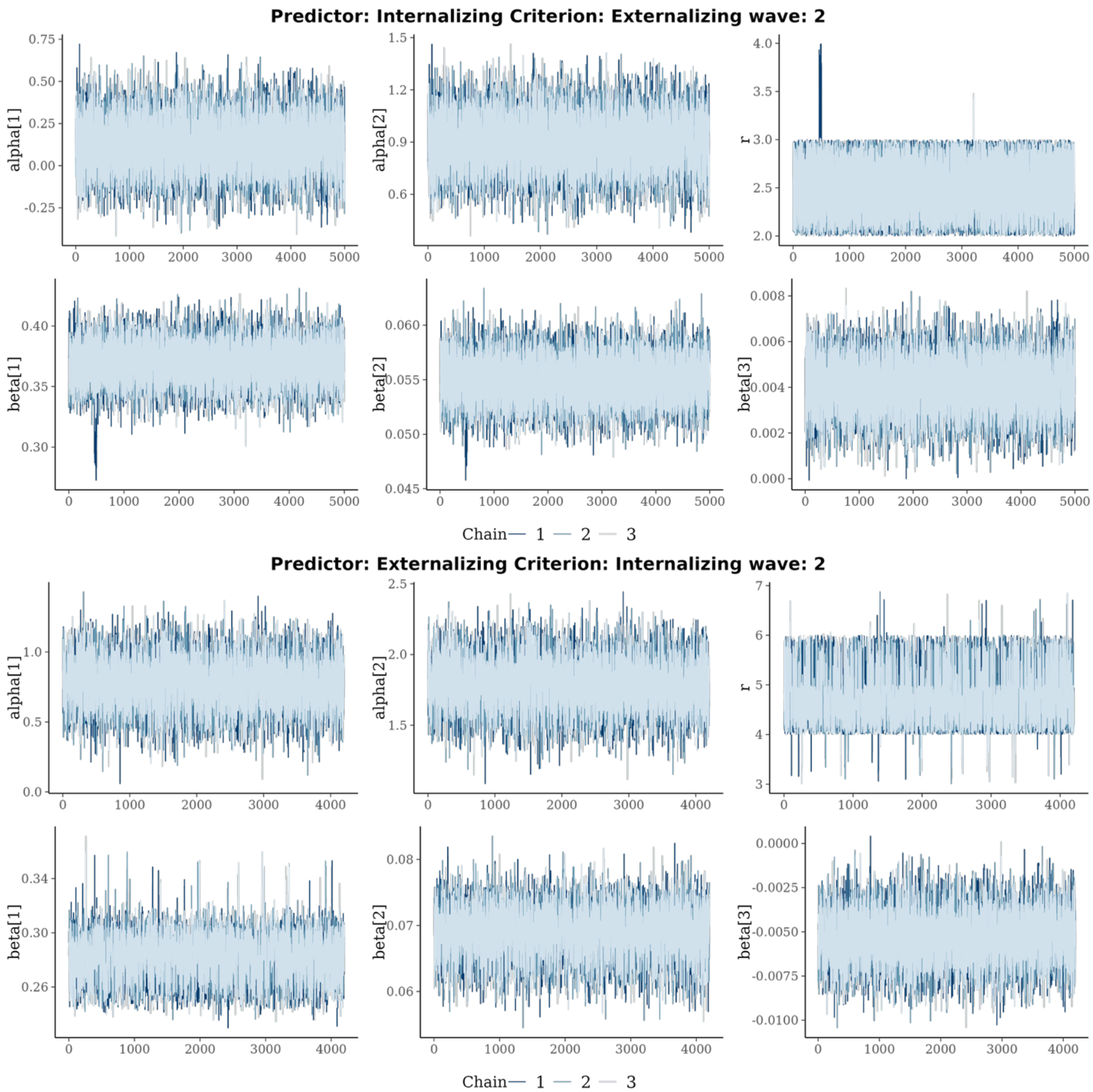

Figure B2. *Trace-plots for 1-changepoint negative binomial regression models with externalizing and internalizing at wave two.*

Note. alpha[1] = intercept of pre-changepoint regression, alpha[2] = intercept of post changepoint regression, r = changepoint location, beta[1] = slope of pre-changepoint regression, beta[2] = slope of post-changepoint regression, beta[3] = slope of age.

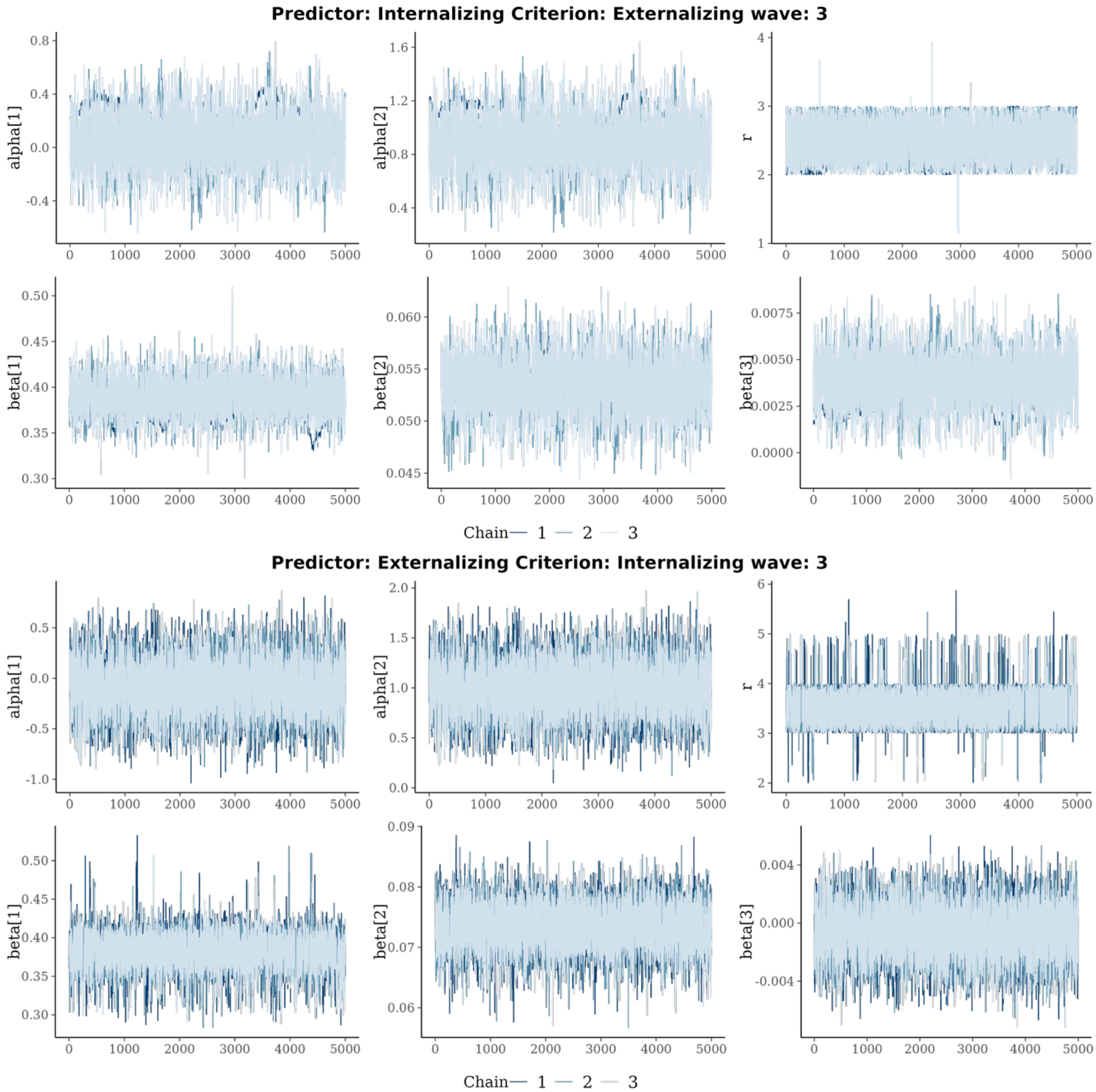

Figure B3. *Trace-plots for 1-changepoint negative binomial regression models with externalizing and internalizing at wave three.*

Note. alpha[1] = intercept of pre-changepoint regression, alpha[2] = intercept of post-changepoint regression, r = changepoint location, beta[1] = slope of pre-changepoint regression, beta[2] = slope of post-changepoint regression, beta[3] = slope of age.

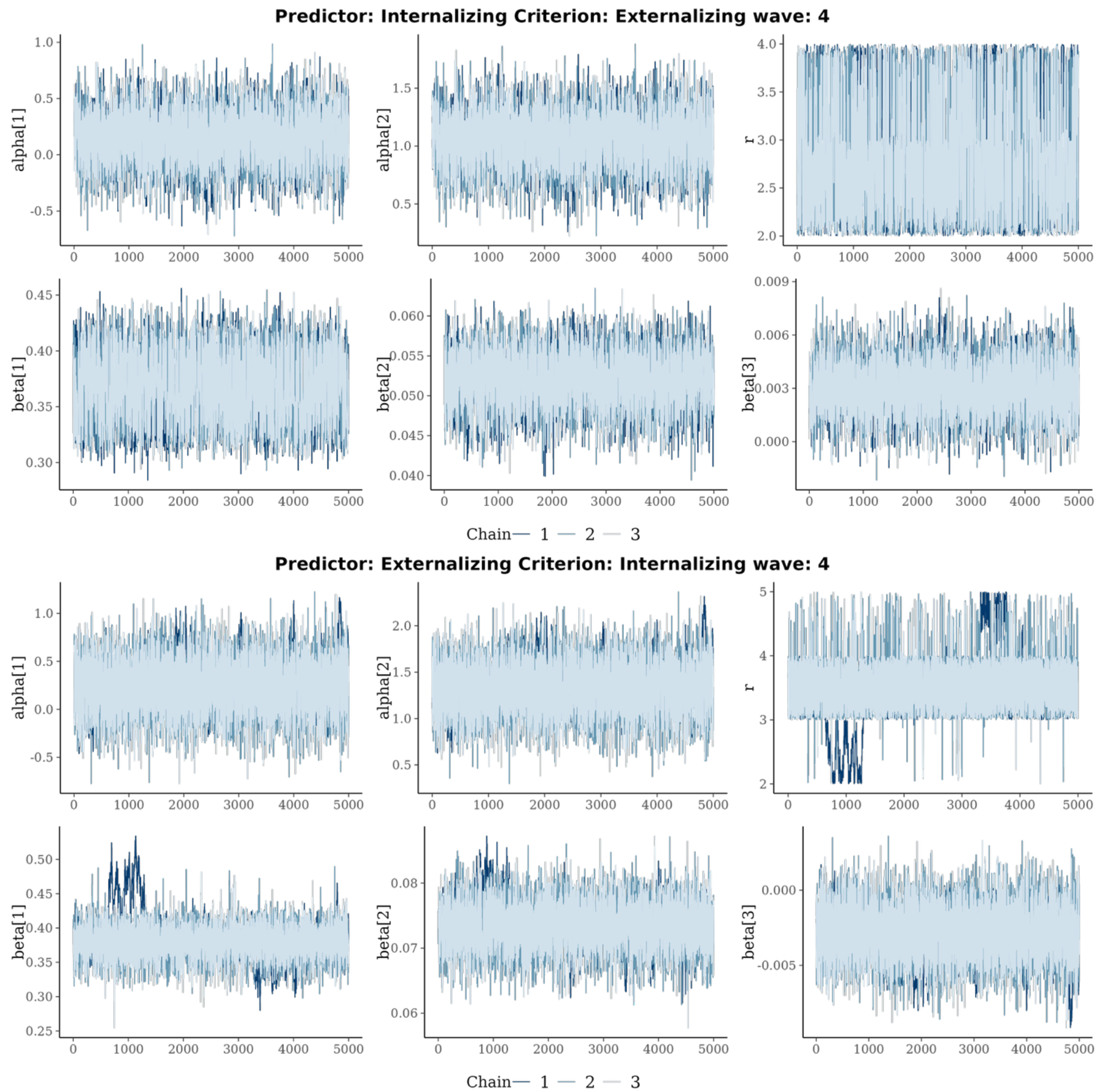

Figure B4. *Trace-plots for 1-changepoint negative binomial regression models with externalizing and internalizing at wave four.*

Note. alpha[1] = intercept of pre-changepoint regression, alpha[2] = intercept of post-changepoint regression, r = changepoint location, beta[1] = slope of pre-changepoint regression, beta[2] = slope of post-changepoint regression, beta[3] = slope of age.

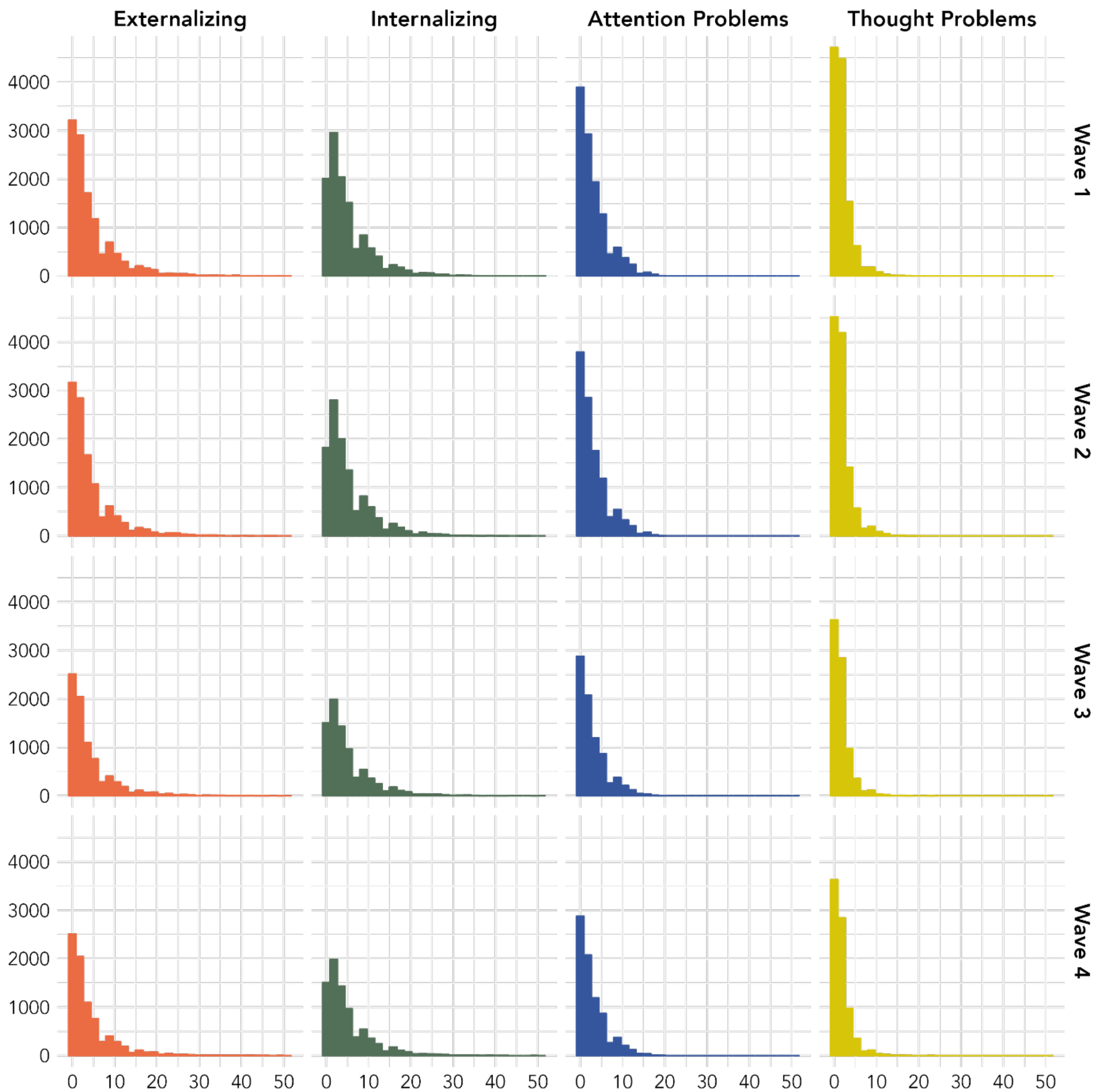

Figure B5. *Univariate distributions of higher-order psychopathology dimensions across all waves.*

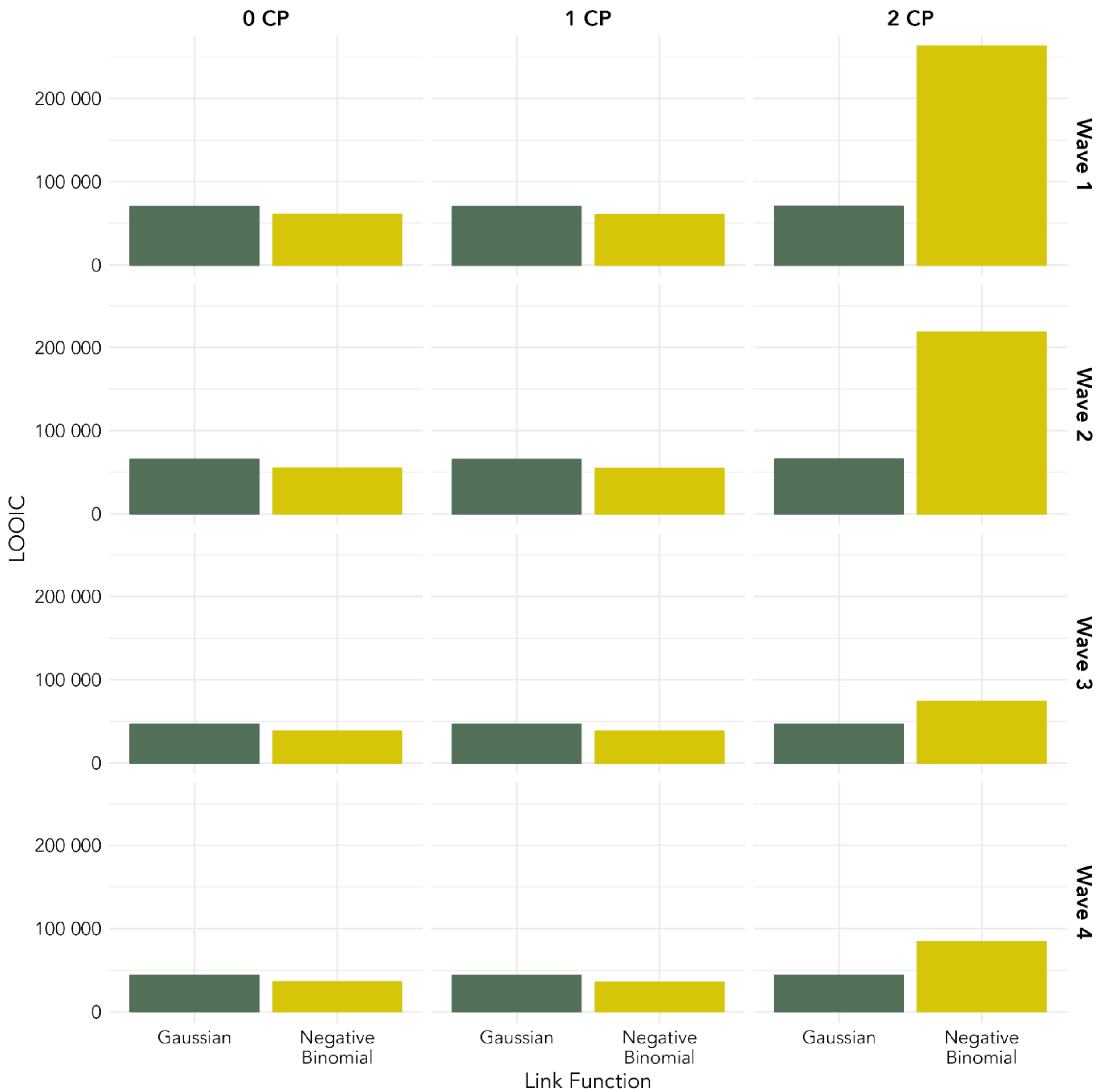

Figure B6. *Comparison of the leave-one-out information criterion for 0-, 1-, and 2-changepoint Gaussian and negative binomial regression models across all waves.*

Note. LOOIC = leave-one-out information criterion; 0CP = 0-changepoint model; 1CP = 1-changepoint model; 2CP = 2-changepoint model.

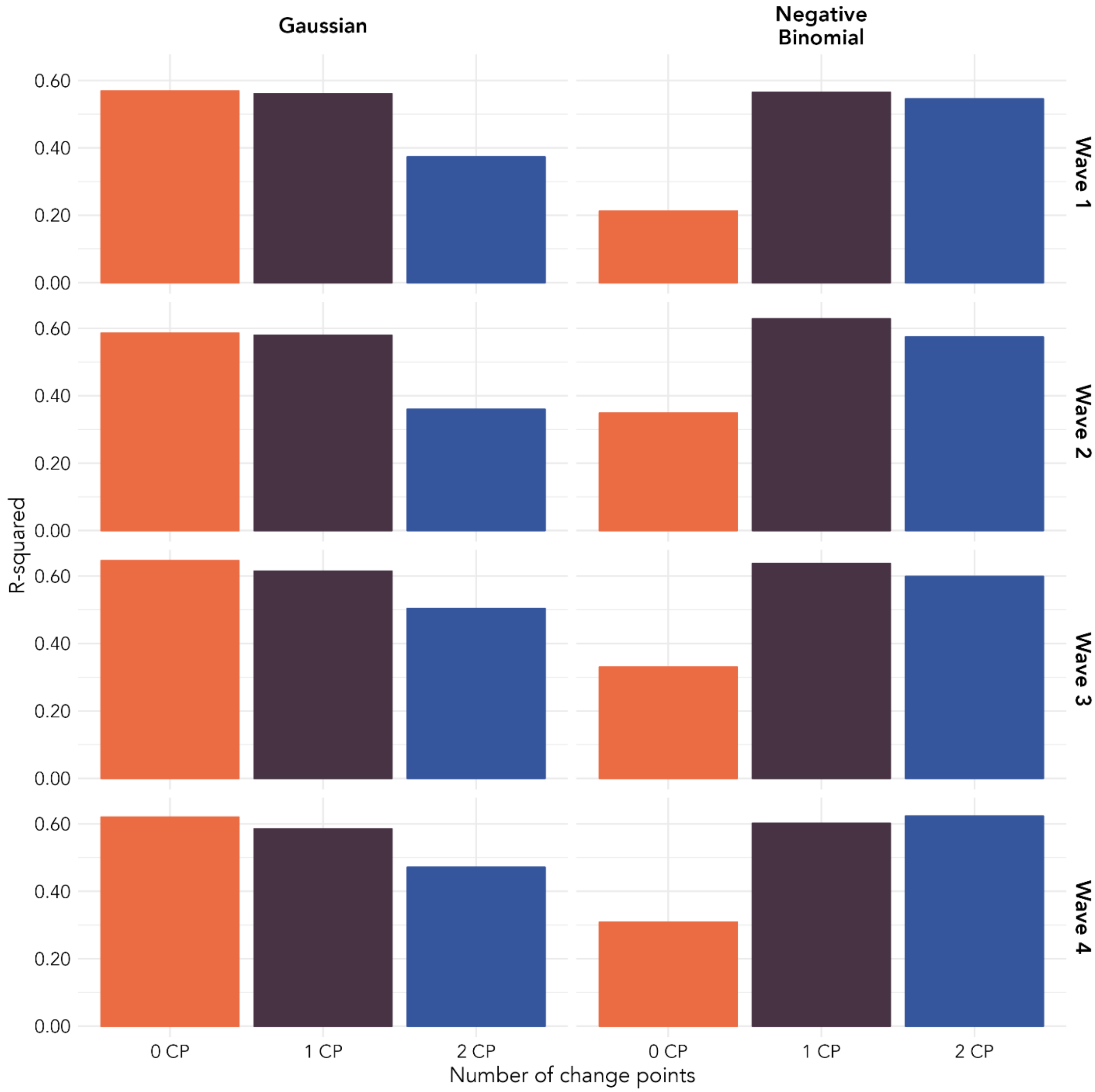

Figure B7. *Comparison of the model R-squared for 0-, 1-, and 2-changepoint Gaussian and negative binomial regression models across all waves.*

Note. 0 CP = 0-changepoint model; 1 CP = 1-changepoint model; 2 CP = 2-changepoint model.

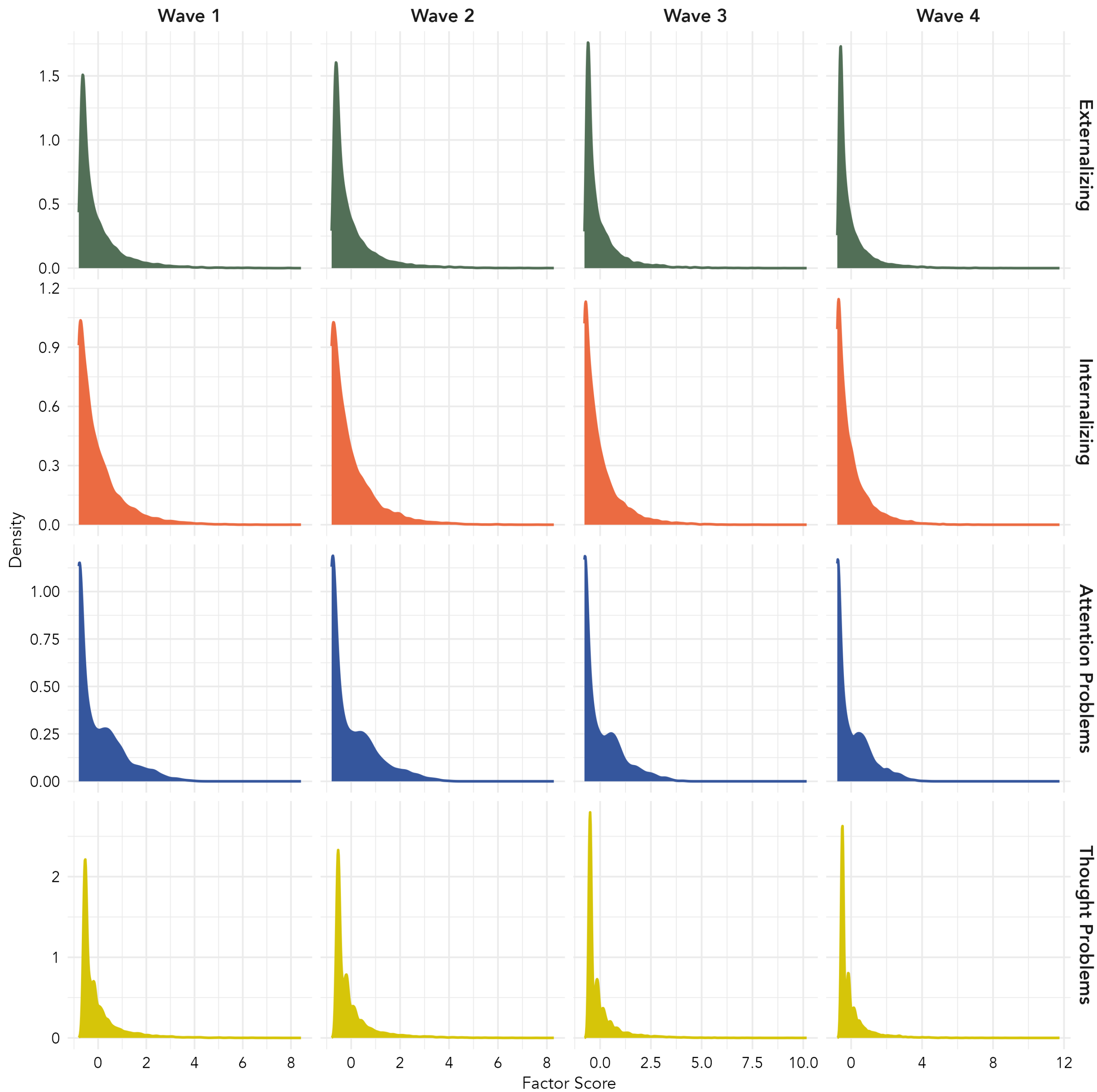

Figure B8. *Distribution of CBCL higher-order psychopathology dimensions' factor scores across all waves.*

Note. Factor scores were derived by extracting a 1-factor solution and factor scores were extracted using the Thurstone regression weights.

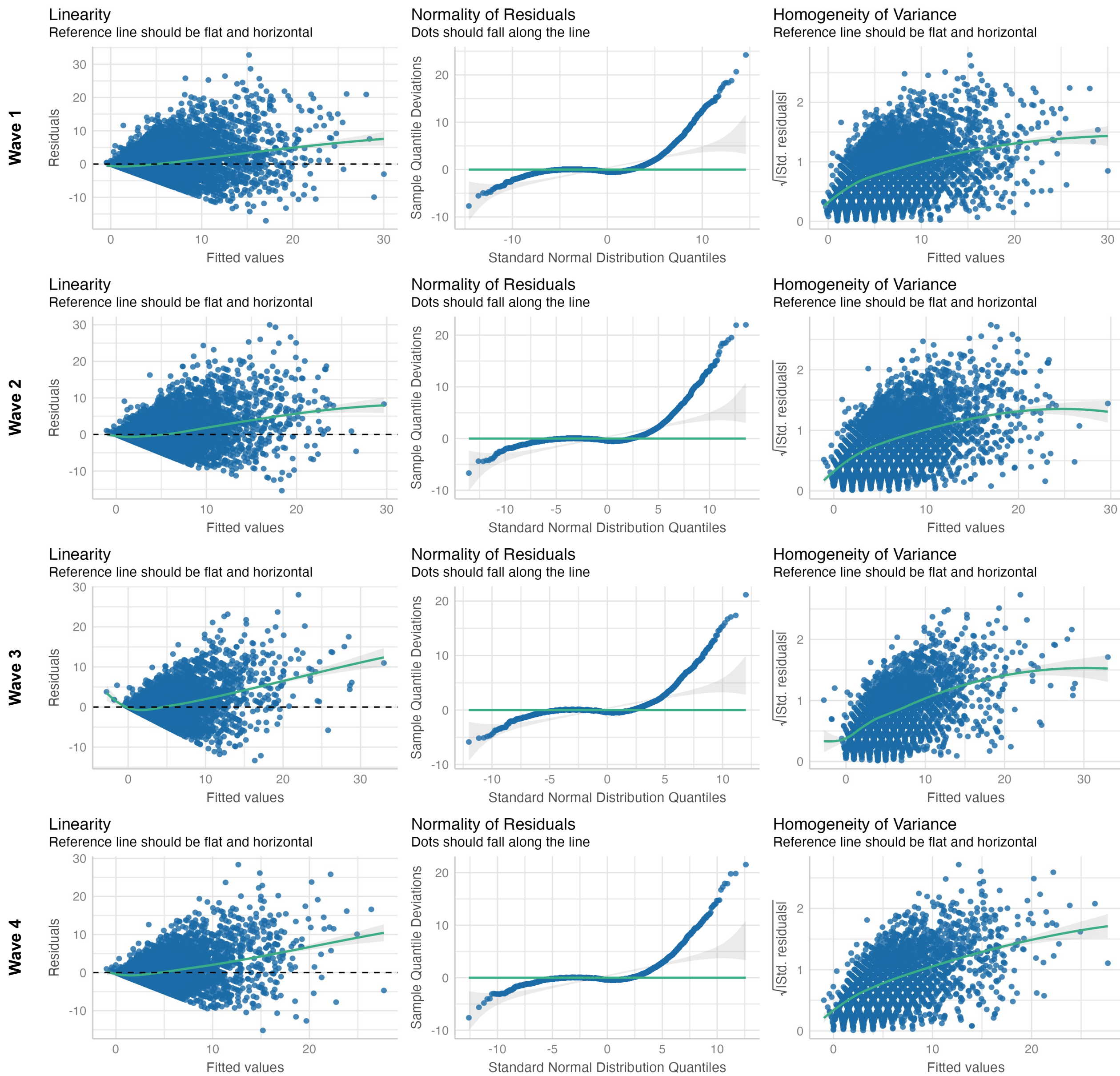

Figure B9. *Regression linearity assumption checks for Gaussian models that regressed externalizing onto internalizing across all waves.*

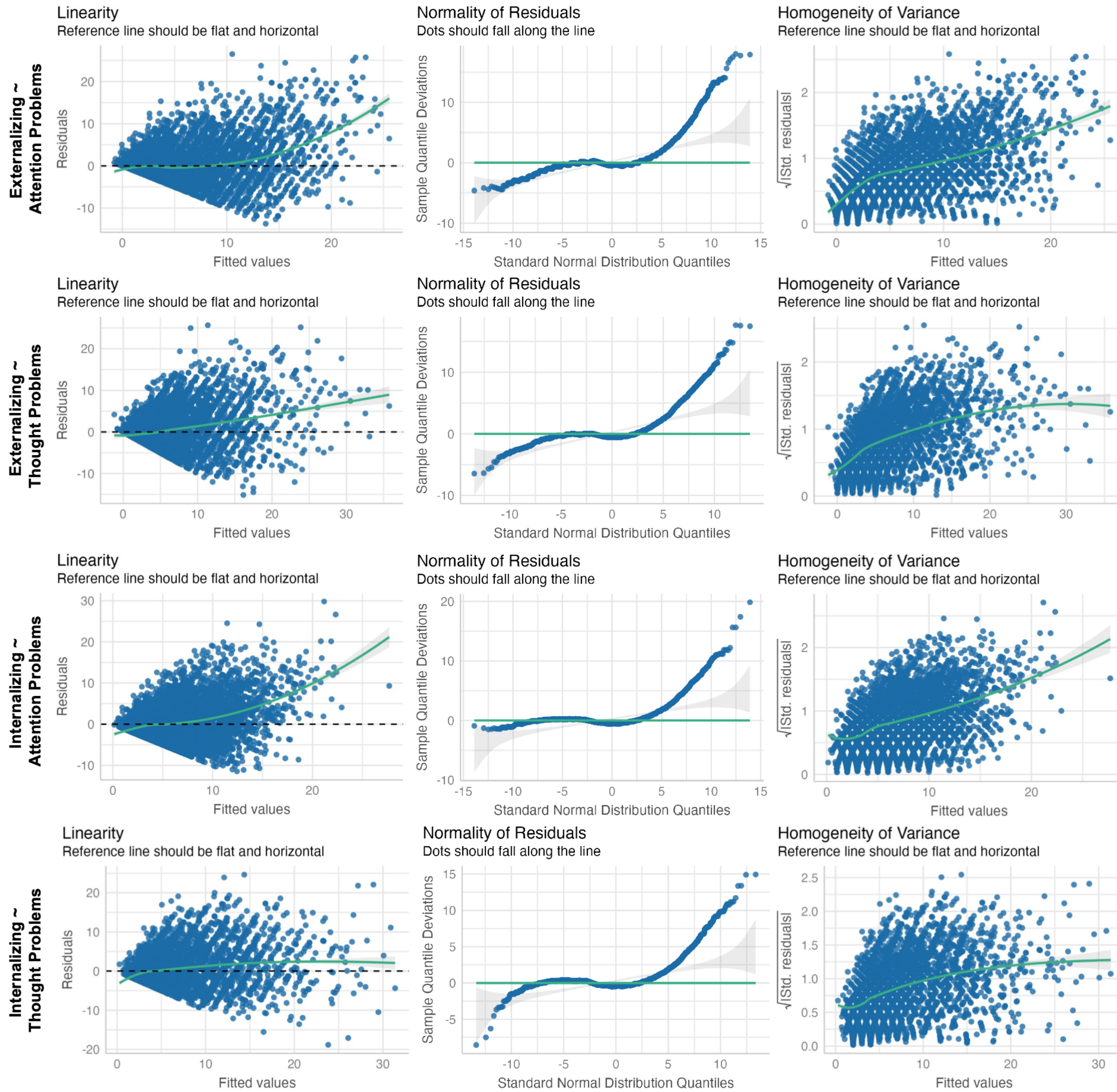

Figure B10. *Regression linearity assumption checks for Gaussian models that regressed higher-order psychopathology dimensions onto one another at wave 1.*

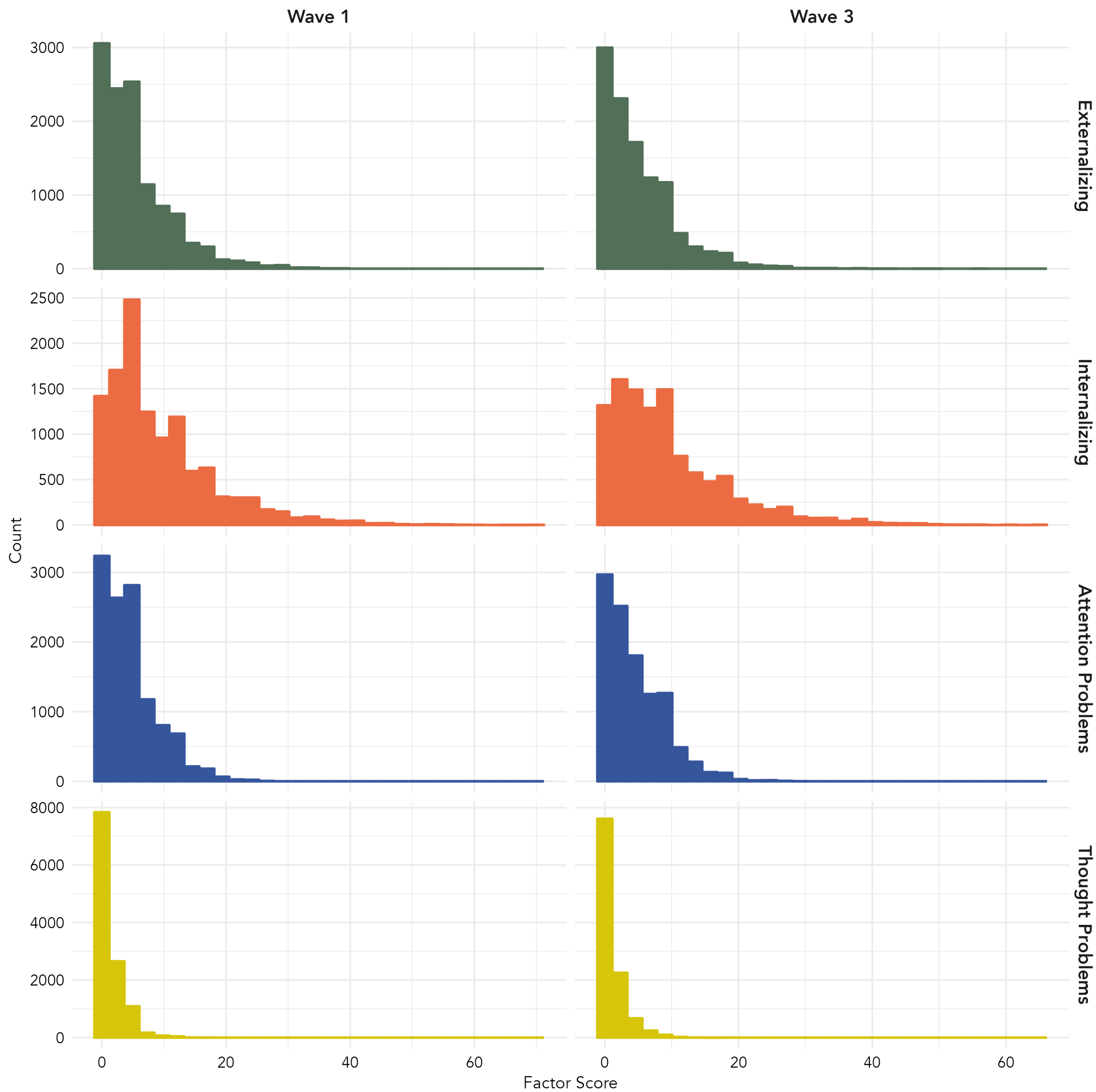

Figure B11. *Distribution of Adult Self-report higher-order psychopathology dimensions across all available waves.*

Note. Adult Self-report data come from primary caregivers who participated in the ABCD Study with their children. These data were available at waves 1 and 3.

APPENDIX D

A simulation study to illustrate how the OLS is biased when there is a nonlinear relationship between two variables

We conducted a simulation study to illustrate how the association between a predictor, x, and criterion, y, behaves when the true data-generating process follows a 1 change-point model.

Simulation summary: Here, x was drawn from a standard normal distribution and y was generated with the intercept fixed at 0, but with different slopes and error variance depending on whether x falls below or above a change point, τ. Specifically, observations with x < τ follow slope β_1 (pre-change point) and observations with x ≥ τ follow slope β_2 (post-change point). To keep the scale of y comparable across slope values, we adjusted the error within each segment to approximately preserve unit variance (via $1 - \beta^2$), ensuring correlations remain interpretable across conditions. To demonstrate bias in the ordinary least squares (OLS) model assuming no change point, we computed the Pearson correlation between x and y.

Simulation conditions: The simulation varied β_1 and β_2 over a grid from –0.8 to 0.8 (in increments of 0.2) and varied the τ over three values: –1.5, 0, 1.5. For every (β_1, β_2, τ) combination, we generated a dataset of size n=10,000.

Simulation technicalities: The data-generating model assumed:

(1) A standard normal predictor, $x_i \sim N(0,1)$, whose values were sampled i.i.d. We chose a standard normal distribution for convenience because it is easy to characterize the extent of data on each side of the change point (i.e., when τ = 0, 50% of data are on either side of the change point; τ = –1.5 yields only about 6.7% of observations in the pre segment and 93.3% in the post segment). That is, τ values correspond to Z scores.
(2) A fixed change point τ that partitions the data into a “pre” region ($x_i < \tau$) and a “post” region ($x_i \geq \tau$), implying segment membership probabilities $P(x < \tau) = \Phi(\tau)$ and $P(x \geq \tau) = 1 - \Phi(\tau)$ (i.e., truncated-normal x distributions within segments).
(3) A criterion variable that was generated from a piecewise linear model with intercept fixed at 0 and segment-specific slopes β_1 and β_2:

$$y_i = \begin{cases} \beta_1 x_i + \varepsilon_i, \text{if } x_i < \tau, \\ \beta_2 x_i + \varepsilon_i, \text{if } x_i \geq \tau. \end{cases}$$

Errors were independent Gaussian with mean 0 and segment-specific variance:

$$\varepsilon_i \mid (x_i < \tau) \sim N(0, 1 - \beta_1^2), \varepsilon_i \mid (x_i \geq \tau) \sim N(0, 1 - \beta_2^2).$$

This choice approximately standardizes the scale of y across slope magnitudes, though the overall variance of y is not guaranteed to be exactly 1 under the mixture/truncation induced by τ, so that differences in the resulting Pearson correlation primarily reflect the slope structure and the change-point location rather than trivial variance inflation. The resulting joint distribution (x, y) is therefore a two-regime linear-Gaussian mixture with heteroscedasticity (as in the data used in this study, see Figure D1 below) across regimes; moreover, the conditional mean is generally discontinuous at $x = \tau$ unless $\tau = 0$ or $\beta_1 = \beta_2$. For each (τ) condition, we computed and stored the marginal Pearson correlation from the simulated dataset.

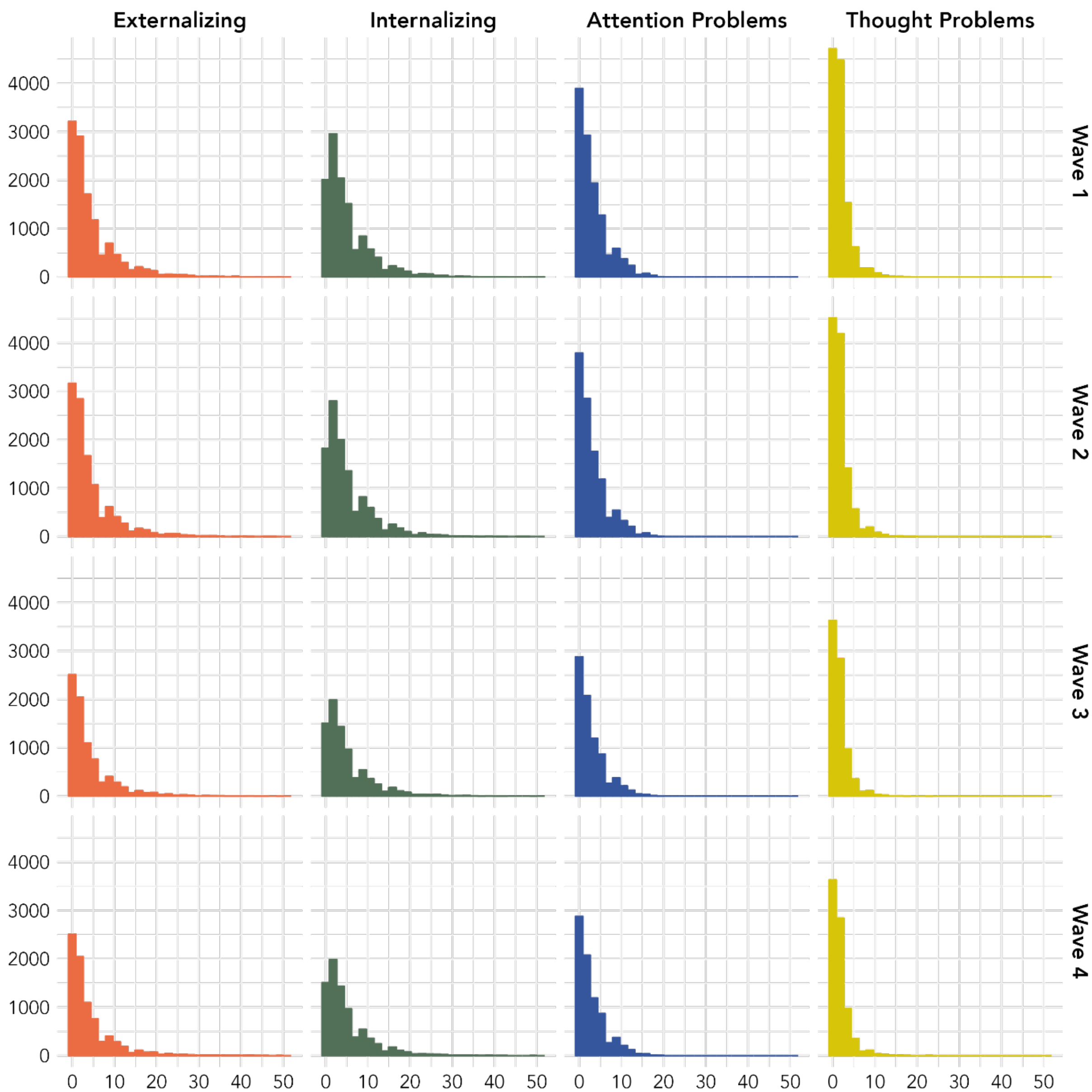

Figure D1. *Univariate distributions of higher-order psychopathology dimensions across all waves.*

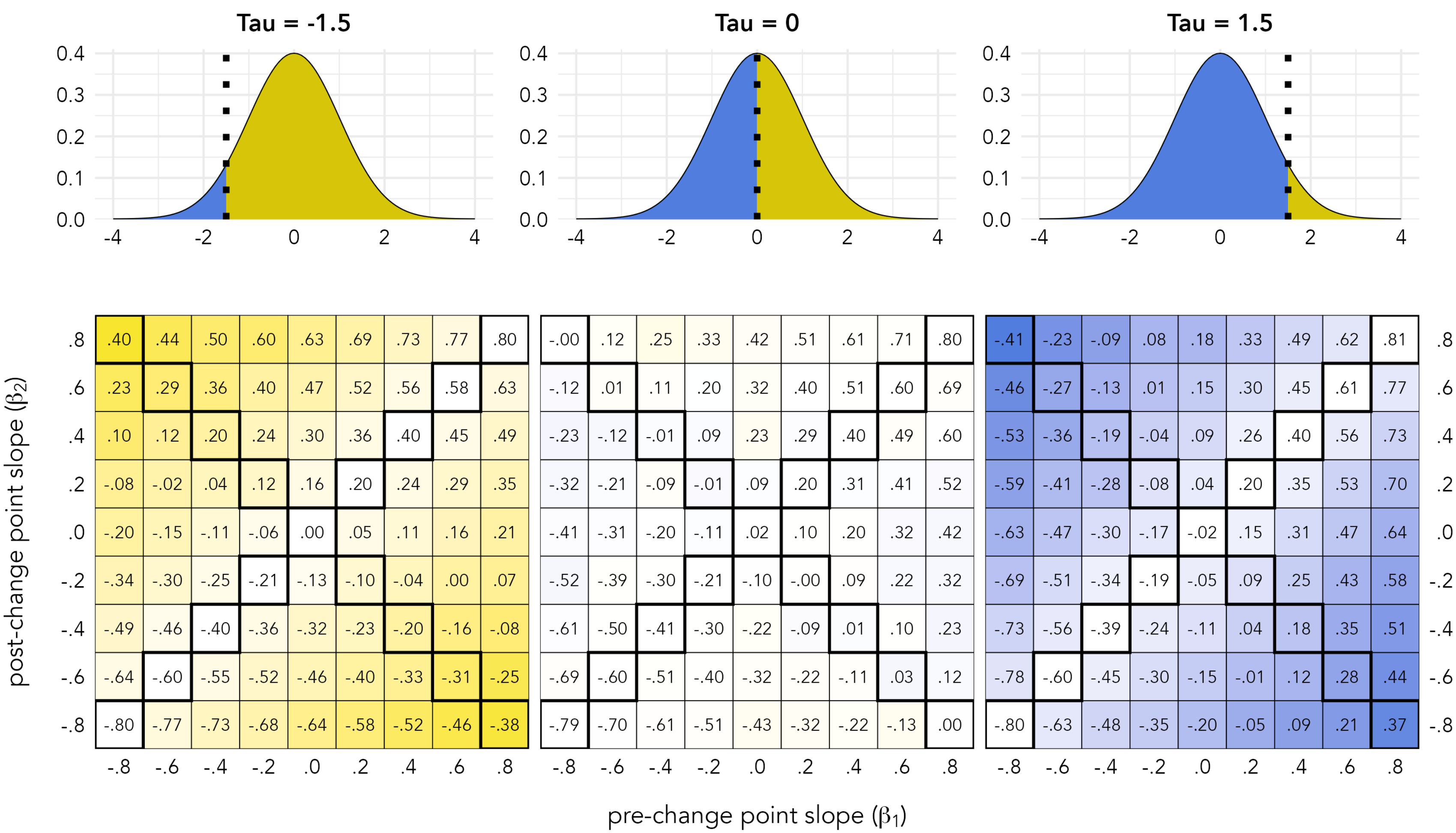

Figure D2. *Heatmaps of the OLS estimate given a 1-change point model with known pre- and post-change point slopes.*

Note. Here, we display the estimated OLS estimate (Pearson r) in each cell of the heatmaps given a known (true) 1-change point model with known pre- and post-change point slopes (or β_1 [x-axis] and β_2 [y-axis], respectively). The density distributions above the heatmaps display differing levels of the change point ($\tau = -1.5, 0, 1.5$). The color and darkness of the cells indicate the direction and extent of bias, whereby the correlation favoring the data to the left of τ is shaded blue and the data to the right of τ is shaded yellow. Where $\tau = 0$, the data are evenly distributed to the left and right of τ, therefore the cells are pale in color because the correlation returns the approximate midpoint of β_1 and β_2.

Figure D2 above demonstrates four important points:

1. **Change point models do not inherently produce evidence of nonlinearity:** The bottom left to upper right diagonals across all levels of τ show that there is no bias in the OLS estimate (Pearson *r*) when the pre- and post-change point slopes are equivalent. That is, although we forced a 1-change point model, when we equated the slopes pre- and post-change point (implying a 0-change point model, or an OLS model), the OLS estimate sufficed. That is, if the slope (y ~ x) does not vary as a function of the value of x, the pre- and post-change point slopes are equivalent to the OLS estimate ($\beta_1 = \beta_2 = r$).

   Figure D3 below demonstrates evidence of this claim, showing that near-equivalent β_1 and β_2 values (.49 and .51, respectively) in a 1-change point model correspond to the OLS estimate ($r = .51$).

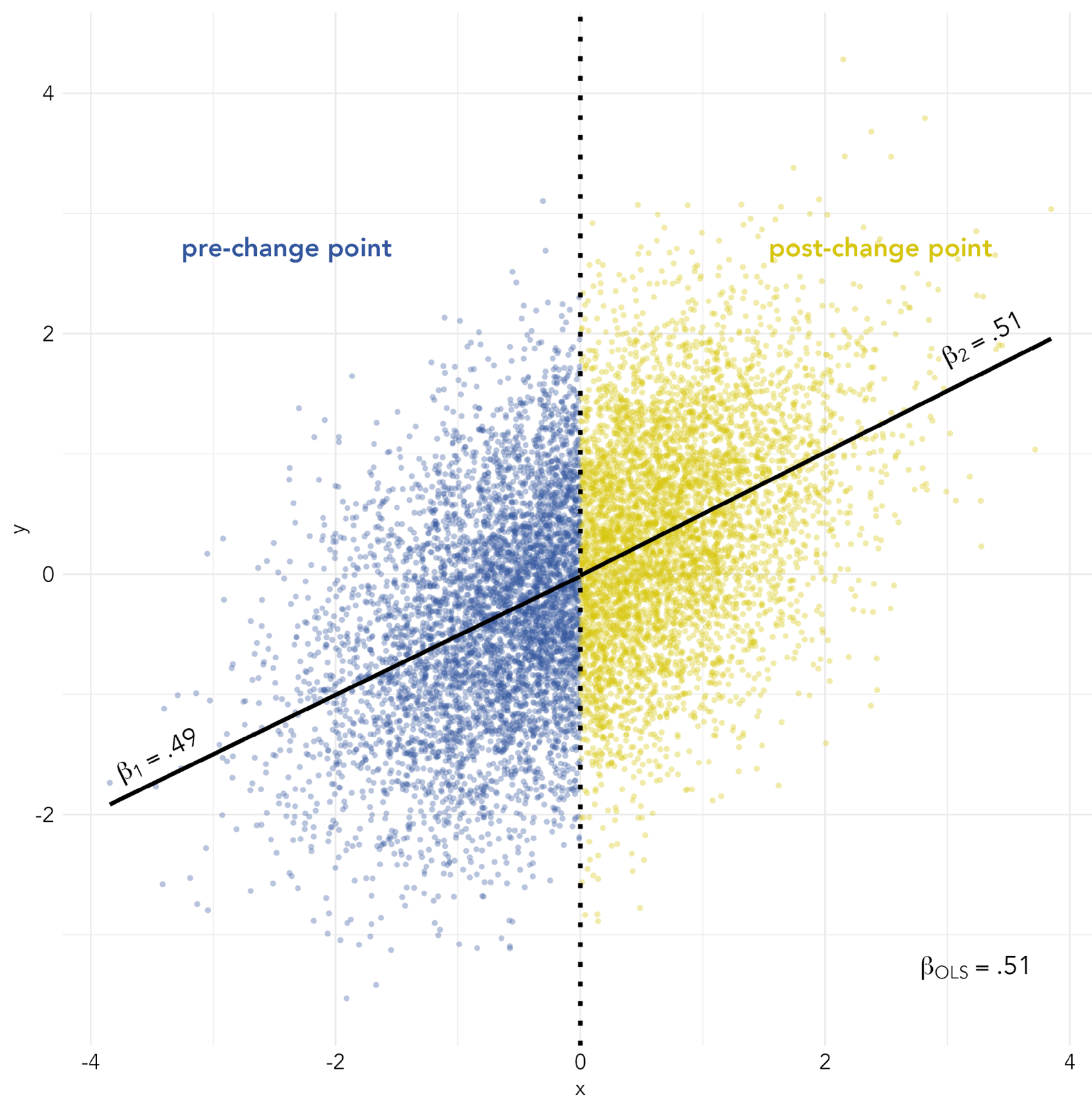

Figure D3. *OLS estimated association between two variables when pre- and post-change point slopes from a 1-change point model are identical (β = .50).*

2. **In the presence of nonlinearity (i.e., a need for a change point), and when the data are evenly distributed pre- and post-change point, OLS returns the average of the pre- and post-change point slopes:** Where τ = 0, ~50% of the data fall pre- and post-change point and the OLS estimate (or correlation) is approximately square in the middle of the pre- and post-change point slopes. For instance, the top left to bottom right diagonal contains cells that are ~0 given that the diagonal captures pre- and post-change point slopes of equivalent magnitude and opposing signs (i.e., β_1 = -.8 and β_2 = .8). Thus, *when data are evenly*

distributed pre- and post-change point, OLS returns the average of the pre- and post-change point slopes.

Figure D4 below demonstrates evidence of this claim, showing that pre- and post-change point slopes that are equivalent in magnitude but opposing in sign (.80 and -.79, respectively) returns an OLS r = .00 when τ = 0. That is, the OLS estimate suggests that there is no association between x and y in the population even though subpopulations have strong positive and strong negative associations pre- and post-change point, respectively!

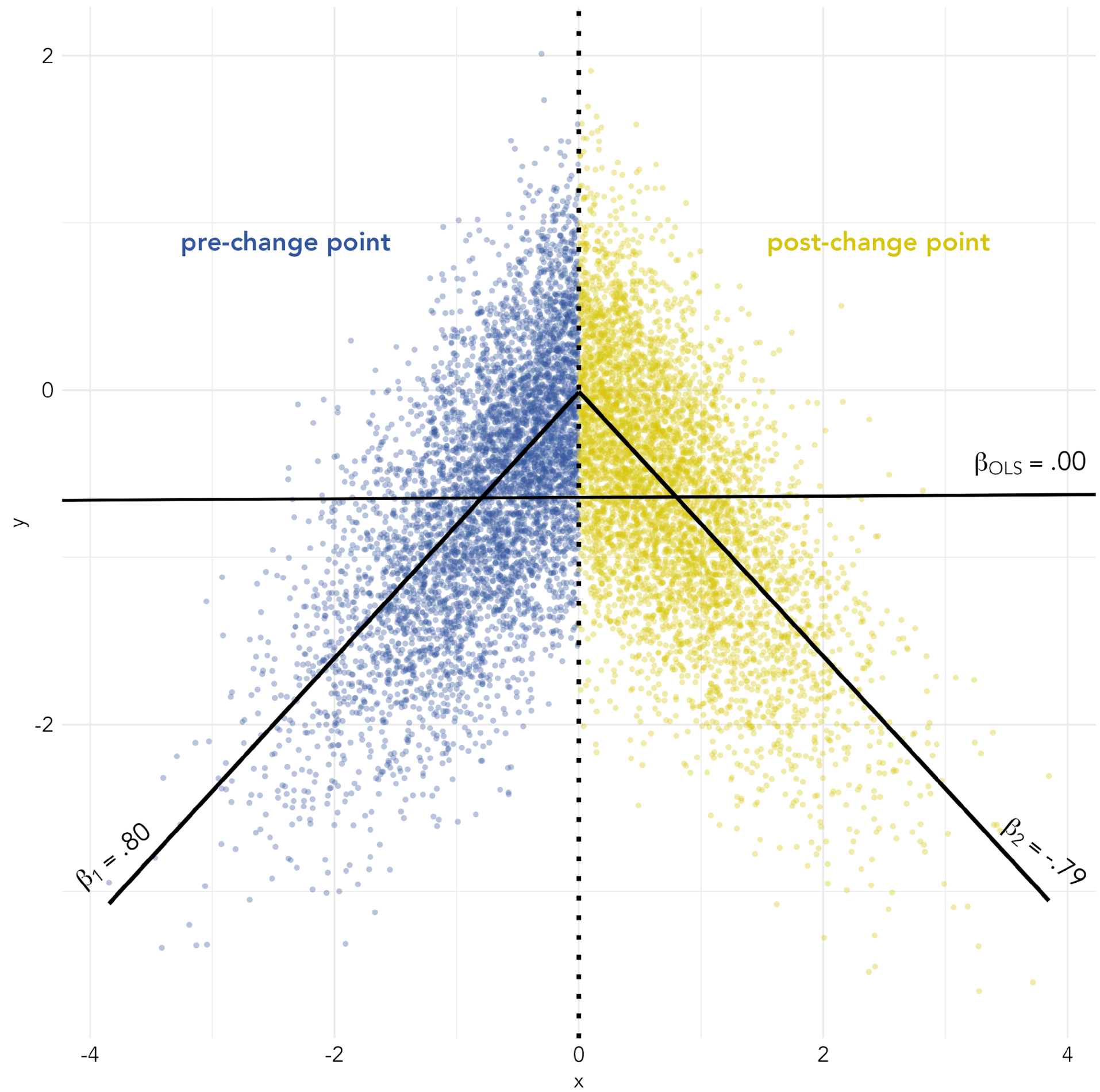

Figure D4. *OLS estimated association between two variables when pre- and post-change point slopes from a 1-change point model are identical in magnitude and opposing in sign (β_1 = -.8, β_2 = .8).*

3. **In the presence of nonlinearity, and when the data are not evenly distributed pre- and post-change point, OLS returns a weighted average of the pre- and post-change point slopes:** Asymmetric distributions (τ = -1.5, 1.5) reveal other useful information. The top left to bottom right diagonal shows that the OLS estimate no longer produces the average (or midpoint) of the pre- and post-change point slopes. Instead, it approximates the weighted average (considering error on both sides of the change point) of the slopes after taking into account the proportion of data on both sides of the change point as well as the respective error in each slope.

That is, because τ determines what proportion of observations fall into each segment, the marginal correlation is expected to reflect a weighted influence of β_1 versus β_2: when τ is far left (e.g., –1.5), most data fall in the post-change region so the OLS r tends to align more with β_2, whereas when τ is far right (e.g., 1.5), the OLS r is driven more by β_1. Hence, Figure D2 panel A (τ = –1.5) is shaded yellow, Figure D2 panel C (τ = 1.5) is shaded blue, and Figure D2 panel B (τ = 0) is hardly shaded such that the OLS r favors both sides of the data equally.

Because τ thresholds a normal predictor, the proportion of observations in each segment depends directly on τ through the standard normal CDF, $P(x<\tau)=\Phi(\tau)$. For example, $\tau=0$ splits the sample roughly 50/50, and $\tau = -1.5$ yields only about 6.7% of observations in the pre segment and 93.3% in the post segment. As a result, the marginal OLS correlation tends to behave like a distribution-weighted blend of the two segment slopes, with the dominant segment (as determined by τ) exerting the strongest influence.

One important consequence of this average is that the OLS estimate can overestimate the population association as a function of zeros. Consider when τ = .5, and ~69% of the data fall before the change-point and 31% fall after it, and when β_1 = .8 and β_2 = .0 (Figure D5 below). By definition, there is no association between x and y post-change point and there is a strong positive association pre-change point. The OLS *r* = .42, which yields the misleading conclusion that there is at least some association between x and y, although it does not exist in a considerable subpopulation. Accordingly, the OLS *r* also suggests that there is a considerably weaker association (moderately positive) between x and y in the subpopulation that corresponds to the lion's share of the population, even though we know the association to be strong and positive.

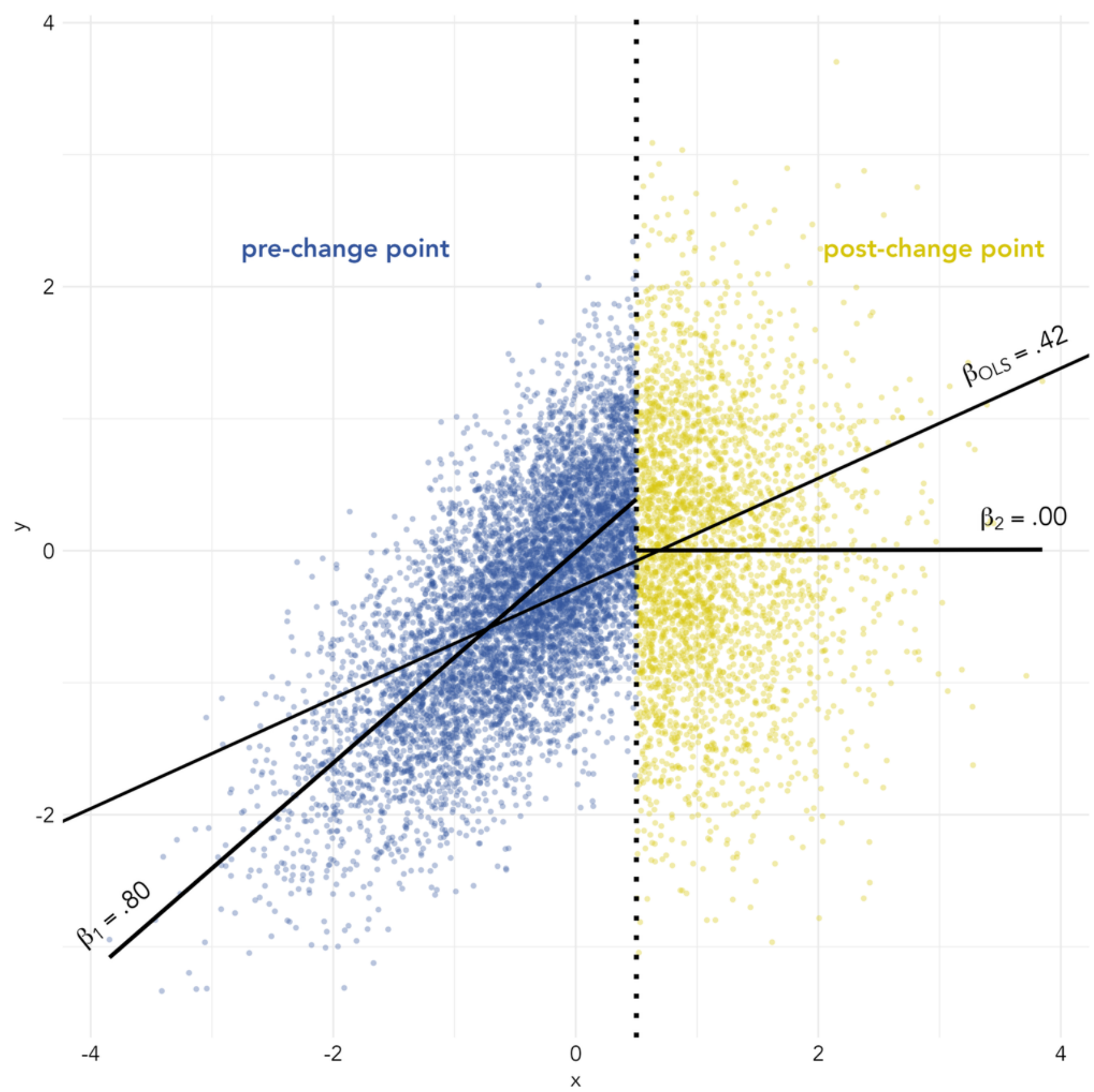

Figure D5. *OLS estimated association between two variables when pre- and post-change point slopes from a 1-change point model are strongly positive and zero, respectively (β_1 = .8, β_2 = .0).*

Although this simulation demonstrates that the OLS estimate in the overall population returns a biased estimate in the presence of nonlinearity, the τ levels selected for this simulation closely correspond to the change point locations identified in our study.

To use a value of τ that is representative of our study's findings, we identify the Z score that corresponds to the value of τ observed in our empirical data. We are borrowing estimates from the model that regressed CBCL Externalizing onto CBCL Internalizing at the baseline wave of the ABCD Study, where a change point occurred at τ = 2.52, or a CBCL Externalizing score of 2.52. Given the mean and SD of CBCL Externalizing (*M* = 4.46, *SD* = 5.87), a score of 2.52 corresponds to a Z score of -.33:

$$z = \frac{X - M}{SD} = \frac{2.52 - 4.46}{5.87} \approx \frac{-1.94}{5.87} \approx -.33$$

We used this Z score, -.33, as the estimate of τ (which is permissible given our simulation conditions) and conducted a novel simulation:

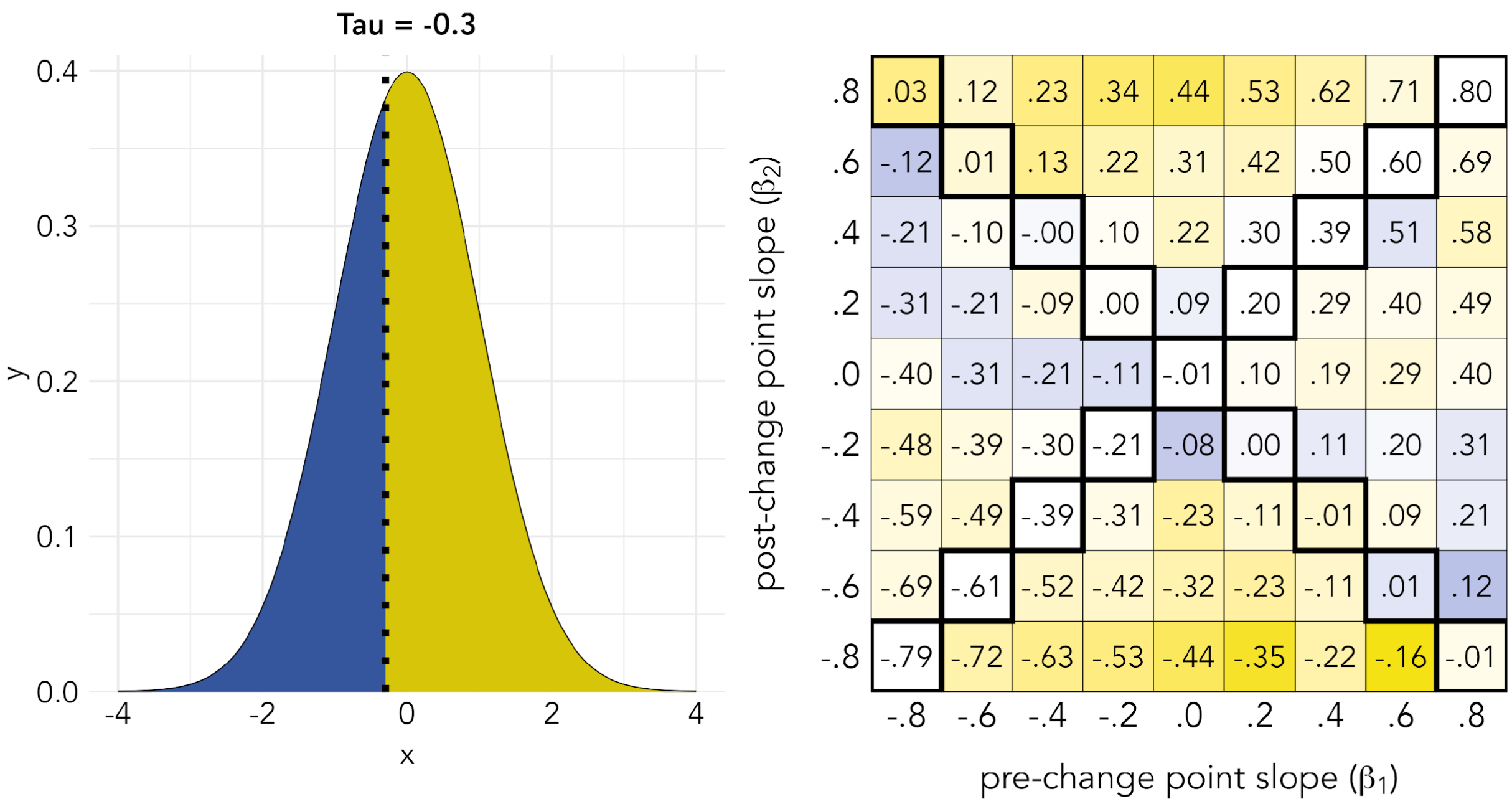

Figure D6. *Heatmaps of the OLS estimate given a 1-change point model with known pre- and post-change point slopes where* τ = -.3.

Note. Here, we display the estimated OLS estimate (Pearson r) in each cell of the heatmaps given a known (true) 1-change point model with known pre- and post-change point slopes (or β_1 [x-axis] and β_2 [y-axis], respectively). The density distributions above the heatmaps display differing levels of the change point (τ = –1.5, 0, 1.5). The color and darkness of the cells indicate the direction and extent of bias, whereby the correlation favoring the data to the left of τ is shaded blue and the data to the right of τ is shaded yellow. Where τ = 0, the data are evenly distributed to the left and right of τ, therefore the cells are pale in color because the correlation returns the approximate midpoint of β_1 and β_2.

As before, we see that the OLS *r* estimate is biased under all conditions, except where $\beta_1 = \beta_2$ (as in the bottom left to top right diagonal). The top left to bottom right diagonal shows just what we demonstrated earlier: The OLS estimate returns essentially the midpoint of estimates when β_1 and β_2 are equivalent in magnitude but differ in sign, at around 0. Thus, under conditions that apply directly to the distributions of the CBCL psychopathology scores and the change points we detected in our study, the OLS estimate is bound to produce a biased estimate of the relationship among higher-order psychopathology dimensions when evidence of nonlinearity exists.

Appendix E

Alternative Estimation Method

We considered an alternative method for identifying nonlinear associations between higher-order psychopathology dimensions using generalized additive multilevel models (GAMM), as in a recent application to psychopathology and cognitive ability.[1] GAMMs combine the flexibility of generalized additive models[2] (GAMs) but can account for random effects in mixed-effect models.

GAMMs use penalized splines to identify nonlinear associations by fitting smooth functions to each predictor while avoiding overfitting the data. We fit cubic splines that form a series of basis functions, the smooth term sums across these basis functions according to how well each of the base functions fit the data locally. The number of basis functions is determined by the total number of knots, or the number of specific points along the predictor variable where the smooth function changes its behavior. Knots are akin to changepoints in Bayesian changepoint models. Knots define the boundaries of the piecewise splines, which are used to model nonlinear associations between the predictor and criterion variables. Increasing the number of knots allows for greater sensitivity to nonlinear trends, but at the cost of potentially overfitting the data.

We fit the splines using restricted maximum likelihood (REML), which has been shown to provide an optimal solution for the penalty term applied to the spline terms.[3] We estimated the GAMM using a spline term for the psychopathology term of interest, a linear fixed effect for age, and nested random effects for the study site and family identification variable to account for ABCD's complex survey design. We fit GAMMs using the gam function in the mgcv[4] package using R version 4.3.0[5] and allowed for 4 through 15 knots. We focused on the association between externalizing and internalizing at wave 1, with the former being the criterion variable and the latter the predictor variable.

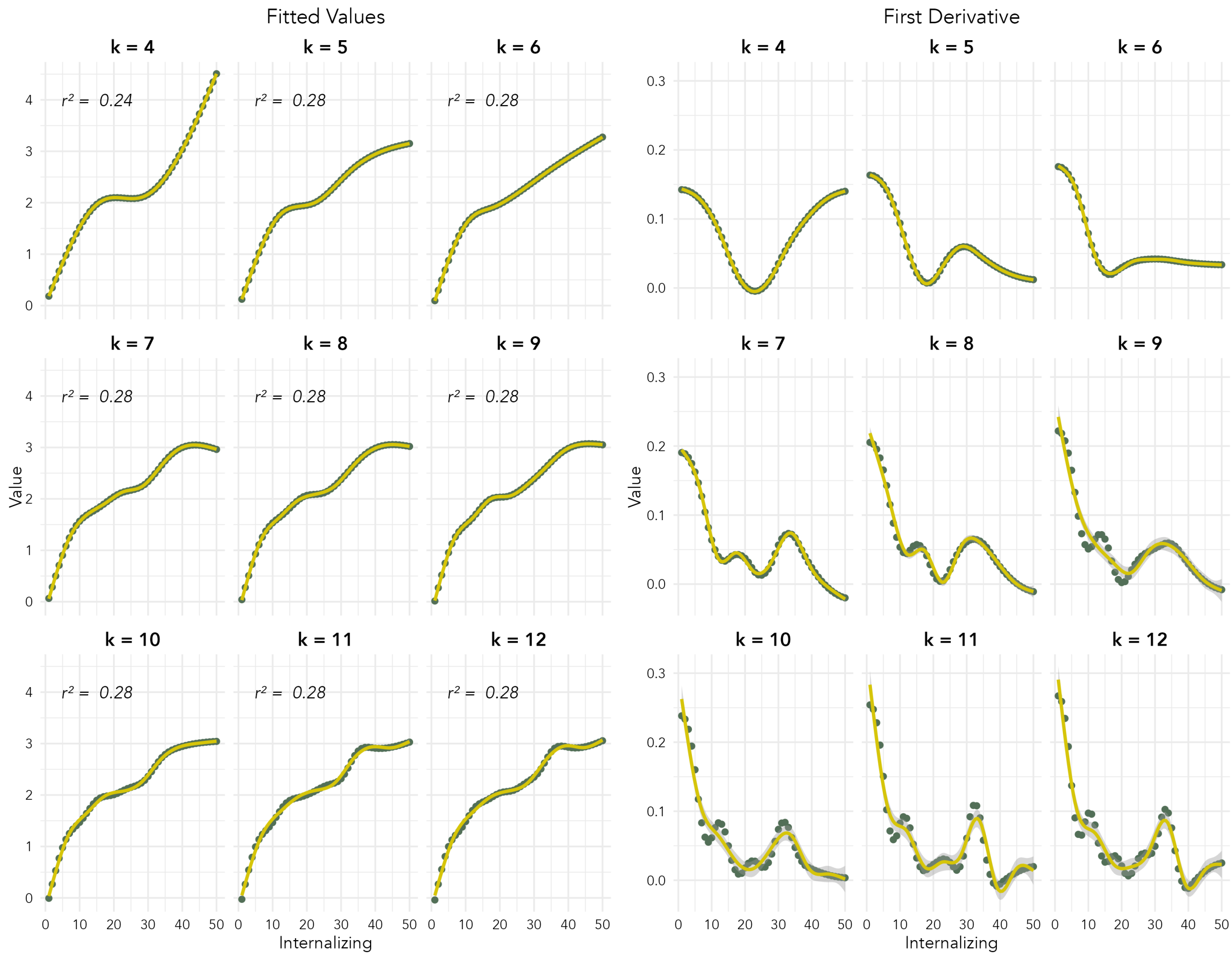

GAMM models suggested similar patterns across models with 4 through 15 knots. First, the models clearly detail a greater first derivative in the lower range of the predictor variables (where Internalizing < 8) and a diminishing first derivative in the upper range of the predictor variables. This trend suggests that the association between externalizing and internalizing decreases as internalizing symptom endorsement increases.

The model R^2 values detail the optimal number of knots, which occurs at k = 5. All models with 5+ knots show no incremental gain in R^2.

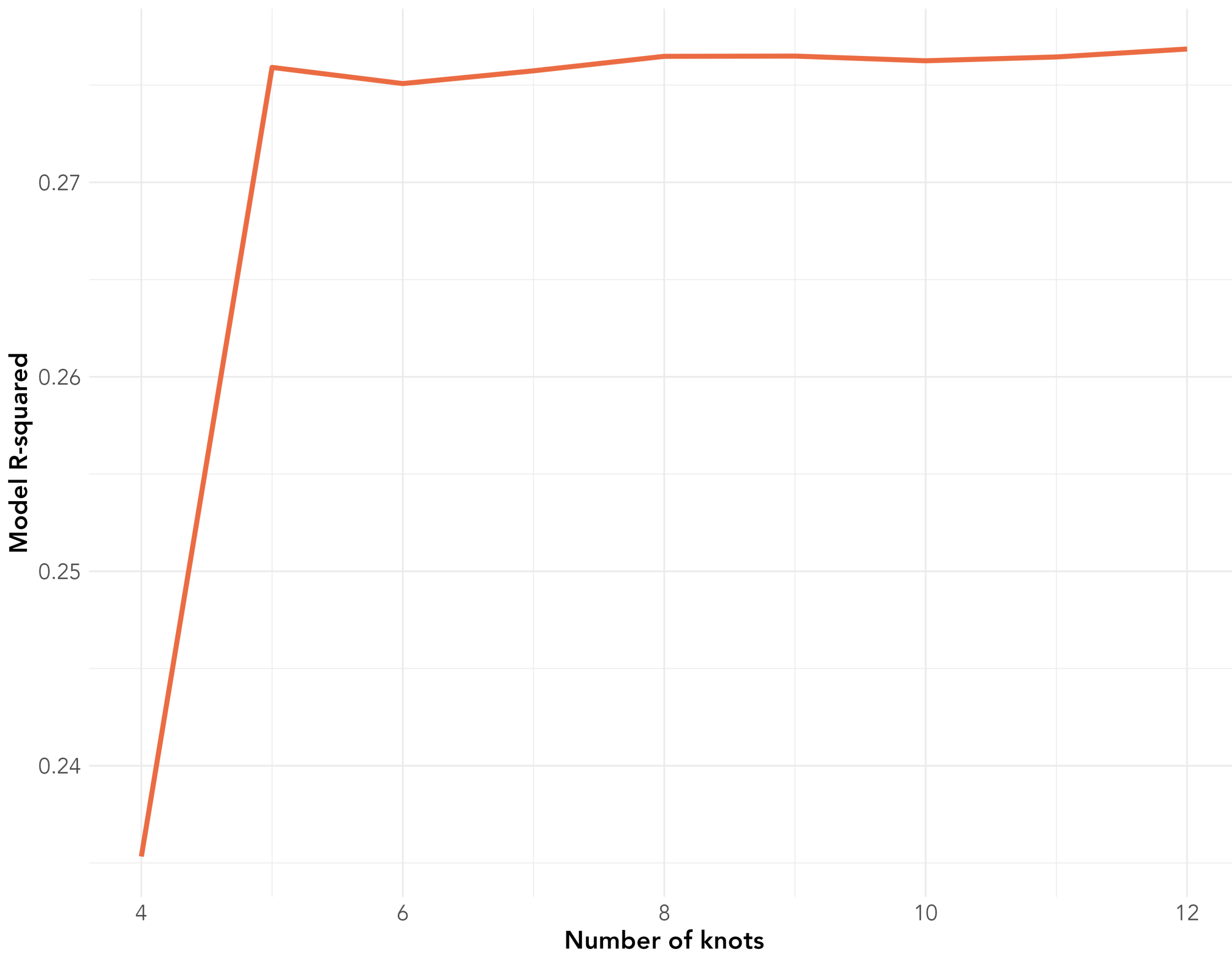

Additionally, increasing the number of knots seems to transition the general pattern of the spline term from cubic (k = 4) into something closely mimicking the changepoint model (k > 4). That is, there was a strong positive slope before the changepoint (knot), and a decrease in the slope after the changepoint (knot), where the slope remained static after the changepoint.

Where k > 6, the models were clearly overfit, consistent with criticisms of GAMMs, and the models appeared biased by outliers at extreme levels of internalizing. Critically, there was no evidence that the association between externalizing and internalizing was strong and positive at higher levels of internalizing.

References

1. Pines, A. *et al.* Psychiatric symptoms, cognition, and symptom severity in children. *JAMA Psychiatry* **81**, 1236 (2024).
2. Hastie, T. & Tibshirani, R. *Generalized Additive Models*. (Routledge, Taylor & Francis, London, 2017). doi:10.1201/9780203753781.
3. Wood, S. N. *Generalized Additive Models: An Introduction with R*. (Chapman and Hall/CRC, 2017). doi:10.1201/9781315370279.
4. Wood, S., & Scheipl, F. Generalized Additive Mixed Models using 'mgcv' and 'lme4'. (2020).
5. R Core Team. R: A language and environment for statistical computing. Foundation for Statistical Computing (2020).
