# Supplementary material for: Covariation among higher-order psychopathology dimensions is driven by nomorbidity and not comorbidity
Source: Nat Ment Health. 2026 Oct 1;4(11):1674–85. doi: 10.1038/s44220-026-00727-0 (PMC13736491; doi:10.1038/s44220-026-00727-0)
Supplement: Supplementary file 3 [file 44220_2026_727_MOESM3_ESM.pdf]

nature portfolio

Peer Review File

Covariation among higher-order psychopathology dimensions is driven by nomorbidity, not comorbidity

Corresponding Author: Dr Ashley Watts

Parts of this Peer Review File have been redacted as indicated to maintain the confidentiality of unpublished data.

This file contains all reviewer reports in order by version, followed by all author rebuttals in order by version.

Version 1:

Reviewer comments:

Reviewer #1

(Remarks to the Author)
In the manuscript titled "Covariation among higher-order psychopathology dimensions is driven by nomorbidity, not comorbidity", the authors examined associations among higher-order psychopathology dimensions in the ABCD study. They found strong associations among these dimensions at low levels of externalizing and internalizing symptom burden, but weaker associations at higher, symptomatic levels. Overall, I found the research question both important and timely, and the study will likely interest a wide range of researchers, including clinicians. The work has several notable strengths, including its robust statistical approach and large sample size. However, I had several questions, comments, and concerns while reading the manuscript, which I have detailed section by section below.

Introduction

The following statement in the Introduction section of the manuscript (line 23-24) needs a citation: "But in the general population, it is statically uncommon to be diagnosed with 2 or more conditions, let alone 3 or more." Where did the authors get this statistic from? The DSM? Previous research?

Given the authors are introducing a more novel, or less commonly used, phrase in this manuscript, I found myself wanting a more thorough definition of nonmorbidity when it is first introduced in the Introduction section of the manuscript (line 26).

I had a question in response to line 57 in the Introduction section of the manuscript. If asymptomatic cases in the population drive the rates of comorbidity, why are people more likely to be diagnosed with 2nd diagnoses after receiving their first (mentioned on line 31 by the authors)? These two points seem to be in contradiction to one another, and I think a more nuanced explanation is required to explain how these can both be true.

A mention of the age onset of externalizing and internalizing disorders in youth at the ages sampled in ABCD study (perhaps as described in the DSM or from another source/research) would be helpful for contextualizing your results. Would we expect a certain percentage of kids at this age to have more severe or symptomatic internalizing or externalizing symptom burden (or both)?

As a reader, I found that the use of quotations in the Introduction section of the manuscript occasionally interrupted the continuity of the text. Paraphrasing these points could help strengthen the author's own voice and argument early on.

Methods

How were symptomatic levels defined? This term was used in the Abstract, but I am not sure how it is defined. I see how asymptomatic is defined (e.g., 3-5 symptoms), so I think symptomatic should also be operationalized.

I think the authors need to make a better argument for why the ABCD dataset is an appropriate or fitting sample for this type of research question. There needs to be clearer motivation for studying this research question in children vs. adults. I think this could be addressed and explained in Methods and Introduction section of the manuscript.

A citation is needed for line 81-82: “The CBCL is among the most widely used assessments of youth psychopathology.”

I was curious why the authors used the CBCL over other mental health measured in the ABCD study protocol, including the diagnostic interviews (K-SADS)?

How would the results potentially change if the authors used multiple mental health measures specific to certain diagnoses rather than using sub scores of the same survey? (e.g., Conners). Did the authors consider using the sub-scales within the internalizing and externalizing CBCL scores (e.g., Anxious/Depressed, Aggressive Behavior)?

I believe there needs to be a stronger explanation in the text for starting with internalizing and externalizing sub-scales and then moving to thought problems and attention problems scales.

Results

In line 181-182, the authors describe the percentage of participants at wave 1 who had nonclinical levels of internalizing and externalizing and the percentage of the sample who exhibited some combination of at-risk or clinical levels of both internalizing and externalizing, but the percentage does not add up to 100%. What about the other percentage of participants?

Discussion

One thought I had while reading the Discussion section of the manuscript was, is there less comorbidity seen in the more symptomatic cases because there are less people with high symptom burden and more people with lower symptom burden in the sample? What if there was more variability in the higher end of the scale? What if it was an older sample, or a more clinical sample?

I think more discussion on the results in the context of the sample would be beneficial- this study is in children, yet the discussion doesn’t necessarily focus on the comorbidity of mental health challenges in children vs. adults.

The Discussion (and Introduction) could benefit from section headings to guide the reader through the main arguments and improve the overall flow.

Conclusions
Are the authors able to cite examples of previous work where comorbidity was driven by lack of mental health burden rather (i.e., nonmorbidity) rather than prevalence of multiple mental health challenges (i.e., comorbidity)? This research is alluded to but not necessarily discussed in detail.

Are the authors able to comment on what they see as the reason for the disconnect between the results of this research and what is happening in the clinic? The authors themselves said that there is an increased risk of a second mental health diagnosis after the first is received, but if comorbidity in research is driven by low burden cases, what is happening in the clinic to cause this comorbidity phenomenon in the real world? A discussion on this could be helpful to readers who are clinicians.

Figures

Text for figure 2 was blurry and hard to read, especially the white text on the yellow background.

(Remarks on code availability)

Reviewer #2

(Remarks to the Author)
Using the ABCD data, the authors examine non-linear relations between internalizing and externalizing symptoms to provide evidence that comorbidity between internalizing and externalizing (and symptoms generally) is primarily driven by the absence of symptoms.

I find the paper to be interesting and novel. The authors appear to provide sufficient rationale for the Bayesian approach and provide supplemental analysis for alternative approaches. I had a few comments, questions, and suggestions that I hope the authors find helpful.

1. Given some of the authors main points regarding the need to go beyond the GLM, with a particular critique of the common factor model, I was surprised there was not more conversation about similar issues that have already been examined when the common factor model is considered within the IRT literature. The authors do cite one of the articles that came to mind:

78. Wall MM, Park JY, Moustaki I. IRT modeling in the presence of zero-inflation with
572 application to psychiatric disorder severity. Appl Psychol Meas. 2015;39(8):583-597.

But the reference to this work is quite brief:

"Such an estimate is not only an oversimplification of the true population relationship,
but a biased estimate of it"78–81

At least at one point in the manuscript, the authors seem to suggest that psychology has not really considered the issues the authors are trying to highlight regarding distributions of the data and base rates and implications for covariance and so on, e.g.,

"Covariance-based similarity measures are dramatically influenced by the distributional
qualities (e.g., base rates, skewness) of the variables of interest64, an issue that has been better appreciated in other fields (e.g., ecology, computer science, engineering)."

And I would generally agree with this claim.

However, there has been several within the IRT literature that have made similar points when discussing the precursor to covariation between two constructs, which is how to estimate variation for one construct, which in itself is focused on covariation among indictor items for a given construct. Within IRT, this often involves deriving some theta score on some construct, and this theta is usually derived using the common factor model critiqued by the authors and the assumptions that go with it (e.g., linearity within a logistic scale assuming proportional odds within a graded response model for ordinal items).

Similar to the authors critiques, many within the IRT literature have noted the issue of the "quasi trait" or unipolar nature of many constructs relevant to psychopathology (e.g., depression) and have tried to come up with alternatives.

For example, the Wall et al. paper cited in the manuscript uses zero-inflated mixture modeling, where IRT parameters are only estimated for the "traited" class (since Wall et al., like the authors here, are concerned about the impact of all the observations with no symptoms - a factor, after all, just captures covariation among a set of measures thought to assess a single construct).

I am also familiar with some additional alternatives to the common factor model (Reise, S. P., Rodriguez, A., Spritzer, K. L., & Hays, R. D. (2018). Alternative approaches to addressing non-normal distributions in the application of IRT models to personality measures. Journal of personality assessment, 100(4), 363-374. may be a helpful reference), such as Lucke's unipolar log-logistic model and Ramsay-Curve IRT and so on. Again, the individuals contributing to this IRT work seem to make points similar to the authors here,

"More generally, we call for a reconsideration of uncritical adoption of the general linear model in psychopathology research, which often produces comorbidity-inference artifacts in community-level data, where most individuals do not exhibit clinically severe psychopathology. We encourage researchers to embrace nonlinearity and population heterogeneity and adopt conceptual and analytic frameworks that prioritize symptomatology over statistical correlation."

However, the rub is, best to my knowledge, when these alternatives are implemented (such as the Wall et al. approach) and the thetas are calculated, they give thetas that correlate very, very highly with the thetas obtained by the common, "flawed" approaches.

For example, per Reise et al., using the more complicated zero inflated mixture model advocated by Wall et al. will give a theta that correlates .994 with the "flawed" graded response model implied by the common factor model. I think the one exception (at least among those highlighted by Reise) is the log logistic model, where Reise found thetas from this approach "only" correlated with .72 with the graded response model estimates.

All to say - it seems that many of the issues that the authors are highlighting here in terms of covariation have been highlighted regarding variation within the IRT literature (which is also focused on covariation, just at the indicator level). For me, they are nearly identical issues - a factor model relative to IRT involves linking together, for psychopathology, a set of symptoms (where many people have 0 values) - and covariation between different types of psychopathologies (e.g., internalizing and externalizing when using a lumped approach) involves linking together scores based on a model (usually the common factor model).

In my opinion, I would think one would want to puzzle out how to best model variation within a construct (i.e., puzzle out how to best model covariation between indicators of a construct) before scrutinizing the covariation between constructs. Unfortunately, we haven't really done that as a field, and thus the importance of the current work to challenge commonly held beliefs about comorbidity.

That being said, I think the authors could more strongly tie in work conducted within IRT that have grappled with similar issues and the practical limitations discovered in this work (e.g., technically more correct models that produce nearly identical thetas) and the implications of such work to the current focus on covariation (e.g., perhaps the highly correlated thetas from say a zero-inflated Wall et al. mixture still has utility for the issues discussed here, since that approach would let you remove the "traited" from the others before proceeding to covarying with something else).

Indeed, given the current work has arguably a more satisfying empirical result compared to some alternatives highlighted in

IRT (with due respect to everyone, I do not find it that satisfying, in most contexts, to trouble with a zero-inflated mixture model IRT to get thetas that correlate .994 with the graded response model), perhaps the current work has implications if one where to take a step back and consider how to best measure the constructs we seek to ultimately covary.

2. Related to point 1, I wasn't quite sure what the way forward is per the authors beyond the general conclusion is we should be doing something different than we are typically doing, and that different thing probably won't be limited to linear relations. For example, on line 283, the authors state "there may be no single, “true” structure of psychopathology in the population" - if this is true, what is the way forward for say DSM-6 or individuals who work and train in the applied branches of clinical psychology (e.g., see patients) and have to seek training in things like psychopathology and learn to yoke said psychopathologies to say evidence-based treatments?

3. Related to point 1 and 2, I am not quite sure what the authors are suggesting for alternative measurement models to constructs related to psychopathology, particularly in light to the existing IRT literature that went down this road and primarily found thetas that correlate really, really highly with standard approaches.

My read of the discussion is that I get reminded that comorbidity is probably driven by the zero cases (just as the factor loadings for a given construct's measurement model is probably driven by the zero cases on the indicators), and that other fields think of this better than we do - we have the p-factor after all - then on line 264 we seem to get to the measurement model issue and note the assumptions (as is done in Wall et al. and the other IRT papers I mention) and how things like average can be misleading (similar to points from the IRT literature, e.g., “it makes little or no sense to assert that a person has below-average level of addiction to alcohol or an above-average level of addiction to gambling”, Lucke, 2015), then some info on how configural invariance across populations might be hosed, then some reasonable defense of study limitations, and then back to how we need to do something different, something nonlinear. But what do we do, exactly?

To put it another way, I have heard this (sort of) all before from the IRT literature, and I find the arguments compelling, but I have not found the nonlinear alternatives findings to be that satisfying - what is different here?

4. If there is to be revolution for how we view comorbidity, it would seemingly start with a revolution in our measurement models, but I am not quite sure what the authors have in mind here or how that would be different from what has been proposed and tested. (to be clear, I don't think it is the authors' charge to solve this, but perhaps something more concrete would be helpful).

In the meantime - what do we do? It seems a very practical implication is that if the field is going to continue to use the common factor model and extensions (e.g., sum scores), which seems to be a solid bet, the least we could do is add say a quadratic term when say regressing internalizing on externalizing to see how often the relation dampens as we get higher in X...do the authors agree with this? And again, if not this, what?

(Remarks on code availability)

Reviewer #3

(Remarks to the Author)
This manuscript examines patterns of associations between dimensions of psychopathology by challenging multiple assumptions espoused in dimensional models of psychopathology. The authors are very clear in their description of the problem. Whether others agree with the premise or not, the authors make cogent arguments based on transparent observations of historic and the analyzed data. If readers and reviewers react negatively to the work, I suspect that these arguments are hitting on fundamental assumptions that shake a compelling narrative, rather than the evidence. The supplementary material provides a great set of complementary analyses that address the low hanging comments that could be made. That said, I am challenged to find many areas of the manuscript to critique. I have some thoughts for the authors to consider, but I do not know that all may lead to a cleaner manuscript.

Perhaps one quibble is in terminology. The authors propose the concept of ‘nomorbidity.’ Is this premise based on the massive center of data with no endorsement of psychopathology, or the degree of association between the internalizing and externalizing dimensions? If the former, I see the naming. However, if the latter, I wonder if this space of the distribution would be better termed ~low-morbidity? That is, the associations are stronger at lower levels, but variability is needed in order to quantify the relation.

The authors rely on change point models to evaluate the non-linear associations in the sample. The complement of GAMMS is complementary and show This is quite clear. The context of the work and focusing on dimensionality leads to an intuitive understanding of these results. Is there an alternative explanation that these relations may be better conceptualized as subgroups coming from different portions of the population? Is there a way of reconciling those different perspectives?

The data came from a community-based sample and a second change point was not identified in the models. Is it possible

that a sample with a higher level of severity may show a second inflection point at higher levels of severity? Might this dovetail with the argument that there is not a uniform psychopathology structure in the population?

For some normative anchoring, it could be helpful to link raw scores to percentiles or t-scores.

(Remarks on code availability)
The code is clear in its uses and structure.

Version 2:

Reviewer comments:

Reviewer #1

(Remarks to the Author)
Thank you for the thorough revisions and detailed responses to my previous comments. I appreciate the authors' careful engagement with the review and believe the manuscript has been substantially strengthened. In particular, the additional analyses using the adult caregiver sample, the expanded justification for the analytic approach, and the added discussion clarifying the distinction between population-level covariance and clinically observed comorbidity address many of my original concerns. Overall, I believe the manuscript is much improved and ready for publication.

(Remarks on code availability)

Reviewer #2

(Remarks to the Author)
I thought the authors did an excellent job addressing reviewer comments from the first round of reviews. I am hoping the issues highlighted here and the approaches promoted by the authors are put into play within various fields relevant to psychopathology.

(Remarks on code availability)

Reviewer #3

(Remarks to the Author)
This is a comprehensive revision and response to previous review comments. The text clarifications and additional supplementary material are helpful to make the already strong manuscript stronger. I have no further queries for the authors.

(Remarks on code availability)
The code and material from the github repository are clear.

Open Access This Peer Review File is licensed under a Creative Commons Attribution 4.0 International License, which permits use, sharing, adaptation, distribution and reproduction in any medium or format, as long as you give appropriate credit to the original author(s) and the source, provide a link to the Creative Commons license, and indicate if changes were made.
In cases where reviewers are anonymous, credit should be given to 'Anonymous Referee' and the source.
The images or other third party material in this Peer Review File are included in the article's Creative Commons license, unless indicated otherwise in a credit line to the material. If material is not included in the article's Creative Commons license and your intended use is not permitted by statutory regulation or exceeds the permitted use, you will need to obtain permission directly from the copyright holder.
To view a copy of this license, visit https://creativecommons.org/licenses/by/4.0/

Response to Reviews

We sincerely appreciate the opportunity to revise our manuscript and endeavored to be as responsive as possible to the helpful and constructive feedback from the editors and reviewers. Before we address each comment point-by-point, we have summarized the major changes to the manuscript:

1. **The appropriateness and novelty of the Bayesian change point model over existing OLS regression and non-normal item response theory (IRT) techniques:** R2 pointed out (see R2 #2,3,5 below) that existing IRT techniques have accommodated non-normal distributions but have not been widely adopted because they produce results (distributions of factor scores) that correlate nearly perfectly with those derived from IRT methods that assume an underlying normal distribution. We addressed this feedback in two major ways.

   First, we have added a simulation study (summarized in Appendix E, which is included at the end of this document for convenience) that establishes that the ordinary least squares (OLS) regression is biased in the presence of nonlinear relationships among two variables. In short, we simulated data from a 1-change point model that establishes that – in the presence of nonlinear relationships among two variables – the OLS estimate (e.g., a slope from a simple linear regression, and by extension the Pearson's correlation) is associated with considerable bias. For instance, in extreme conditions where the pre- and post-change point slopes are strong and equivalent in magnitude but differ by sign (e.g., $B_1 = -.8$, $B_2 = .8$), the OLS model estimates a null ($B = 0$) association. In other conditions, such as where there is a strong association pre-change point and a null association post-change point (e.g., $B_1 = -.8$, $B_2 = 0$), the OLS model estimates the average of these two associations ($B = -.4$), which characterizes neither the pre- or post-change point association and produces a misleading positive association post-change point when we can establish that one does not exist. Appendix E details many such scenarios.

   Critically, we also establish that the OLS estimate is unbiased when the 1-change point model includes pre- and post-change point slopes that are identical in magnitude (i.e., there is no evidence of nonlinearity). Otherwise, in the presence of nonlinearity, the OLS estimate is always biased, with the degree of bias varying as a function of the extent of nonlinearity. Thus, the change point model does not always produce evidence of nonlinearity – it only does so when there is genuine evidence of it.

   Second, R2 makes the interesting point that non-normal IRT methods already exist and have not been widely adopted because they have produced unsatisfying results that do not appear to increment existing methods that assume an underlying normal distribution. Although we agree in principle, we advance several counterpoints and demonstrate that the conclusion that factor scores from nonnormal and conventional IRT approaches yield highly correlated factor scores is flawed because the correlation still assumes a linear relationship between the factor scores. We first expand upon a point made in the original submission, that the data used here violate fundamental assumptions of the linear model, precluding their use even if the linear model is popular. Originally, we showed these violations for externalizing and internalizing dimensions but have expanded our analysis to examine all pairwise combinations of higher-order psychopathology dimensions (Appendix B, Figures B9-10).

   Further, in a novel demonstration analysis, we show that factor scores from the two IRT approaches have a nonlinear relationship, with strong convergence between the factor scores arising at low but not high levels of the variables of focus. In fact, there was a weak relationship

among factor scores at moderate to high levels. These results strongly align with our argument that covariance (i.e., the correlation) is driven by zeros. ***Our full response to R2's helpful points can be found in R2 #3.***

2. **<u>Our exclusive focus on youths and potential limitations in generalizability to adults</u>**: R1 raised the reasonable point that our results may not generalize to adults. We present two sets of evidence demonstrating that developmental differences do not drive our results, because the mechanism is distributional (not developmental).

   First, in the revised manuscript, we have now analyzed data from the adult caregivers who self-reported on their own psychopathology per the Adult Self-Report (ASR), the adult counterpart to the Child Behavior Checklist, at wave 1 of the study. We now report these results in Appendix C (Table C9) and show below (see R1 #5) that the results from adult caregivers are consistent with what we observed in youths. That is, there was a strong positive relationship among externalizing and internalizing dimensions at low levels and a weak relationship at moderate-low to high levels. With these findings in mind, we have revised the manuscript to summarize evidence of generalizability across development but have largely maintained a focus on the youth results in the main text given space constraints.

   Second, it is worth noting that our simulation study (summarized in point #1 above) establishes that our youth results were not driven by developmental differences between youths and adults *per se*. Instead, evidence of nonlinearity is driven by the skewed distribution of the data. When adult data are positively skewed (as is most often the case in psychopathology, even when dimensional assessments are used), the OLS model runs the risk of being biased and failing the assumptions of the linear model. ***Our full response to R1's comments can be found in R1 #5.***

3. **<u>Our focus on higher-order as opposed to lower-order psychopathology dimensions:</u>** R1 requested more justification for why we started with externalizing and internalizing dimensions, as opposed to lower-order psychopathology dimensions. Part of the justification for focusing on higher-order dimensions was that these are the dimensions directly implicated in operationalization of the *p*-factor, and most saliently demonstrates the applied issues we empirically evaluate in the current work. In addition to making changes to the text, we have now incorporated analyses that regressed lower-order psychopathology dimensions onto one another. We report these results in Appendix C (Table C8). In short, findings for lower-order dimensions were with few exceptions consistent with those for higher-order dimensions. We now summarize these findings in a footnote in the revised text and include in the future direction section in the discussion a nod to these findings and encourage researchers to further explore the extent to which lower-order dimensions' associations are also driven by the inclusion of asymptomatic cases. ***Our full response to R1's comment can be found in R1 #12.***

4. **<u>The tension between clinical observations of comorbidity and our conclusion that covariance is driven by nomorbidity rather than comorbidity</u>**: R1 asked us to comment on the "disconnect" between our findings and what is happening in the clinic, where it is common to find individuals who are diagnosed with two or more conditions at once. The new Appendix A (referred to in a footnote in the main text) details how the OLS model is dominated by the highest-density region of the data, which corresponds to low levels of psychopathology in the presence of extreme positive skew (as in most community-based studies). In contrast, we detail how clinic-level observations of comorbidity are driven by clinical ascertainment, which conditions on the presence of at least some symptomatology. Thus, these two sets of

observations are simply driven by different pieces of information, or levels of analysis. For convenience, we have included Appendix A at the end of this document. ***Our full response to R1's comment can be found in R1 #18.***

Point-by-point Response to Reviews

Reviewer 1

R1 #1: In the manuscript titled "Covariation among higher-order psychopathology dimensions is driven by nomorbidity, not comorbidity", the authors examined associations among higher-order psychopathology dimensions in the ABCD study. They found strong associations among these dimensions at low levels of externalizing and internalizing symptom burden, but weaker associations at higher, symptomatic levels. Overall, I found the research question both important and timely, and the study will likely interest a wide range of researchers, including clinicians. The work has several notable strengths, including its robust statistical approach and large sample size. However, I had several questions, comments, and concerns while reading the manuscript, which I have detailed section by section below.

Response: Thank you so much for your constructive feedback. We have addressed each comment below.

R1 #2: The following statement in the Introduction section of the manuscript (line 23-24) needs a citation: "But in the general population, it is statically uncommon to be diagnosed with 2 or more conditions, let alone 3 or more." Where did the authors get this statistic from? The DSM? Previous research?

Response: Thank you for the opportunity to clarify. We have added a citation and have clarified that we are referring to epidemiologic data. Specifically, we referred to data from the National Comorbidity Survey, an epidemiologic study:

> **p. 3:** *"But epidemiologic data suggest that it is statistically uncommon to be diagnosed with 2 or more conditions, let alone 3 or more, at the same time[3]."*

R1 #3: Given the authors are introducing a more novel, or less commonly used, phrase in this manuscript, I found myself wanting a more thorough definition of nonmorbidity when it is first introduced in the Introduction section of the manuscript (line 26).

Response: We have expanded our definition of 'nomorbidity,' but it remains relatively succinct. Our definition remains succinct, in part, because definitions of its counterpart, comorbidity, are also succinct: diagnosing with two or more conditions at a given time.

> **p. 3:** *"Around 70% of the population exhibits what we term 'nomorbidity' – wherein an individual is not diagnosed with a single mental disorder – when past-year diagnoses are assessed and 50% when lifetime diagnoses are assessed[3]."*

<u>R1 #4</u>: I had a question in response to line 57 in the Introduction section of the manuscript. If asymptomatic cases in the population drive the rates of comorbidity, why are people more likely to be diagnosed with 2nd diagnoses after receiving their first (mentioned on line 31 by the authors)? These two points seem to be in contradiction to one another, and I think a more nuanced explanation is required to explain how these can both be true.

<u>Response</u>: We appreciate this point from R1 and the opportunity to elaborate, as we can envision many readers will struggle to grapple with these seeming contradictions. In brief, both can be true at the same time because these two observations emerge from different processes.

Our point about 'asymptomatic' individuals is about population-level estimation: Because most individuals have few symptoms, likelihood-based/OLS estimates of cross-domain associations are disproportionately influenced by the large asymptomatic group that makes up the plurality of cases in population-level data. As noted above, this corresponds to ~70% of the population when considering symptoms currently being experienced.

In contrast, the increased probability of receiving an additional diagnosis after a first diagnosis reflects selection and ascertainment: once an individual meets criteria for an initial disorder they are enriched for higher underlying liability and are more likely to be under clinical observation, which increases detection of additional conditions (i.e., an applied example of the well-known Berkson's bias statistical paradox). Thus, comorbidity parameter estimates can be driven by the low-symptom majority in the full sample, and clinically observed diagnoses can still be more frequent among those already diagnosed. Even though comorbidity and multimorbidity occur in the population, they are statistically rare *overall* and thus are not as heavily weighted in the OLS regression model.

Because we anticipate other readers will share R1's view, we generated an Appendix (Appendix A) that explains in greater detail how the linear model is dominated by asymptomatic cases, and clinic-level inferences are inherently driven by ascertainment bias, meaning that the two sets of inferences are being driven by different pieces of information. For convenience, we have appended this new Appendix to the end of this document. We direct readers to this Appendix in the revised manuscript:

> **<u>p. 5</u>:** *"It may initially seem counterintuitive that we are arguing simultaneously (1) that people are statistically more likely to be diagnosed with a third mental disorder if they are diagnosed with two and (2) that covariance between mental disorders is driven by nomorbidity, or a lack of comorbidity. In Appendix A, we detail the specifics of how the linear model is influenced by the highest-density region of the data space, which can preclude inferences about comorbidity (high levels of two or more forms of psychopathology) when the data are largely composed of asymptomatic cases."*

<u>R1 #5</u>: A mention of the age onset of externalizing and internalizing disorders in youth at the ages sampled in ABCD study (perhaps as described in the DSM or from another source/research) would be helpful for contextualizing your results. Would we expect a certain percentage of kids at this age to have more severe or symptomatic internalizing

or externalizing symptom burden (or both)?

Response: We elected not to address the issue of age of onset of psychopathology in youths because this contextual information is unnecessary considering our demonstration that the nature of the nomorbidity phenomenon is distributional, not developmental.

Several of R1's comments allude to generalizability concerns regarding our use of youth data that may differ from conclusions drawn had we used adult data (R1 #5, 6, 8). In response to this general concern, we have now analyzed data from the adult caregivers of the youths in the ABCD Study, who completed the Adult Self-Report, the counterpart to the Child Behavior Checklist (results below).

Table 1.
Results from 1-change point models that regressed higher-order psychopathology dimensions onto one another among adults.

		**Change point Location (T1)**			**Slopes**							
					**B1**				**B2**			
IV	DV	T1	SE	R-hat	B1	SE	R-hat	% increase in DV	B2	SE	R-hat	% increase in DV
Internalizing	Externalizing	7.73	0.04	1.01	0.17	0.00	1.01	19	0.05	0.00	1.03	5
Externalizing	Internalizing	4.01	0.36	1.83	0.25	0.01	1.84	28	0.07	0.00	1.86	7
Attention Problems	Externalizing	3.24	0.05	1.03	0.32	0.00	1.03	38	0.10	0.00	1.04	11
Externalizing	Attention Problems	3.52	0.01	1.00	0.30	0.00	1.01	35	0.06	0.00	1.03	6
Attention Problems	Internalizing	3.46	0.05	1.02	0.32	0.00	1.01	38	0.09	0.00	1.04	10
Internalizing	Attention Problems	8.18	0.04	1.00	0.17	0.00	1.01	19	0.04	0.00	1.01	4
Thought Problems	Externalizing	3.39	1.14	1.20	0.46	0.04	1.21	58	0.14	0.01	1.14	15
Externalizing	Thought Problems	4.44	0.10	1.07	0.29	0.00	1.06	33	0.07	0.00	1.03	8
Thought Problems	Internalizing	1.50	0.01	1.00	0.63	0.00	1.00	89	0.17	0.00	1.00	18
Internalizing	Thought Problems	9.13	0.03	1.01	0.18	0.00	1.00	20	0.05	0.00	1.01	5
Attention Problems	Thought Problems	3.75	0.03	1.01	0.32	0.00	1.01	37	0.10	0.00	1.02	10
Thought Problems	Attention Problems	1.50	0.01	1.00	0.58	0.00	1.00	78	0.14	0.00	1.00	15

Note. 0- and 2-change point models were less viable than 1-change point models per the leave-one-out information criterion and R^2. Full results, including alternative change point models, are summarized in Table C9.

As can be seen in the table above, results from adults mirror those reported previously – the magnitude of the pre-change point slope far exceeds that of the post-change point slope, the latter of which is only weak in magnitude. We have revised the manuscript to include the results in Appendix C (Table C9), and have summarized these findings in several places in the discussion section:

> **pp. 16-17**: *"Third, we relied on sum scores of youth psychopathology data. In sensitivity analyses, we examined caregivers' psychopathology data from the Adult Self-Report, the adult counterpart to the CBCL, and our findings mirrored those reported here (Appendix C, Table 9). Change point models indicated a preference for one (as opposed to zero or two) change points, and the pre-change point slopes were considerably stronger than the post-change point slopes. It is perhaps unsurprising that our findings replicated among adults because the adult data in the ABCD Study were highly skewed and kurtotic (Fig. B11, Tables B16-17). As demonstrated in our simulation study (Appendix E), any data are liable to the problems we demonstrated here: The associations among higher-order*

psychopathology dimensions – in youths and adults – are driven up with the inclusion of asymptomatic cases."

Given space constraints, and the redundancy in findings across youths and adults, we have elected to maintain a primary focus on the youth analyses in the main text, but underscore replication in adults and, therefore, generalizability in our conclusions across development. We sincerely appreciate these points from R1, who inspired us to carry out this extension to the adult data, which have considerably strengthened our evidence base for the notion that the mechanism driving our results isnot developmental but driven by the fact that we detected evidence of genuine nonlinearity.
With that said, it is also worth noting that R1's comment might imply that the children were too young and therefore our findings might not generalize to adults. Given that our findings did generalize to adults, we do not feel that it is necessary to provide more context around age of onset, particularly because we used a dimensional assessment that considered psychopathology that is age appropriate and not all that statistically rare among youths (e.g., crying, feels sad).

<u>R1 #6</u>: As a reader, I found that the use of quotations in the Introduction section of the manuscript occasionally interrupted the continuity of the text. Paraphrasing these points could help strengthen the author's own voice and argument early on.

<u>Response</u>: We appreciate this feedback but have elected to retain quotations given that we feel it is important to establish that we are characterizing widespread conclusions in the field, as opposed to setting up a straw man argument.

<u>R1 #7</u>: How were symptomatic levels defined? This term was used in the Abstract, but I am not sure how it is defined. I see how asymptomatic is defined (e.g., 3-5 symptoms), so I think symptomatic should also be operationalized.

<u>Response</u>: We have revised several sentences in the manuscript for clarity, specifically to detail what we mean by asymptomatic and symptomatic. For example, we revised "The relative rate of the pre-change point slope was 4.6 times larger than that of the post-change point slope (32 / 7 = 4.6), suggesting far stronger slopes among largely asymptomatic cases" to include "who endorse fewer than 2 symptoms". Because the line between asymptomatic and symptomatic is fuzzy, we have revised "symptomatic" to "higher", in relation to "extreme low (asymptomatic)." For example, the abstract now states: "We observed strong positive associations among psychopathology dimensions at relatively extreme low (asymptomatic) levels (e.g., when 3 to 5 symptoms were endorsed) and weak positive associations at higher levels." We have carried forward these revisions throughout the manuscript.

<u>R1 #8</u>: I think the authors need to make a better argument for why the ABCD dataset is an appropriate or fitting sample for this type of research question. There needs to be clearer motivation for studying this research question in children vs. adults. I think this could be addressed and explained in Methods and Introduction section of the manuscript.

Response: Thank you for the opportunity to clarify. It is worth noting that externalizing and internalizing were first discovered in children by Achenbach in the 1960s, around 35 or so years before they were first studied in adults. As such, we think that studying youth is ideal for this research question. In terms of ideal youth data, the ABCD Study contains data from the largest cohort of youth in the US followed across development, for whom we have annual data on psychopathology, facilitating replication across four waves of data.

To make these points clear, we have revised the manuscript to justify our use of a nationally representative (-ish) sample of youth:

> **p. 5:** *"Here, we focus on youth who took part in the Adolescent Brain Cognitive Development Study (or ABCD[38]) given that externalizing and internalizing were first observed among youth[15] and because epidemiologic data are often used to characterize the structure of psychopathology[6]. The large size of the ABCD Study affords among the best powered examinations of the interface between higher-order psychopathology dimensions in youth."*

Further, given the inclusion of results from adult caregivers that replicate our findings from youth, we now feel that there is less of a need to motivate the research question in youth versus adults given the developmental stability of our findings.

> **R1 #9:** A citation is needed for line 81-82: "The CBCL is among the most widely used assessments of youth psychopathology."

Response: We have now provided a citation to support our statement, and we have also revised that statement to provide more justification for it. For clarity, we have also revised our statement to clarify that the CBCL is among the most widely used *broadband* assessments of youth psychopathology, to acknowledge that there are commonly used assessments for narrower sets of phenomena (e.g., externalizing).

> **p. 6:** *"We used the caregiver-reported Child Behavior Checklist (CBCL[40]), a 113-item assessment of psychiatric symptoms. The CBCL is among the most widely used broadband assessments of youth psychopathology, with over 10,000 publications reporting its use across over 50 different countries[41]."*

> **R1 #10:** I was curious why the authors used the CBCL over other mental health measured in the ABCD study protocol, including the diagnostic interviews (K-SADS)?

Response: We chose the CBCL over the K-SADS because the K-SADS includes a skip-out assessment structure whereby participants are administered a limited set of questions (gating questions) probing certain symptoms for a given disorder and are not fully assessed for the disorder if they do not endorse the gating questions. In turn, there is extensive missingness that is conditional on symptom endorsement.

Researchers who use the K-SADS data are faced with several choices. One option in using the data is to listwise delete on the basis of missingness, or to analyze complete cases. This approach is problematic when rates of missingness exceed 5% (Liu & De, 2015). In ABCD, missingness far

exceeds 5% given that participants skip out of many disorder-based modules. Another option is to substitute missing values with zeros (i.e., "imputation with zeros"), assuming that participants would not endorse symptoms in a given disorder's module if they didn't endorse the gating questions. On that front, this approach makes a strong assumption about "true" zeros. Further, although this approach preserves the total sample size for analyses, populating the data with many zeros induces spurious correlations among disorders (e.g., McBride et al., 2023).

In the context of this study, autopopulating the data with zeros is bound to generate even more highly positively skewed (or zero-inflated) disorders than we see for CBCL dimension scores. Symptom-based assessments without a skip-out structure give participants all possible opportunities to endorse symptoms, whereas skip-out structures seriously increase the proportion of zeros in the data.

Given these issues, we elected not to use the K-SADS data. Nevertheless, in light of our new simulation study (summarized in Appendix E, and in response to R2 #3), we would anticipate even more dramatic evidence of nonlinearity had we used the K-SADS data given the immense zero-inflation in the data.

References

Liu, Y., & De, A. (2015). Multiple imputation by fully conditional specification for dealing with missing data in a large epidemiologic study. *International Journal of Statistics in Medical Research, 4*(3), 287–295.

McBride, O., van Bezooijen, J., Aggen, S. H., Kendler, K. S., & Fried, E. I. (2023). Quantifying skip-out information loss when assessing major depression symptoms. *Journal of Psychopathology and Clinical Science*, *132*(4), 396.

<u>R1 #11</u>: How would the results potentially change if the authors used multiple mental health measures specific to certain diagnoses rather than using sub scores of the same survey? (e.g., Conners). Did the authors consider using the sub-scales within the internalizing and externalizing CBCL scores (e.g., Anxious/Depressed, Aggressive Behavior)?

<u>Response</u>: Thank you for this interesting question. ABCD does not include thorough assessment of broadband psychopathology via other assessments, with the exception of the K-SADS. For instance, ABCD does not include the Conners.

Moreover, the results of our simulation study (summarized in Appendix E, and in response to R2 #3) show that the problem we demonstrate in this manuscript is not driven by content coverage of psychopathology per se, as much as it is driven by the data distributions, their bivariate distributions, and whether there are true nonlinear relationships among the variables. To the extent that assessments of psychopathology are extremely positively skewed, which is often the case in community-based samples, they are subject to the same problems we demonstrate here. We have revised the future directions section in the Discussion to encourage replication efforts using novel assessments:

<u>p. 6</u>: *"Readers interested in the interface among lower-order psychopathology dimensions within a higher-order dimension (i.e., anxiety and depression within internalizing) are*

directed to Table C8 in Appendix C, where we show the results for all change point models based on CBCL subscales. Briefly, results were consistent with what we report here, with few exceptions: Across models, the 1-change point model was superior to others, and pre-change point slopes were far stronger than post-change point slopes."

p. 19: *"We welcome investigations of nonlinearity among samples with more representation of severe levels of psychopathology, such as in clinical or mixed community-clinical samples, and differing assessments of psychopathology, including ones that assess finer-grained phenomena than we consider here. Although not of focus here, it is possible that the dimensionality of narrower phenomena is also driven by asymptomatic cases, and that higher-order psychopathology dimensions may appear more multidimensional at higher levels of psychopathology. For instance, perhaps depression and anxiety are most overlapping at low levels and less overlapping at higher levels (Appendix C)."*

R1 #12: I believe there needs to be a stronger explanation in the text for starting with internalizing and externalizing sub-scales and then moving to thought problems and attention problems scales.

Response: We have now expanded upon our justification for including Thought Problems and Attention Problems scales.

p. 6: *"We also considered two subscales that contain items not included in Externalizing and Internalizing, Attention Problems and Thought Problems (coefficient omegas were .86 and .70, respectively), because they map onto broad transdiagnostic dimensions in modern classification frameworks[18,42]. In addition to externalizing and internalizing spectra, modern frameworks also include neurodevelopmental[43] and psychosis[42] (or thought disorder) spectra, which map broadly onto attention problems and thought problems, respectively. As the field incorporates lesser studied higher-order psychopathology dimensions into hierarchical frameworks of psychopathology, we felt that broadening our focus to include these dimensions would be of relevance to the field."*

We began with higher-order dimensions because they are most typically the focus of studies that model the structure of psychopathology, as well as studies that use the CBCL; the subscales are more rarely reported. Further, we situate our study in the context of the *p*-factor literature, which argues for a *p*-factor in light of the frequently observed moderate covariation among higher-order psychopathology dimensions.

To address R1's point, though, we have now included results that regressed subscales within externalizing and internalizing onto one another, to characterize associations among lower-order psychopathology dimensions. These results are now reported in Appendix C (Table C8). In short, with few exceptions, findings for lower-order dimensions were consistent with the findings we reported for higher-order dimensions.

Given that the CBCL does not include many lower-order dimensions – only two for externalizing and three for internalizing – we do not think the CBCL is the optimal measure to characterize associations among lower-order psychopathology dimensions, but look forward to extensions of our work in alternative dimensional assessments that thoroughly characterize finer-grained psychopathology dimensions. Given space constraints, the CBCL's limited coverage of finer-

grained psychopathology dimensions, and the field's general interest in higher-order dimensions, we have included these findings in the Appendix and have referenced the findings in a limited manner in the main text:

> **p. 6**: *"Readers interested in the interface among lower-order psychopathology dimensions within a higher-order dimension (i.e., anxiety and depression within internalizing) are directed to Table C8 in Appendix C, where we show the results for all change point models based on CBCL subscales. Briefly, results were consistent with what we report here, with few exceptions: Across models, the 1-change point model was superior to others, and pre-change point slopes were far stronger than post-change point slopes."*
>
> **p. 19**: *"We welcome investigations of nonlinearity among samples with more representation of severe levels of psychopathology, such as in clinical or mixed community-clinical samples, and differing assessments of psychopathology, including ones that assess finer-grained phenomena than we consider here. Although not of focus here, it is possible that the dimensionality of narrower phenomena is also driven by asymptomatic cases, and that higher-order psychopathology dimensions may appear more multidimensional at higher levels of psychopathology. For instance, perhaps depression and anxiety are most overlapping at low levels and less overlapping at higher levels (Appendix C)."*

> **R1 #13:** In line 181-182, the authors describe the percentage of participants at wave 1 who had nonclinical levels of internalizing and externalizing and the percentage of the sample who exhibited some combination of at-risk or clinical levels of both internalizing and externalizing, but the percentage does not add up to 100%. What about the other percentage of participants?

Response: Thank you for the opportunity to clarify. We emphasized only the symptomatic and multimorbid estimates in the main text given our focus on comorbidity. We refer readers to Fig.1, which includes estimates for cases that were (1) at-risk and above for internalizing and (2) at-risk and above for externalizing, where estimates across these classification groups sum to 100%.

> **R1 #14:** One thought I had while reading the Discussion section of the manuscript was, is there less comorbidity seen in the more symptomatic cases because there are less people with high symptom burden and more people with lower symptom burden in the sample? What if there was more variability in the higher end of the scale? What if it was an older sample, or a more clinical sample?

Response: Thank you for this comment, which ultimately strengthened our paper considerably. In response to reviewer comments regarding the fact that we analyzed data from youth and not adults, we analyzed data from caregivers. In the ABCD Study, the primary caregiver who attended the data collection session(s) with the youth participant completed the adult counterpart to the Child Behavior Checklist, the Adult Self-Report.

As can be seen in Appendix C (Table C9), with an abbreviated version at the end of this document, our results replicated among adults, with the change point models favoring 1 as opposed to 0 or 2 change points. For example, when we regressed internalizing onto externalizing in youth at

wave 1, the pre-change point slope was considerably larger than the post-change point slope (B_1 = .36, % increase in DV = 44; B_2 = .06, % change in DV = 6). Similarly, in adult caregivers at wave 1, the pre-change point slope was considerably larger than the post-change point slope (B_1 = .25, % increase in DV = 28; B_2 = .07, % change in DV = 7). These patterns across youth and their adult caregivers held for all pairwise comparisons of higher-order dimensions (i.e., internalizing, externalizing, attention problems, thought problems).

Given these findings, we revised the manuscript's discussion section to include a summary of the findings and refer readers to Appendix C, which includes all tabled results. We elected to report these results briefly given space constraints. The revised discussion reads as follows:

> **<u>pp. 17</u>:** *"Third, we relied on sum scores of youth psychopathology data. In sensitivity analyses, we examined caregivers' psychopathology data from the Adult Self-Report, the adult counterpart to the CBCL, and our findings mirrored those reported here (Appendix C, Table 9). Change point models indicated a preference for one (as opposed to zero or two) change points, and the pre-change point slopes were considerably stronger than the post-change point slopes. It is perhaps unsurprising that our findings replicated among adults because the adult data in the ABCD Study were highly skewed and kurtotic (Fig. B11, Tables B16-17). As demonstrated in our simulation study (Appendix E), any data are liable to the problems we demonstrated here: The associations among higher-order psychopathology dimensions – in youths and adults – are driven up with the inclusion of asymptomatic cases."*

In the revision, we have also emphasized that we had a considerable number of cases (i.e., *n* = 257, or 2.2%) that are clinically severe for both externalizing and internalizing, suggesting that extremes of the bivariate distribution for externalizing and internalizing were adequately represented.

> **<u>p. 17</u>:** *"Fourth, given the highly positively skewed nature of our psychopathology dimensions, the ABCD data may be an inadequate representation of psychopathology at clinically severe levels. We offer several counterpoints to this potential conclusion. Although nonclinical cases reflected the plurality of data in the ABCD Study (Fig. 1), we had an appreciable amount of clinically severe levels of externalizing and internalizing, with 2.2% of youths (n = 257) falling into this category. Moreover, when we forced two-change point models, we did not find a change point at severe levels of psychopathology dimensions and did not find evidence of increasingly strong associations at severe levels (Appendix C). In one instance, when we regressed externalizing onto internalizing in the adult data, we detected a second change point at a score of 30 (Appendix C, Table 9). Still, the three slopes detected in this model suggested a decreasing association between externalizing and internalizing as a function of severity (B_1 = .29, B_2 = .12, B_3 = .04)."*

Further, the alternative estimation methods – generalized additive mixed models – described in Appendix D allow for stronger associations to emerge at high levels of psychopathology, such as clinically severe levels. No such associations emerged. Taken together, we feel that our study was well-suited to identify an "uptick" in the associations among higher-order psychopathology dimensions at high levels, but no such evidence emerged. Still, we retain a discussion of this possibility in our revised Discussion section.

> **<u>p. 18</u>:** *"Along those lines, we used an alternate estimation method, generalized additive multilevel models (see Appendix C), that may be more sensitive to the upper tail of the distribution and still found no support for this hypothesis. Thus, we are not convinced that*

our 2-change point models were preordained to reject the notion that higher-order psychopathology dimensions become more strongly associated at severe levels[22]. Instead, there was no uptick for the third estimated slope, ruling out the possibility that comorbidity "emerges" at severe levels of psychopathology."

R1 #15: I think more discussion on the results in the context of the sample would be beneficial- this study is in children, yet the discussion doesn't necessarily focus on the comorbidity of mental health challenges in children vs. adults.

Response: Our response to R1 #14 overlaps with the issues raised here, so we focus here only on the requested revisions to the discussion section. Given that our findings replicated nicely across youths and adults, we have elected not to emphasize that our findings are limited to youths in the discussion section. Instead, we accordingly revised the discussion section to underscore the replication across youths and adults, to broaden our argument regarding the generalizability of our findings.

R1 #16: The Discussion (and Introduction) could benefit from section headings to guide the reader through the main arguments and improve the overall flow.

Response: We appreciate this suggestion, but we omitted section headings per journal formatting requirements.

R1 #17: Are the authors able to cite examples of previous work where comorbidity was driven by lack of mental health burden rather (i.e., nonmorbidity) rather than prevalence of multiple mental health challenges (i.e., comorbidity)? This research is alluded to but not necessarily discussed in detail.

Response: We have referenced work that has made adjacent points about psychopathology, but no study (to our knowledge) has examined the fact that psychiatric comorbidity is driven by nomorbidity.

pp. 4-5: *"As the basis of this claim, we borrow from a larger literature that suggests that the inclusion of asymptomatic (or undiagnosed) cases in covariance-based analyses of the structure of psychological constructs encourages their unidimensionality[34], or inflates the overlap among the phenomena of interest. Including asymptomatic cases produces dense network[35] structures and coherent (unidimensional) factor[34,36] structures that – although people assume they capture comorbidity – are driven by people who endorse few, if any, symptoms[37]. That is, what might glue together symptoms is a lack of comorbidity, or nomorbidity. We extend these findings to examine associations among higher-order psychopathology dimensions, whose covariation is interpreted as being synonymous with psychiatric comorbidity, using innovative statistical modeling techniques."*

R1 #18: Are the authors able to comment on what they see as the reason for the

disconnect between the results of this research and what is happening in the clinic? The authors themselves said that there is an increased risk of a second mental health diagnosis after the first is received, but if comorbidity in research is driven by low burden cases, what is happening in the clinic to cause this comorbidity phenomenon in the real world? A discussion on this could be helpful to readers who are clinicians.

Response: We appreciate this point, which we summarized briefly in our opening summary of the major changes we made to the manuscript. We acknowledge the tension between the two arguments you mention, (1) that comorbidity is a real phenomenon and (2) that covariance is driven by low burden cases. Note that we revised R1's statement to say "covariance is driven by low burden cases" as opposed to "comorbidity" because our findings emphasize that covariance and comorbidity are not interchangeable concepts, even though psychopathologists often use covariance-based measures (e.g., the correlation) to make claims about comorbidity.

Also, in the revised manuscript, we have included an appendix (Appendix A) that outlines the specifics of the ordinary least squares (OLS) model and the fact that it is dominated by high-density regions of the data space. In the case of community data, coefficients from the general model are driven by low burden cases, or asymptomatic cases. In contrast, comorbidity observed in the clinic is conditioned on experience of at least some symptomatology, reflecting clinical ascertainment. Here, we are not advocating that either observation is inherently wrong or invalid – they are simply describing two different things altogether.

The Appendix A we generated is appended at the end of this document. We wrote it with clinicians in mind, bridging the complexities of the OLS model with plain-language statements about how the OLS is sensitive to asymptomatic cases. We appreciate this suggestion to comment on this seemingly paradoxical set of observations and hope that the appendix is useful to clinicians and readers less familiar with the intricacies of statistical models.

> **p. 5:** *"It may initially seem counterintuitive that we are arguing simultaneously (1) that people are statistically more likely to be diagnosed with a third mental disorder if they are diagnosed with two and (2) that covariance between mental disorders is driven by nomorbidity, or a lack of comorbidity. In Appendix A, we detail the specifics of how the linear model is influenced by the highest-density region of the data space, which can preclude inferences about comorbidity (high levels of two or more forms of psychopathology) when the data are largely composed of asymptomatic cases."*

R1 #19: Text for Fig.2 was blurry and hard to read, especially the white text on the yellow background.

Response: Thank you for this feedback. We have revised the figures for clarity. With that said, the PDF rendering of the figures contributed to them being blurry, whereas we have generated high resolution .tiff files that should not be blurry when they are manipulated during proof production.

Reviewer 2

R2 #1: Using the ABCD data, the authors examine nonlinear relations between

internalizing and externalizing symptoms to provide evidence that comorbidity between internalizing and externalizing (and symptoms generally) is primarily driven by the absence of symptoms.

I find the paper to be interesting and novel. The authors appear to provide sufficient rationale for the Bayesian approach and provide supplemental analysis for alternative approaches. I had a few comments, questions, and suggestions that I hope the authors find helpful.

Response: Thank you for your feedback. We have responded to each of your comments below.

R2 #2: Given some of the authors main points regarding the need to go beyond the GLM, with a particular critique of the common factor model, I was surprised there was not more conversation about similar issues that have already been examined when the common factor model is considered within the IRT literature. The authors do cite one of the articles that came to mind:

78. Wall MM, Park JY, Moustaki I. IRT modeling in the presence of zero-inflation with 572 application to psychiatric disorder severity. Appl Psychol Meas. 2015;39(8):583-597.

But the reference to this work is quite brief: "Such an estimate is not only an oversimplification of the true population relationship, but a biased estimate of it"78–81

Response: We have revised the manuscript to expand upon our discussion of the IRT literature that has grappled (at least somewhat) with the issues we raise here, specifically by citing several additional papers:

> **pp. 15:** *"Such an estimate is not only an oversimplification of the true population relationship, but a biased estimate of it[78,82–86]. Specifically, we showed that these estimates are upwardly biased, over characterizing the statistical association between higher-order psychopathology dimensions at nonclinical levels of psychopathology, and particularly at clinical levels. Because the assumptions of the linear model were extremely violated in these data (Figs. A9-10), we conducted a simulation study (Appendix D) to show that the linear model produces a biased, and often extremely misleading, estimate of the population association between two psychopathology dimensions in the presence of nonlinearity under many conditions."*

In particular, we now cite the following papers:

Wall MM, Park JY, Moustaki I. IRT modeling in the presence of zero-inflation with application to psychiatric disorder severity. *Appl Psychol Meas*. 2015;39(8):583-597. doi:10.1177/0146621615588184

Azevedo CLN, Bolfarine H, Andrade DF. Bayesian inference for a skew-normal IRT model under the centred parameterization. *Comput Stat Data Anal*. 2011;55(1):353-365. doi:10.1016/j.csda.2010.05.003

Reise SP, Rodriguez A, Spritzer KL, Hays RD. Alternative Approaches to Addressing Non

Normal Distributions in the Application of IRT Models to Personality Measures. *J Pers Assess*. 2018;100(4):363-374. doi:10.1080/00223891.2017.1381969

Reise SP, Du H, Wong EF, Hubbard AS, Haviland MG. Matching IRT Models to Patient-Reported Outcomes Constructs: The Graded Response and Log-Logistic Models for Scaling Depression. *Psychometrika*. 2021;86(3):800-824. doi:10.1007/s11336-021-09802-0

This comment overlaps in themes explored with several subsequent comments by R2, so we will continue to address those themes as they arise.

R2 #3: At least at one point in the manuscript, the authors seem to suggest that psychology has not really considered the issues the authors are trying to highlight regarding distributions of the data and base rates and implications for covariance and so on, e.g., "Covariance-based similarity measures are dramatically influenced by the distributional qualities (e.g., base rates, skewness) of the variables of interest64, an issue that has been better appreciated in other fields (e.g., ecology, computer science, engineering)." And I would generally agree with this claim.

However, there has been several within the IRT literature that have made similar points when discussing the precursor to covariation between two constructs, which is how to estimate variation for one construct, which in itself is focused on covariation among indictor items for a given construct. Within IRT, this often involves deriving some theta score on some construct, and this theta is usually derived using the common factor model critiqued by the authors and the assumptions that go with it (e.g., linearity within a logistic scale assuming proportional odds within a graded response model for ordinal items).

Similar to the authors critiques, many within the IRT literature have noted the issue of the "quasi trait" or unipolar nature of many constructs relevant to psychopathology (e.g., depression) and have tried to come up with alternatives.

For example, the Wall et al. paper cited in the manuscript uses zero-inflated mixture modeling, where IRT parameters are only estimated for the "traited" class (since Wall et al., like the authors here, are concerned about the impact of all the observations with no symptoms - a factor, after all, just captures covariation among a set of measures thought to assess a single construct).

I am also familiar with some additional alternatives to the common factor model (Reise, S. P., Rodriguez, A., Spritzer, K. L., & Hays, R. D. (2018). Alternative approaches to addressing non-normal distributions in the application of IRT models to personality measures. Journal of personality assessment, 100(4), 363-374. may be a helpful reference), such as Lucke's unipolar log-logistic model and Ramsay-Curve IRT and so on. Again, the individuals contributing to this IRT work seem to make points similar to the authors here, "More generally, we call for a reconsideration of uncritical adoption of the general linear model in psychopathology research, which often produces comorbidity-inference artifacts in community-level data, where most individuals do not exhibit clinically severe psychopathology. We encourage researchers to embrace nonlinearity and population heterogeneity and adopt conceptual and analytic frameworks that prioritize symptomatology over statistical correlation."

However, the rub is, best to my knowledge, when these alternatives are implemented (such as the Wall et al. approach) and the thetas are calculated, they give thetas that correlate very, very highly with the thetas obtained by the common, "flawed" approaches.

For example, per Reise et al., using the more complicated zero inflated mixture model advocated by Wall et al. will give a theta that correlates .994 with the "flawed" graded response model implied by the common factor model. I think the one exception (at least among those highlighted by Reise) is the log logistic model, where Reise found thetas from this approach "only" correlated with .72 with the graded response model estimates.

All to say - it seems that many of the issues that the authors are highlighting here in terms of covariation have been highlighted regarding variation within the IRT literature (which is also focused on covariation, just at the indicator level). For me, they are nearly identical issues - a factor model relative to IRT involves linking together, for psychopathology, a set of symptoms (where many people have 0 values) - and covariation between different types of psychopathologies (e.g., internalizing and externalizing when using a lumped approach) involves linking together scores based on a model (usually the common factor model).

In my opinion, I would think one would want to puzzle out how to best model variation within a construct (i.e., puzzle out how to best model covariation between indicators of a construct) before scrutinizing the covariation between constructs. Unfortunately, we haven't really done that as a field, and thus the importance of the current work to challenge commonly held beliefs about comorbidity.

That being said, I think the authors could more strongly tie in work conducted within IRT that have grappled with similar issues and the practical limitations discovered in this work (e.g., technically more correct models that produce nearly identical thetas) and the implications of such work to the current focus on covariation (e.g., perhaps the highly correlated thetas from say a zero-inflated Wall et al. mixture still has utility for the issues discussed here, since that approach would let you remove the "traited" from the others before proceeding to covarying with something else).

Indeed, given the current work has arguably a more satisfying empirical result compared to some alternatives highlighted in IRT (with due respect to everyone, I do not find it that satisfying, in most contexts, to trouble with a zero-inflated mixture model IRT to get thetas that correlate .994 with the graded response model), perhaps the current work has implications if one where to take a step back and consider how to best measure the constructs we seek to ultimately covary.

<u>Response</u>: Thank you for these comments. R2 explores several themes in one comment. To summarize:

1. Unipolar/non-normal IRT methods already exist.
2. Zero-inflated IRT methods also already exist.
3. Even when these methods are used, they have generated largely unsatisfying results because the “thetas” derived from competing methods correlate extremely highly.
4. It would be better to employ these methods to explore dimensionality within a construct.

We will address each of these arguments in sequence.

1. **Unipolar/non-normal IRT methods already exist:** R2 stated: “I think the authors could more strongly tie in work conducted within IRT that have grappled with similar issues and the practical limitations discovered in this work (e.g., technically more correct models that produce nearly identical thetas).” As described in R2 #2, we have now more thoroughly cited this literature but respectfully disagree that the IRT models that exist are technically more correct than the models we use here. Those models and ours ask fundamentally different questions, with neither being inherently more valid than another.

   In particular, the IRT models that are developed with unipolar constructs in mind are currently only able to implement for unidimensional models, whereas the research question we pose here is inherently multidimensional, involving numerous higher-order psychopathology dimensions. That is, the IRT methods R2 cites are concerned with how to estimate a theta that reflects the true distribution of a single construct. Our research question surrounds what happens when you examine the relationship between thetas (or sum scores) in a community sample dominated by asymptomatic cases. Hence, we feel that the IRT methods mentioned by R2 are insufficient to address our study aims, and that the methods we used are more appropriate.

2. **Zero-inflated IRT methods also already exist:** Similarly, we are aware of existing zero-inflated IRT methods that separate the “traited” class from the “zero” class, though we perceive our method as having strong benefits above and beyond these approaches. Most notably, the Bayesian change point model allows for empirical identification of the change point(s) that demarcate where in the bivariate distribution the association between two psychopathology dimensions changes. Empirical identification of the change point represents a key advantage over zero-inflated models, which assume that “traited” and “non-traited” classes are distinguished by parsing the 0s from 1s and above.

   We could, for example, employ hurdle models to CFA data, where each factor is indicated by items that are simultaneous treated as Present/Absent and Level/Missing (also called semi-continuous models). But this does not allow us to *choose* the changepoint.

   In fact, our findings show that zero-inflated models are inappropriate here. Whereas zero-inflated models parse the 0s from all else, our change point models show that the cut-point in the distribution does not take place in between 0 and 1, and instead around 3-5 scale points depending on the analysis. As such, our method increments existing ones, in that it facilitates empirical identification of a cut point in the distribution, as opposed to assuming one *a priori*.

3. **Even when these methods are used, they have generated largely unsatisfying results because the “thetas” derived from competing methods correlate extremely highly:** R2 stated: “When these alternatives are implemented (such as the Wall et al. approach) and the thetas are calculated, they give thetas that correlate very, very highly with the thetas obtained by the common, "flawed" approaches.” We appreciate this point but think that the data we present here provide a strong counterpoint to R2’s generalization from the existing IRT literature that the status quo is sufficient because novel methods produce similar results.

   As alluded to by R2, previous work pertained to the dimensionality within a given construct (e.g., depression) as opposed to the covariance across constructs. Also, it is not entirely true that the literature surrounding novel methods (e.g., Christensen et al., 2022; Wall et al., 2015) produces findings that are essentially comparable to traditional methods. For instance, R2

described Wall's approach to parsing the zeros from the "traited class," and Christensen et al. (2022) first argued to parse these cases from one another in EFA for the very psychometric reasons we demonstrate in our study: When data are zero-inflated, the zeros dominate the dimensionality of an EFA solution and encourage a 1-factor solution. When all-zero responses are removed from the EFA solution, multidimensionality is better supported. The analog to our findings is that the zeros drive up covariance, and the change point models reveal weaker covariance at symptomatic levels.

> Christensen, W. F., Wall, M. M., & Moustaki, I. (2022). Assessing dimensionality in dichotomous items when many subjects have all-zero responses: An example from psychiatry and a solution using mixture models. *Applied Psychological Measurement*, *46*(3), 167-184.
>
> Wall, M. M., Park, J. Y., & Moustaki, I. (2015). IRT modeling in the presence of zero-inflation with application to psychiatric disorder severity. *Applied Psychological Measurement*, *39*(8), 583-597.

Next, R2 stated: "When these alternatives are implemented (such as the Wall et al. approach) and the thetas are calculated, they give thetas that correlate very, very highly with the thetas obtained by the common, "flawed" approaches." We appreciate this point but think that R2's claim is a bit strong for reasons we show below.

As a demonstration, we used a "brief form" of the CBCL by taking the items for externalizing and internalizing that are used in the Brief Problem Monitor, the short version of the CBCL. From there, we modeled unidimensional (1-factor) externalizing and internalizing dimensions, respectively, using a graded response model and the unipolar log-logistic model. The graded response model assumes an underlying normal distribution, whereas the unipolar log-logistic model does not.

R2 is correct that these two approaches yield highly correlated factor scores.

Table 2.
Correlations between factor scores derived from the graded response model and the unipolar log-logistic model.

	Spearman *r*	Pearson *r*
Externalizing	.99	.85
Internalizing	.99	.86

But these rank-ordered correlations do not tell the whole story. Below, we have displayed a scatter plot of the factor scores for both approaches. The red lines represent the linear relationship – as captured by the correlation – between factor scores. The blue lines represent LOESS curves that closely fit the data.

Redacted

As can be seen by comparing these two lines, the linear model is an oversimplification of the relationship between the factor scores derived from these competing IRT models: The association between factor scores is strong and positive at low levels of psychopathology, and the association weakens as a function of severity. In turn, the linear relationship dramatically overestimates the association between factor scores at high levels of psychopathology. We make huge prediction errors by relying on the correlation. These findings suggest that the correlation used to justify the lack of utility in the unipolar log-logistic (or non-normal IRT methods, more generally) is a mischaracterization of their relationship.

It is also worth noting that Reise et al. do make the point that theory, rather than model fit and the like, should guide decision making when choosing among competing IRT models (e.g., log-logistic, graded response model). Although there is no strong theory to guide the exploratory analysis we conducted here, thankfully we do have compelling statistical information in hand: Assumption checks that inform whether our data violate assumptions of the OLS model strongly indicate that OLS assumptions are violated. We summarized these results in the original submission (Appendix B, Fig.B9) but only showed data where we regressed externalizing onto internalizing. Now, we provide assumption checks for a comprehensive set of models (Appendix B, Fig.B10).

As can be seen below, the models we present substantially violate three assumptions of the OLS model – linearity, normality of residuals, and homogeneity of variance, all of which support the presence of nonlinear associations among psychopathology dimensions. As such, even though the OLS model (and the factor model, in turn) are widely popular in the psychopathology literature, they are often not defensible.

Redacted

Fig.B9. Assumption checking for the ordinary least squares model.

We have now revised the manuscript to include a standalone Fig.that explains the nature of the prediction errors associated with the ordinary least squares model:

Redacted

Fig. 3. Prediction errors generated by the ordinary least squares regression model in the presence of nonlinearity.

Note. Here, we depict the same data as presented in Fig. 2a, with an emphasis on the negative binomial models with 0- and 1-change points (the blue dashed and green solid lines, respectively), where we regressed externalizing onto internalizing. The 0-change point model corresponds to the ordinary least squares [OLS] regression model most typically used in the literature, albeit with a negative binomial link function as opposed to a Gaussian link function. By comparing the slopes from the OLS (0-change point) and 1-change point models, we see that the OLS estimate results in substantial prediction error (per the shaded regions of the plot) across levels of severity for internalizing, with much of the error occurring at clinical levels of severity. Blue shaded regions indicate where the OLS model is overestimating the association relative to what is predicted by the 1-change point model, and green shaded regions indicate where the OLS model is underestimating the association. Severity levels are denoted by the bar that ranges from nonclinical levels (yellow) to at-risk levels (orange) to clinical levels (red). For Internalizing, values lower than ~9 reflect nonclinical levels (per the T scores), between ~9 and ~14 reflect at-risk levels, and above ~14 reflect clinical levels (see Table C1 for a translation between raw scores and T scores). Thus, because of the considerable extent of nonclinical (and asymptomatic cases) in the data, the OLS estimate generally overstates the statistical association between externalizing and internalizing at nonclinical levels of internalizing, and especially at symptomatic and clinically severe levels of internalizing.

Finally, and most directly, the revised manuscript now includes a simulation study (described in Appendix E; for convenience, it is appended at the end of this document) that demonstrates that using an OLS model in the presence of a population mixture produces a biased estimate of the relationship between two variables. With the simulation being able to establish the “true” association between two variables, we can firmly conclude that the OLS is biased.

Redacted

Fig.E2. *Heatmaps of the OLS estimate given a 1-change point model with known pre- and post-change point slopes.*

Note. Here, we display the estimated OLS estimate (Pearson r) in each cell of the heatmaps given a known (true) 1-change point model with known pre- and post-change point slopes (or β_1 [x-axis] and β_2 [y-axis], respectively). The density distributions above the heatmaps display differing levels of the change point ($\tau = -1.5, 0, 1.5$). The color and darkness of the cells indicate the direction and extent of bias, whereby the correlation favoring the data to the left of τ is shaded blue and the data to the right of τ is shaded yellow. Where $\tau = 0$, the data are evenly distributed to the left and right of τ, therefore the cells are pale in color because the correlation returns the approximate midpoint of β_1 and β_2.

As summarized earlier: In short, we simulated data from a 1-change point model that establishes that – in the presence of nonlinear relationships among two variables – the OLS estimate (e.g., a slope from a simple linear regression, and by extension the Pearson's correlation) is associated with considerable bias. The simulation varied β_1 and β_2 over a grid from −0.8 to 0.8 (in increments of 0.2) and varied the τ over three values: −1.5, 0, 1.5. For every (β_1, β_2, τ) combination, we generated a dataset of size n=10,000.

In extreme conditions where the pre- and post-change point slopes are strong and equivalent in magnitude but differ by sign (e.g., B_1 = -.8, B_2 = .8), the OLS model estimates a null (B = 0) association. In other conditions, such as where there is a strong association pre-change point and a null association post-change point (e.g., B_1 = -.8, B_2 = 0), the OLS model estimates the average of these two associations (B = -.4), which characterizes neither the pre- or post-change point association and produces a misleading positive association post-change point when we can establish that one does not exist. Appendix E describes many such scenarios in detail.

Ultimately, our analysis of real psychopathology data also suggests that the OLS is not sufficient to characterize associations among higher-order psychopathology dimensions across severity levels in the population. That is, if OLS – the traditional method – and the change point models – the novel method – were redundant (e.g., a .994 correlation between thetas), they would produce identical results. Further, if there were a single estimate in the entire population, zero-, one- and two-change point models would reveal near identical slopes regardless of level of psychopathology. Our simulation study further supports this claim, showing that when pre- and post-change point slopes are identical, the OLS estimate is unbiased.

Our results do not align with these outcomes and instead show that there are substantial differences between the findings from the OLS model(s) and the change point model(s). The data we present here provide a strong counterpoint to R2's generalization from the existing IRT literature, that the status quo is sufficient because novel methods produce similar results.

Relevant revised quotes from the manuscript are as follows:

> **<u>p. 15:</u>** *"Such an estimate is not only an oversimplification of the true population relationship, but a biased estimate of it[78,82–86]. Specifically, we showed that these estimates are upwardly biased, over characterizing the statistical association between higher-order psychopathology dimensions at nonclinical levels of psychopathology, and particularly at clinical levels. Because the assumptions of the linear model were extremely violated in these data (Figs. A9-10), we conducted a simulation study (Appendix D) to show that the linear model produces a biased, and often extremely misleading, estimate of the population association between two psychopathology dimensions in the presence of nonlinearity under many conditions."*

> **<u>p. 17</u>:** *"Second, because we allowed for a change point in the associations between higher-order psychopathology dimensions, skeptics might argue that we artificially produced different slopes and, in turn, evidence of nonlinearity. Change point models do not inherently produce evidence of nonlinearity. Indeed, our simulation study (Appendix E) demonstrates that the change point model will generate estimates identical to the linear model when there is no evidence of nonlinearity. We observed evidence of this fact in our analysis of psychopathology data, as well. For instance, in the Gaussian 1-change point model that regressed externalizing onto internalizing (Appendix C), the pre- and post-*

change point slopes were almost identical to each other, and to the slope estimated in the 0-change point model. Thus, it is not always the case that modeling a change point will engender evidence of nonlinearity."

pp. 17: *"It is perhaps unsurprising that our findings replicated among adults because the adult data in ABCD Study were highly skewed and kurtotic (Fig. B11, Tables B16-17). As demonstrated in our simulation study (Appendix E), any data are liable to the problems we demonstrated here: The associations among higher-order psychopathology dimensions – in youths and adults – are driven up with the inclusion of asymptomatic cases."*

4. **It would be better to employ these methods to explore dimensionality within a construct:** We agree that it would be interesting to use varied quantitative methods to characterize nonlinearity and its effects on the dimensionality of individual constructs, but we disagree that it should be the first, or primary, focus of our efforts. Much attention has been paid to higher-order dimensions of psychopathology and their covariation, with the field being increasingly interested in "lumping" putatively distinct phenomena into increasingly broad transdiagnostic dimensions (e.g., general factor models like the "*p*-factor"). Along those lines, far more attention has been paid to lumping into broad, higher-order dimensions, and we believe our manuscript is a timely response to the field's current collective interest.

   Nevertheless, we agree with R2 that their described research question is an important one, and we have revised the manuscript to motivate researchers to explore this issue in future research:

   p. 19: *"We welcome investigations of nonlinearity among samples with more representation of severe levels of psychopathology, such as in clinical or mixed community-clinical samples, and differing assessments of psychopathology, including ones that assess finer-grained phenomena than we consider here. Although not of focus here, it is possible that the dimensionality of narrower phenomena is also driven by asymptomatic cases, and that higher-order psychopathology dimensions may appear more multidimensional at higher levels of psychopathology. For instance, perhaps depression and anxiety are most overlapping at low levels and less overlapping at higher levels (Appendix C)."*

> **R2 #4:** Related to point 1, I wasn't quite sure what the way forward is per the authors beyond the general conclusion is we should be doing something different than we are typically doing, and that different thing probably won't be limited to linear relations. For example, on line 283, the authors state "there may be no single, "true" structure of psychopathology in the population" - if this is true, what is the way forward for say DSM-6 or individuals who work and train in the applied branches of clinical psychology (e.g., see patients) and have to seek training in things like psychopathology and learn to yoke said psychopathologies to say evidence-based treatments?

Response: Thank you for this comment. Our intent was not to imply that nosology or clinical training should be abandoned, but rather to emphasize that the latent "structure" recovered from statistical models may be contingent on measurement, sampling, and modeling assumptions, and therefore should not automatically be treated as a unique population-level ground truth. We have

now added a paragraph that articulates some implications of our findings for DSM-6 and classification efforts more generally.

> **p. 16**: *"As the framers of the DSM-6 are charged with capturing the continuum (or dimensionality) of mental health and to explicitly consider severity and transdiagnostic features[96], our findings are highly relevant to current efforts to incorporate dimensionality into future psychiatric nosology[9,14]. In particular, our findings suggest that dimensional structures of psychopathology should be evaluated in relation to their target population and intended clinical use[97]. A structure estimated from covariance in a community sample may be useful for population screening or describing overall symptom burden, but it should not automatically be interpreted as evidence of clinical comorbidity, shared etiology, or treatment-relevant co-occurrence among symptomatic patients. In community samples overrepresented by asymptomatic cases, global covariance may primarily reflect shared absence of symptoms. Thus, studies used to support dimensional nosology should report the distributional qualities of the data (e.g., skewness, kurtosis), confirm that assumptions of the general linear model are met in their data, examine nonlinear associations among forms of psychopathology, and evaluate whether broad structures remain informative in the clinical range. Our data suggest that the general linear model results in overprediction of covariance (or so-called comorbidity) among psychopathology dimensions at clinically severe levels of psychopathology."*

R2 #5: Related to point 1 and 2, I am not quite sure what the authors are suggesting for alternative measurement models to constructs related to psychopathology, particularly in light to the existing IRT literature that went down this road and primarily found thetas that correlate really, really highly with standard approaches.

My read of the discussion is that I get reminded that comorbidity is probably driven by the zero cases (just as the factor loadings for a given construct's measurement model is probably driven by the zero cases on the indicators), and that other fields think of this better than we do - we have the p-factor after all - then on line 264 we seem to get to the measurement model issue and note the assumptions (as is done in Wall et al. and the other IRT papers I mention) and how things like average can be misleading (similar to points from the IRT literature, e.g., "it makes little or no sense to assert that a person has below-average level of addiction to alcohol or an above-average level of addiction to gambling", Lucke, 2015), then some info on how configural invariance across populations might be hosed, then some reasonable defense of study limitations, and then back to how we need to do something different, something nonlinear. But what do we do, exactly?

To put it another way, I have heard this (sort of) all before from the IRT literature, and I find the arguments compelling, but I have not found the nonlinear alternatives findings to be that satisfying - what is different here?

Response: We believe we largely addressed the theme of this comment in response to R2 #1-3, where we respectfully disagree that the IRT literature has settled this matter, and should be ignored by psychopathologists because the results are unsatisfying. Instead, we are offering a path forward, specifically by way of introducing a novel method that allows researchers to critically examine whether the OLS estimate typically implemented is appropriate and supported.

<u>R2 #6</u>: If there is to be revolution for how we view comorbidity, it would seemingly start with a revolution in our measurement models, but I am not quite sure what the authors have in mind here or how that would be different from what has been proposed and tested. (to be clear, I don't think it is the authors' charge to solve this, but perhaps something more concrete would be helpful).

In the meantime - what do we do? It seems a very practical implication is that if the field is going to continue to use the common factor model and extensions (e.g., sum scores), which seems to be a solid bet, the least we could do is add say a quadratic term when say regressing internalizing on externalizing to see how often the relation dampens as we get higher in X...do the authors agree with this? And again, if not this, what?

<u>Response</u>: With our open code, we are offering researchers a direct pathway forward: Analysis of skewed psychopathology data that violate assumptions of the OLS model using our proposed method. Simply adding a quadratic term may not be sufficient, as it assumes a specific functional form to the association between two variables. In our view, it is unnecessary to simplify the analytic approach to that extent given that we provide code to use a more appropriate method.

We have also revised the manuscript to suggest an analytic pipeline to researchers, which may or may not include our particular use of Bayesian change point models:

> **<u>p. 16</u>:** *"Thus, studies used to support dimensional nosology should report the distributional qualities of the data (e.g., skewness, kurtosis), confirm that assumptions of the general linear model are met in their data, examine nonlinear associations among forms of psychopathology, and evaluate whether broad structures remain informative in the clinical range. Our data suggest that the general linear model results in overprediction of covariance (or so-called comorbidity) among psychopathology dimensions at clinically severe levels of psychopathology."*

<u>Reviewer 3</u>

<u>R3 #1</u>: This manuscript examines patterns of associations between dimensions of psychopathology by challenging multiple assumptions espoused in dimensional models of psychopathology. The authors are very clear in their description of the problem. Whether others agree with the premise or not, the authors make cogent arguments based on transparent observations of historic and the analyzed data. If readers and reviewers react negatively to the work, I suspect that these arguments are hitting on fundamental assumptions that shake a compelling narrative, rather than the evidence. The supplementary material provides a great set of complementary analyses that address the low hanging comments that could be made. That said, I am challenged to find many areas of the manuscript to critique. I have some thoughts for the authors to consider, but I do not know that all may lead to a cleaner manuscript.

<u>Response</u>: We sincerely thank you for your constructive feedback and engagement with our work, and have addressed your comments below.

<u>R3 #2</u>: Perhaps one quibble is in terminology. The authors propose the concept of 'nomorbidity.' Is this premise based on the massive center of data with no endorsement of psychopathology, or the degree of association between the internalizing and externalizing dimensions? If the former, I see the naming. However, if the latter, I wonder if this space of the distribution would be better termed ~low-morbidity? That is, the associations are stronger at lower levels, but variability is needed in order to quantify the relation.

<u>Response</u>: Thank you for this question. We meant the former. To help make this clear to the reader, we have revised the manuscript to define 'nomorbidity' more clearly so that we make this important point clear.

> **<u>p. 3</u>:** *"At a given time, most people in the general population do not exhibit appreciable levels of symptomatology to warrant a psychiatric diagnosis. Around 70% of the population exhibits what we term 'nomorbidity' – wherein an individual is not diagnosed with a single mental disorder – when past-year diagnoses are assessed and 50% when lifetime diagnoses are assessed[3]."*

<u>R3 #3</u>: The authors rely on change point models to evaluate the nonlinear associations in the sample. The complement of GAMMS is complementary and show This is quite clear. The context of the work and focusing on dimensionality leads to an intuitive understanding of these results. Is there an alternative explanation that these relations may be better conceptualized as subgroups coming from different portions of the population? Is there a way of reconciling those different perspectives?

<u>Response</u>: We completely agree. We have summarized this possibility in two places in the manuscript.

> **<u>p. 13-14</u>:** *"Critically, our findings suggest that the p-factor appears largely irrelevant to clinical levels of psychopathology and the subpopulation of people who experience psychopathology in some significant form, which is inconsistent with the notion that it is a unitary dimension on which all humans can be placed[21,22]."*

> **<u>pp. 15-16</u>:** *"Ultimately, there may be no single, "true" structure of psychopathology in the population[87,88]. Instead, the assumption of population homogeneity may be inappropriate[88], and there may be discrete subpopulations of people that are differentially susceptible to psychopathology, have differential comorbidity patterns, and in turn have dramatically different structures of psychopathology. Related literatures support such a possibility: Self-esteem appears to have different factor structures – one unidimensional and one multidimensional – depending on the subpopulation of interest[89]. Further, the structure of intelligence appears moderated by ability level, with the strength of correlations among intelligence tasks decreasing as intelligence levels increase[90–92].*

> *With the considerable degree of asymptomatic cases in most large-scale epidemiologic samples in mind, it may be the case that there is a large class with a relative absence of diagnosed psychopathology for whom psychopathology appears to conform to a unidimensional solution[35,36,93], and one or more relatively symptomatic classes for which*

> *psychopathology appears multidimensional. We suspect that there is a subpopulation that comprises people with an exceptionally high threshold to experience and/or accurately report psychopathology, whose detection is obscured – even precluded – in most modern classification efforts[94] due to the widespread assumption of population homogeneity[95].”*

Still, we do not know that it is entirely possible to reconcile these competing perspectives – that there is a single structure of psychopathology in the population, or more than one – because the methods commonly used to study the structure of psychopathology inherently assume population homogeneity. For instance, conventional applications of factor analysis, network analysis, and item response theory models all assume that there is a single structure in the population at hand. Because of the dominant influence of factor analysis, in particular, on modern psychiatric classification, we hope that these findings add to the conversation and are hopeful that our findings motivate skepticism such that researchers do not inherently assume that population homogeneity holds.

> **R3 #4:** The data came from a community-based sample and a second change point was not identified in the models. Is it possible that a sample with a higher level of severity may show a second inflection point at higher levels of severity? Might this dovetail with the argument that there is not a uniform psychopathology structure in the population?

Response: We were interested in detecting an inflection point at high levels of psychopathology, such that the association between higher-order dimensions became stronger after the second change point (consistent with an inverse-U relationship). With that said, we did not find evidence of this possibility. Although our model fit indices did not support 2-change point models, it is worth noting that we forced a second change point and did not find evidence that the slopes increased after the second change point. Instead, they became even weaker, providing evidence against the notion that associations would be stronger at higher levels of severity.

In one instance (though we note that this observation was extremely rare in these data), when we regressed externalizing onto internalizing in the newly included adult data, we detected a second change point at a score of 30 (Appendix C, Table C9). Still, the three slopes detected in this model suggesting a decreasing association between externalizing and internalizing as a function of severity ($B_1 = .29$, $B_2 = .12$, $B_3 = .04$). Further, the GAMM models described in Appendix D further allowed for an increasing relationship at extremes of the bivariate distribution and did not find evidence for this possibility.

Given these data, we revised the manuscript to expand on our discussion of the data-driven counterparts to the argument that higher-order psychopathology dimensions covary increasingly strongly as a function of severity:

> **p. 18:** *“Fourth, given the highly positively skewed nature of our psychopathology dimensions, the ABCD data may be an inadequate representation of psychopathology at clinically severe levels. We offer several counterpoints to this potential conclusion. Although nonclinical cases reflected the plurality of data in the ABCD Study (Fig. 1), we had an appreciable amount of clinically severe levels of externalizing and internalizing, with 2.2% of youths (n = 257) falling into this category. Moreover, when we forced two-change point models, we did not find a change point at severe levels of psychopathology dimensions and did not find evidence of increasingly strong associations at severe levels (Appendix C). In one instance, when we regressed externalizing onto internalizing in the adult data, we detected a second change point at a score of 30 (Appendix C, Table 9).*

Still, the three slopes detected in this model suggested a decreasing association between externalizing and internalizing as a function of severity (B_1 = .29, B_2 = .12, B_3 = .04).”

R3 #5: For some normative anchoring, it could be helpful to link raw scores to percentiles or t-scores.

Response: In the revised manuscript, specifically in the Fig.notes, we now note the approximate scores that constitute “nonclinical”, “at-risk”, and “clinical” levels of externalizing and internalizing. We said “approximate” because, unfortunately, the raw scores cannot be precisely linked to T scores, such that a score of 10 on CBCL Externalizing is equivalent to a T score of a particular value. T scores take into account age and sex, so the same raw score is associated with a range of T scores as a function of age and sex. To provide a way to translate raw scores into T scores, we computed the average raw score at each possible value of T scores and report these data in Appendix C (Table C1). We refer readers to these data in the revised manuscript:

> **p. 6:** “For readers interested in T scores, Table C1 (Appendix C) contains a translation between raw scores and manual-based T scores for all scales across all waves.”

We also revised Figure notes to summarize information pertaining to T scores. For example, the Figure note for Fig. 2 states:

> **p. 34:** *“For Internalizing, values lower than ~9 reflect nonclinical levels (per the T scores), between ~9 and ~14 reflect at-risk levels, and above ~14 reflect clinical levels. For Externalizing, values lower than 12 reflect nonclinical levels, between ~12 and ~16 reflect at-risk levels, and above ~16 reflect clinical levels. The specific raw score that corresponds to a T-score varies as a function of age and sex. Table C1 (Appendix C) contains a translation between raw scores and T scores for all scales across all waves.”*
